# Stereoselective Synthesis of α-Disubstituted β-Homoprolines

**DOI:** 10.1021/acs.orglett.3c02891

**Published:** 2023-09-20

**Authors:** Arianna Quintavalla, Davide Carboni, Maria Simeone, Marco Lombardo

**Affiliations:** † Alma Mater Studiorum - University of Bologna, Department of Chemistry “G. Ciamician”, via P. Gobetti 85, 40129 Bologna, Italy; ‡ Center for Chemical Catalysis - C3, Alma Mater Studiorum - Università di Bologna, via P. Gobetti 85, 40129 Bologna, Italy

## Abstract

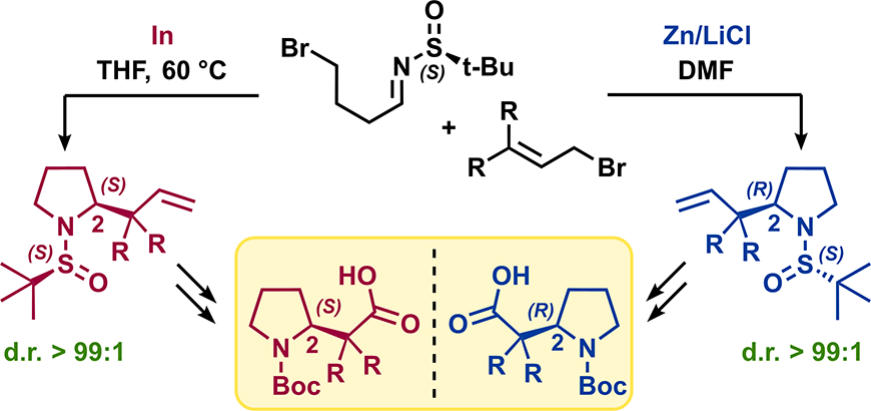

An efficient enantioselective synthesis of chiral α-disubstituted β-homoprolines was developed, starting with the stereodivergent allylation of chiral *N*-*tert*-butanesulfinyl imines derived from 4-bromobutanal with indium or zinc and using well-established and reliable synthetic transformations. This methodology allows the easy introduction of different substituents at the α-position of the pyrrolidine scaffold and is characterized by the possibility of switching the absolute configuration of the newly formed stereocenter either by changing the configuration of the *tert*-butanesufinamide chiral auxiliary or by using a different stereodivergent allylation protocol with the same auxiliary.

The field of therapeutic peptides has witnessed a remarkable evolution in the past several years, driven by an ongoing pursuit of innovative design, synthesis, and delivery strategies to address the inherent limitations associated with the use of peptides.^1^ Indeed, peptide drugs constituted a substantial portion of the pharmaceutical market in 2019, with 10 non-insulin peptide drugs among the top 200 drug sales, and the top three peptide drugs employed in treating type 2 diabetes.^1^ One of them, semaglutide, was recently approved also for chronic weight management in adults with general obesity,^2^ capturing the interest of newspapers and public opinion for its potential misuse as a weight loss aid.

The synthesis of bioactive peptides containing β-amino acids represents a promising strategy for preparing new medicinal chemistry entities with distinctive properties, because the insertion of unnatural amino acids into peptide sequences can modulate not only their conformations but also their biological activity and metabolic stability.^3^ β-Homoproline (β-Pro, **1**) has demonstrated intriguing properties in this context and has found compelling applications in medicinal chemistry. For instance, the replacement of natural proline (Pro) with β-Pro in the tetrapeptide endomorphin-1 (H-Tyr-Pro-Trp-Phe-NH_2_) significantly increased both the μ-opioid receptor affinity and the resistance to enzymatic hydrolysis.^4^ Interestingly, the analogue with l-β-Pro exhibited ∼20-fold greater activity than that with having d-β-Pro. Again, the replacement of Pro with β-Pro in the Pro-Leu dipeptide, a potential agent against cardiovascular diseases, displayed a 500-fold increase in the inhibitory activity of bradykinin cleavage by aminopeptidase and a complete stability to peptidases in kidney membranes after 24 h.^3a^

Despite the interesting potential applications of β-Pro in the production of new drugs, its use has remained relatively unexplored in the chemical literature, most probably due to the very few synthetic methods available for its preparation in enantiopure forms.^5a−5f^ Some chiral homoprolines and derivatives have been proposed as organocatalysts,^5g−5j^ in a manner analogous to the use of proline.^5k,5l^ More recently, a few syntheses of β-homoproline analogues, possessing supplementary substituents on the pyrrolidine ring, have also been proposed.^6^

Another intriguing strategy for modulating the aggregation and self-assembly of oligopeptides involves the insertion of stereochemically constrained amino acids in the peptide sequence, allowing protein secondary structures to be modeled using short peptides.^7^ α-Aminoisobutyric acid (Aib, **2**), the simplest unnatural achiral amino acid possessing a quaternary α-carbon atom, plays a crucial role in controlling peptide conformations through the Thorpe–Ingold effect, promoting helical folding in both synthetic and natural oligopeptides.^8^

Surprisingly, only a few synthetic procedures have been reported so far for the preparation of conformationally constrained α-disubstituted β-homoprolines (**3**), and invariably in racemic form (Figure 1).^9−13^

**Figure 1:**
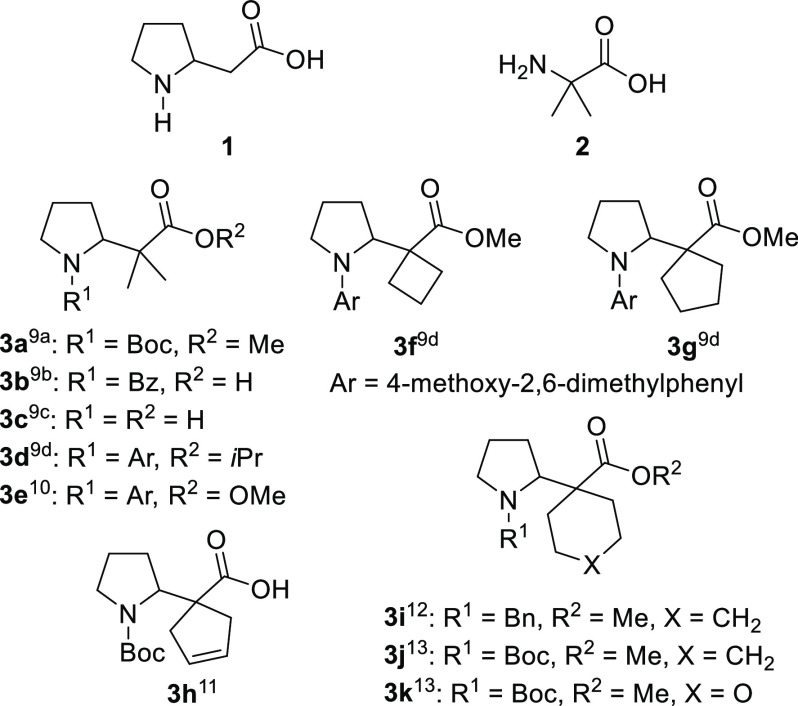
Structures of β-homoproline (**1**), α-aminoisobutyric acid (**2**), and α-disubstituted β-homoprolines (**3a**–**k**).

Here we present a novel and straightforward method for the enantioselective synthesis of a family of chiral α-disubstituted β-homoprolines, which allows one to afford the desired products in both enantiomeric forms, using readily available and cost-effective reagents and exploiting well-established and reliable synthetic transformations. Amidst the plethora of methodologies for the construction of nitrogen-containing heterocycles,^14^ the addition of organometallic reagents to chiral sulfinimines, followed by the intramolecular cyclization of nitrogen on an opportunely positioned leaving group, appears to be particularly appealing. This strategy was originally pioneered by Ruano for the synthesis of optically pure 2-(1-hydroxybenzyl)piperidine and pyrrolidine^15a^ and later found broad application in the construction of chiral pyrrolidine scaffolds (**5**) from 4-halobutanal-derived chiral sulfinimines **4** (Scheme 1).^15^

**Scheme 1:**
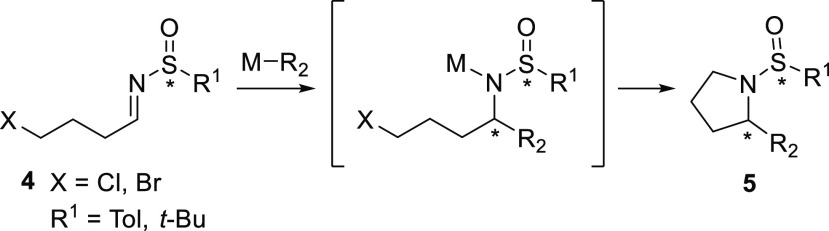
Synthesis of Pyrroldines from 4-Halobutanal-Derived Chiral Sulfinimines

In 2002, Ellman reported the addition of lithium enolates to chiral sulfinimines.^16^ Unfortunately, when we tried this reaction on chiral sulfinimines derived from 4-bromobutanal (**4a**), only simple acetate-derived enolates in the absence of ClTi(O*i*Pr)_3_ gave acceptable yields (40–50%), albeit with low diastereoselectivities (∼75:25). As lithium enolates gave unsatisfactory results, we decided to investigate the possibility of using allylation reactions for the stereoselective introduction of a double bond on the pyrrolidine scaffold, because simple functional group manipulations (FGI) would allow transformation of the terminal alkene moiety in the desired carboxylic acids.

The allylation of chiral sulfinimines with allylindium reagents was reported for the first time by Yus and Foubelo in 2004^17a−17c^ and extended to prenylation by González-Gómez in 2013.^17d^ The same authors further investigated this synthetic strategy, applying it also to the synthesis of pyrrolidine and piperidine scaffolds.^17e−17h^ In 2018, Guo reported the allylation of chiral sulfinimines with allylzinc reagents for the construction of indolines and tetrahydroquinoline derivatives.^18a^ Subsequently, Zhu employed the same procedure for the preparation of cyclic sulfinamides.^18b^

We started our investigation by examining the prenylation reaction of chiral sulfinimine (*S*)-**4a**, using magnesium, zinc, and indium, in THF, DMF, or water as the solvent, with or without additives (Table 1). The preformed Grignard reagent was added to a solution of the imine in THF at −78 °C. After 1 h, the complete disappearance of the imine was detected by TLC analysis, and the product was confirmed to be open adduct **8a** through ^1^H NMR analysis of a quenched sample. Cyclized product **7a** was quantitatively obtained with a diastereomeric ratio of 96:4 by simply allowing the reaction mixture to warm to 0 °C in 2 h (entry 1). The one-pot reaction protocol with indium and prenyl bromide **6a** (entry 2), as established by Yus and co-workers,^17^ yielded open product **8a** as a single diastereoisomer with a very high conversion. Crude open product **8a** can be very conveniently cyclized using LiHMDS in THF at rt for 1 h, affording the desired product **7a** in quantitative yield while maintaining the stereochemical integrity. Once the compound was cyclized, we confirmed by ^1^H NMR analysis that magnesium and indium favored the formation of the same diastereoisomer. When zinc was used under one-pot Barbier conditions, using THF as the solvent (entry 3), once again the conversion was quantitative at 0 °C after 5 h. In this instance, the crude mixture predominantly contained open adduct **8a** (80:20), even after the mixture had been stirred for 12 h at room temperature.

**Table 1:**
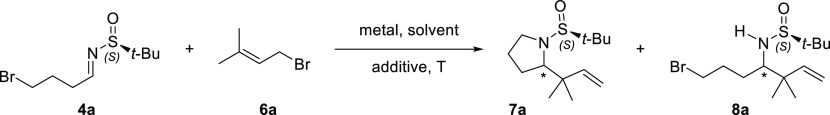
Prenylation of Imine (*S*)-**4a**^a^

entry	metal	solvent	additive	*T* (°C)	*t* (h)	conversion (%)^b^	**7a**:**8a**^b^	dr^b^,^c^
1	Mg	THF	–	–78/0	1/2	>99	>99:1	96:4
2	In	THF	–	60	6	>99	>1:99	>99:1
3	Zn	THF	–	0/rt	5/12	>99	20:80	90:10
4	Zn	DMF	–	0/rt	5/12	>99	80:20	55:45
5	Zn	THF	LiCl	0/rt	5/12	>99	40:60	60:40
6	Zn	DMF	LiCl	0/rt	5/12	>99	70:30	>1:99
7	Zn	H_2_O	NH_4_Cl	rt	2.5	89	70:30	67:33

a Reactions run on 1 mmol of **4a**, using 1.5 mmol of **6a**, 1.5 mmol of metal, and 1.5 mmol of an additive.

b Determined by ^1^H NMR analysis of crude reaction mixtures.

c The diastereomeric ratio (dr) refers to **7a** or **8a**; when a mixture of **7a** and **8a** was obtained, the dr was calculated on **7a** after cyclization of the crude mixture with LiHMDS in THF.

Once again, after the complete cyclization to closed product **7a** with LiHMDS, we confirmed by ^1^H NMR analysis that the main diastereoisomer formed (90:10) was the same as that obtained using magnesium or indium. Under identical conditions employing DMF as the solvent, conversion was again complete; however, the main product was closed adduct **7a** (80:20), and two diastereoisomers formed in almost the same amounts (entry 4). The addition of LiCl to the prenylzinc reagent had a profound impact on stereoselectivity, both in THF and in DMF. While the **7a**:**8a** ratio changed only slightly, the diastereoselectivity significantly decreased in THF (entry 5 vs entry 3). Conversely, only one diasteroisomer was obtained using DMF (entry 6 vs entry 4), displaying the opposite configuration of the newly formed stereocenter with respect to the previous cases. Finally, by using water as the solvent in the presence of NH_4_Cl as the additive (entry 7), a good conversion was obtained after 2.5 h at room temperature, favoring the formation of closed product **7a** (70:30), but with a moderate diasteroselectivity (67:33). The observed stereochemical behavior of prenylzinc reagents, favoring the formation of opposite diasteroisomers by using THF or DMF/LiCl, was fully consistent with the results obtained by Guo in the allylation of chiral aromatic imines.^18a^

The formation of two different diastereoisomers can be easily determined and quantified by ^1^H NMR analysis of crude reaction mixtures, examining the chemical shifts of the hydrogens at positions 2 and 5 of the pyrrolidine ring as well as the chemical shifts of the methyl groups in the specific case of the prenylation reaction (Figure 2).

**Figure 2:**
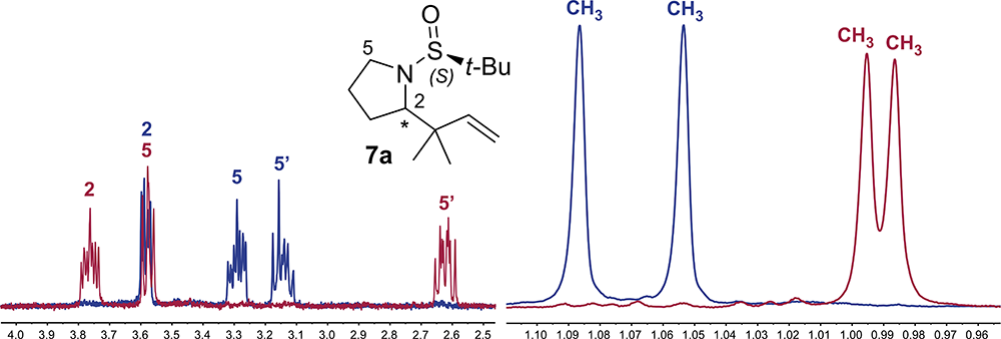
^1^H NMR insets of the crude reaction mixture for the prenylation of imine (*S*)-**4a** with indium (blue) and zinc (red).

The stereochemistry of the newly formed stereocenter (C2) can be deduced by considering that prenylmagnesium, prenylindium, and prenylzinc in THF react by adopting a closed six-membered chair transition state, while an open antiperiplanar transition state is preferred using strongly coordinating solvents (DMF) in the presence of LiCl (Figure 3).

**Figure 3:**
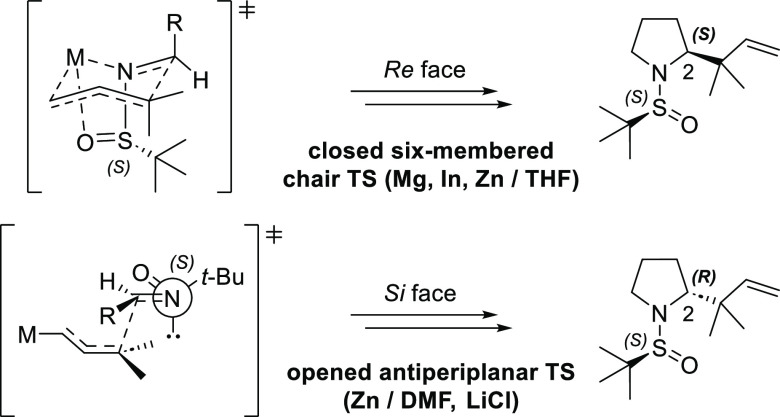
Closed and open transition states involved in the prenylation of imine (*S*)-**4a**.

Once we had established the use of indium in THF at 60 °C (protocol A) and the use of zinc/LiCl in DMF (protocol B), followed by cyclization of the crude reaction mixture with LiHMDS in THF, as the two most efficient stereodivergent methods for the synthesis of **7a** (Table 2, entries 1–3), we extended the allylation reaction to include additional disubstituted cyclic allyl bromides (**6b**–**d**). The reaction with prenylzinc bromide was scaled up to 5 mmol using enantiomeric imine (*R*)-**4a**, with very good results (entry 3). The NMR spectra of the resultant product were completely superimposable with those derived from the reaction of prenylindium bromide with imine (*S*)-**4a** (entry 1), and we further confirmed the two products to be enantiomers by comparing the sign of their optical rotations.

**Table 2:**
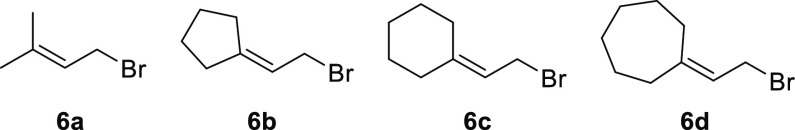
Synthesis of *N*-Sulfinyl 2-Allyl Pyrrolidines **7**^a^

entry	**6**	protocol	**7** [yield^b^ (%)]	**7** dr^c^
1	**6a**	A	**7a** (92)	>99:1
2	**6a**	B	**7a** (82)	>99:1
3	**6a**	B^d^	**7a** (71)	>99:1
4	**6b**	A	**7b** (87)	>99:1
5	**6b**	B	**7b** (85)	>99:1
6	**6c**	A	**7c** (15)	nd
7	**6c**	B	**7c** (69)	>99:1
8	**6d**	A	**7d** (<5)	nd
9	**6d**	B	**7d** (46)	nd
10	**6d**	B^e^	**7d** (74)	>99:1

a Reactions run on 2 mmol of imine (*S*)-**4a** using protocol A (In, THF, 60 °C, 6 h) or protocol B (Zn, LiCl, DMF, 0 °C for 5 h, rt for 12 h), followed by cyclization of the crude reaction mixture (LiHMDS, THF, 0 °C to rt, 1 h).

b Isolated yield after purification by column chromatography.

c Determined by ^1^H NMR analysis of the crude reaction mixtures. nd = not determined.

d Reaction on 5 mmol of imine (*R*)-**4a**.

e At 50 °C.

We were very pleased to find that bromide **6b** afforded the desired product **7b** in very good, isolated yields and with a complete diastereoselectivity using both protocols (entries 4 and 5). However, different results in terms of reactivity were observed with bulkier bromides **6c** and **6d**. When using indium (protocol A), we observed a drastically reduced reactivity for both bromides (entries 6 and 8), a trend that was somewhat attenuated using zinc (protocol B), albeit with diminished product yields of **7c** (69%, entry 7) and **7d** (46%, entry 9), compared to less hindered bromides. Notably, a slight improvement in the yield (74%) was obtained in the case of **7d**, simply by running the reaction under protocol B conditions but increasing the temperature to 50 °C (entry 10).

Once we had obtained the precursors (*S*,*R*)-**7a**–**d**, the reaction sequence to the corresponding (*R*)-homoprolines **11a**–**d** was completed by a three-step sequence of standard synthetic transformations (Scheme 2). All reactions gave satisfactory yields of isolated products, considering the presence of a quite sterically hindered quaternary carbon atom at the α-position of the reaction center, particularly for ozonolysis and Pinnick oxidation. Interestingly, the Pinnick reaction gave **11** in approximately 50% yields, albeit with near-complete conversions, because ∼50% of aldehyde **10** could be recovered alongside product **11** by flash chromatography.

**Scheme 2:**
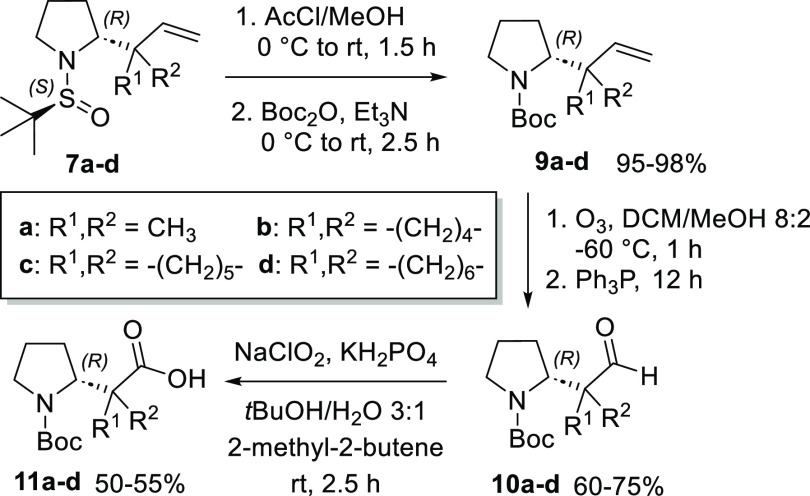
Synthesis of β-Homoprolines **11a**–**d**

In conclusion, we have developed a simple and highly diastereoselective route for the preparation of yet undisclosed chiral α-disubstituted β-homoprolines **11**, which is characterized by a short sequence of operationally simple, cost-effective, and reliable synthetic steps. Furthermore, this procedure allows the introduction of different substituents at the α-position of β-homoproline simply by changing the nature of the starting bromides **6**, opening the possibility for further functionalizations and structural modifications.

## Data Availability

The data underlying this study are available in the published article and its Supporting Information.

## Supporting Information Available

General experimental procedures, analytical data, and spectral data for new compounds (PDF)
