# Supplementary material for: Stereoselective Synthesis of α-Disubstituted β-Homoprolines
Source: Org Lett. 2023 Sep 20;25(38):7067–71. doi: 10.1021/acs.orglett.3c02891 (PMC10546376; doi:10.1021/acs.orglett.3c02891)
Supplement: Supplementary file 1 — ol3c02891_si_001.pdf [file ol3c02891_si_001.pdf]

Supporting Information

Stereoselective Synthesis of α-Disubstituted β-Homoprolines

Arianna Quintavalla, Davide Carboni, Maria Simeone, Marco Lombardo**

Department of Chemistry "G. Ciamician" & Center for Chemical Catalysis - C3, Alma Mater Studiorum, University of Bologna, via P. Gobetti 85, I-40129 Bologna, Italy

Corresponding authors:

Marco Lombardo. E-mail: marco.lombardo@unibo.it

Arianna Quintavalla. E-mail: arianna.quintavalla@unibo.it

Table of Contents

General Information S3

General Methods S3

Preparation of Known Starting Materials S3

Experimental Procedures S4

General Procedure A: Allylation Reaction Using Indium in THF[1] (Protocol A, Table 2) S4

General Procedure B: Allylation Reaction Using Zinc/LiCl in DMF[2] (Protocol B, Table 2) S4

General Procedure C: Removal of the Sulfinyl Group and Boc Protection (Scheme 4, Step 1) S4

General Procedure D: Ozonolysis Reaction[3] (Scheme 4, Step 2) S5

General Procedure E: Pinnick Oxidation[4] (Scheme 4, Step 3) S5

Characterization Data of the Products S6

NMR & HPLC-MS Spectra S12

References S49

General Information

General Methods

All the commercial chemicals were purchased from Sigma-Aldrich, VWR, Alfa Aesar, or TCI-Chemicals and used without additional purifications. The **^{1}H** and **^{13}C NMR** spectra were recorded on a Varian INOVA 400 NMR instrument with a 5 mm probe or on a Bruker Ascend-600 spectrometer. All chemical shifts have been quoted relative to residue solvent signal; chemical shifts (δ) are reported in ppm and coupling constants (*J*) are reported in hertz (Hz). Low-resolution MS (**LRMS**) ESI analyses were performed on an Agilent Technologies MSD1100 single-quadrupole mass spectrometer. Mass spectrometric detection was performed in the full-scan mode from *m/z* 50 to 2500, with a scan time of 0.1 s in the positive ion mode, ESI spray voltage of 4500 V, nitrogen gas pressure of 35 psi, drying gas flow rate of 11.5 mL min^{-1} and fragmentor voltage of 30 V. High-resolution MS (**HRMS**) ESI analyses were performed on a Xevo G2-XS QTof (Waters) mass spectrometer. Mass spectrometric detection was performed in the full-scan mode from *m/z* 50 to 1200, with a scan time of 0.15 s in the positive ion mode, cone voltage: 40 V, collision energy: 6.00 eV. ESI: capillary: 3kV, cone: 40 V, source temperature: 120 °C, desolvation temperature: 600 °C, cone gas flow: 50 L/h, desolvation gas flow: 1000 L/h. **GC-MS** analyses were performed on a Hewlett-Packard 5971 with GC injection, EI ionization at 70 eV. They are reported as: m/z (rel. intensity). **HPLC** analyses were performed on an Agilent Technologies HP1260 instrument. A Phenomenex Gemini C18 3 µm (100 x 3 mm) column was employed for the chromatographic separation: mobile phase H_2O/CH_3CN, gradient from 30% to 80% of CH_3CN in 8 min, 80% of CH_3CN until 22 min, then up to 90% of CH_3CN in 2 min, flow rate 0.4 mL/min. Melting point (**m.p.**) measurements were performed on Bibby Stuart Scientific SMP3 apparatus. Optical rotation measurements (**$[\alpha]_D^{20}$**) were performed on a polarimeter Schmidt+Haensch UniPol L1000. **Flash chromatography** purifications were carried out using VWR silica gel (40 – 63 µm particle size). **Thin-layer chromatography** was performed on Merck 60 F254 plates.

Preparation of Known Starting Materials

Prenyl bromide **6a** is a commercial reagent, while bromides **6b-d**[5] and sulfinimines (*S*)-**4a** and (*R*)-**4a**[6] were prepared according to known literature procedures and were stored under nitrogen at -30 °C. Their spectroscopical data matched the reported ones.

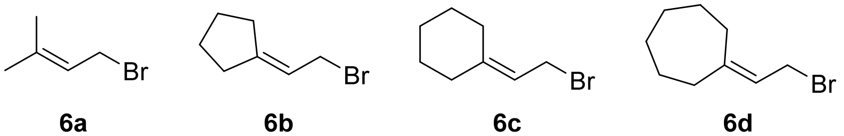

6a **6b** **6c** **6d**

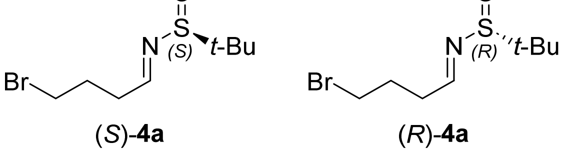

(*S*)-**4a** (*R*)-**4a**

Prenylmagensium bromide was prepared according to a reported literature procedure.[7]

Experimental Procedures

General Procedure A: Allylation Reaction Using Indium in THF[1] (Protocol A, Table 2)

In a screw cap septum vial equipped with a magnetic stir bar, indium powder (0.34 g, 3 mmol) is added under nitrogen to a solution of imine **4a** (0.51 g, 2 mmol) and bromide **6** (3 mmol, 1.5 equiv.) in dry THF (4 mL). The vial is sealed, immersed in a pre-heated oil bath at 60 °C and vigorously stirred at 60 °C for 6 h, during which time the solution color gradually turned orange. After completion, monitored by TLC, the solution is cooled (0 °C) and the reaction is quenched with saturated aqueous NH_4Cl solution. The mixture is extracted with EtOAc (3x), the combined organic phases are dried over anhydrous Na_2SO_4 and concentrated under reduced pressure. The crude reaction mixture is dissolved in dry THF (4 mL) under nitrogen. LiHMDS (1.0 M solution in THF, 3 mL, 3 mmol) is added at 0 °C to the solution, and the reaction is stirred for 1 h at room temperature. The reaction mixture is quenched with saturated aqueous NH_4Cl solution and extracted with EtOAc (3x). The combined organic phases are dried over anhydrous Na_2SO_4 and concentrated under reduced pressure. Product **7** is purified by flash-column chromatography on silica, eluting with cyclohexane:EtOAc mixtures (gradient elution from 100:0 to 80:20).

General Procedure B: Allylation Reaction Using Zinc/LiCl in DMF[2] (Protocol B, Table 2)

Zinc powder (0.2 g, 3 mmol) is added at 0 °C under nitrogen to a solution of imine **4a** (0.51 g, 2 mmol), flamed-dry LiCl (0.13g, 3 mmol) and bromide **6** (3 mmol, 1.5 equiv.) in dry DMF (4 mL) and the reaction is vigorously stirred at 0 °C for 5 h. The cooling bath is removed, and the reaction is further stirred at room temperature for 12 h. The solution is cooled (0 °C) and the reaction is quenched with saturated aqueous NH_4Cl solution. The mixture is extracted with EtOAc (3x), the combined organic phases are dried over anhydrous Na_2SO_4 and concentrated under reduced pressure. The **7**:**8** ratio is checked by 1H-NMR and the crude reaction mixture is dissolved in dry THF (4 mL) under nitrogen. LiHMDS (1.0 M solution in THF, 1.5 equivalents with respect to the amount of **8**) is added at 0 °C to the solution, and the reaction is stirred for 1 h at room temperature. The reaction mixture is quenched with saturated aqueous NH_4Cl solution and extracted with EtOAc (3x). The combined organic phases are dried over anhydrous Na_2SO_4 and concentrated under reduced pressure. Product **7** is purified by flash-column chromatography on silica, eluting with cyclohexane:EtOAc mixtures (gradient elution from 100:0 to 80:20).

General Procedure C: Removal of the Sulfinyl Group and Boc Protection (Scheme 4, Step 1)

Freshly distilled acetyl chloride (0.54 mL, 7.5 mmol) is added at 0 °C to a solution of **7** (1.5 mmol, 1.0 equiv.) in MeOH (5 mL), and the reaction is stirred for 1 h, while allowed to reach room temperature. After completion, monitored by TLC, the reaction mixture is concentrated under reduced pressure and the crude chlorohydrate salt is dissolved in CH_2Cl_2 (4 mL) and cooled to 0 °C. Boc_2O (0.42 mL, 1.8 mmol) and Et_3N (0.5 mL, 1.8 mmol) are added and the reaction is stirred for

2.5 h, while allowed to reach room temperature. After completion, monitored by TLC, the reaction mixture is quenched with saturated aqueous NH_4Cl solution and extracted with EtOAc (3x). The combined organic phases are washed with 1M HCl (3x), brine, dried over anhydrous Na_2SO_4 and concentrated under reduced pressure. Product **9** is purified by flash-column chromatography on silica, eluting with cyclohexane:EtOAc mixtures (gradient elution from 100:0 to 90:10).

General Procedure D: Ozonolysis Reaction[3] (Scheme 4, Step 2)

Ozone is bubbled through a solution of **9** (1 mmol, 1.0 equiv.) in CH_2Cl_2/methanol 4:1 (4 mL) at −60 °C for 1 h, until the characteristic blue color of ozone persists in the solution. Oxygen is then bubbled for 5 min, until the blue color disappears, and the reaction is quenched by adding Ph_3P (0.32 g, 1.2 mmol). The reaction mixture is stirred at room temperature for 12 h and concentrated under reduced pressure. Product **10** is obtained after purification of the residue by flash-column chromatography on silica, eluting with cyclohexane:EtOAc mixtures (gradient elution from 100:0 to 85:15). **Caution!** Ozone is a potent oxidant and a biocide. The ozonolysis reactions must be carried out within a fume hood and exit gases must be quenched with a suitable reducing agent (aq. $Na_2S_2O_3$). Methanol is added as a co-solvent to steer the reaction towards the formation of easily reduced and less dangerous hydroperoxyacetals.

General Procedure E: Pinnick Oxidation[4] (Scheme 4, Step 3)

A freshly prepared solution of $NaClO_2$ (80% w/w, 0.23 g, 2 mmol) and KH_2PO_4 (0.27 g, 2 mmol) in H_2O (2 mL) is added to a solution of **10** (1 mmol, 1.0 equiv.) in *t*BuOH/2-methyl-2-butene 2:1 (9 mL) and the reaction mixture is vigorously stirred at room temperature for 2.5 h. The reaction is extracted with AcOEt (3x), the combined organic phases are dried over anhydrous Na_2SO_4 and concentrated under reduced pressure. The residue is purified by flash-column chromatography on silica, eluting with cyclohexane:EtOAc mixtures, starting from 75:25 to recover the unreacted aldehyde **10**, and finishing with 50:50 to isolate the product **11**.

Characterization Data of the Products

(*S*)-1-((*S*)-*tert*-butylsulfinyl)-2-(2-methylbut-3-en-2-yl)pyrrolidine (*S*,*S*-7a)

Obtained as a colorless oil in 92% isolated yield (0.45 g, 1.85 mmol), starting from (*S*)-**4a** (0.508 g, 2 mmol) and bromide **6a** (0.447 g, 3 mmol), following general procedure A (Table 1, entry 1).

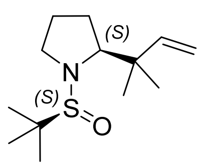

Y: 92%. $[\alpha]_D^{20} = -71°$ (c = 1.61 in $CHCl_3$). **^{1}H NMR** (400 MHz, $CDCl_3$) δ 5.98 – 5.82 (m, 1H), 5.04 – 4.99 (m, 1H), 4.97 (s, 1H), 3.58 (dd, *J* = 8.0, 3.7 Hz, 1H), 3.29 (ddd, *J* = 11.1, 7.6, 3.6 Hz, 1H), 3.14 (dt, *J* = 11.1, 7.2 Hz, 1H), 1.93 – 1.66 (m, 4H), 1.23 (s, 9H), 1.09 (s, 3H), 1.05 (s, 3H). **^{13}C NMR** (150 MHz, $CDCl_3$) δ 145.7, 112.0, 67.6, 58.7, 48.5, 41.9, 27.3, 25.0, 24.9, 24.6, 23.7. **LRMS** (ESI) *m/z* = 244 $[M + H]^+$, 266 $[M + Na]^+$, 266 $[M + Na]^+$, 307 $[M + Na + CH_3CN]^+$, 509 $[2M + Na]^+$, 768 $[3M+K]^+$. **HRMS** *m*/z: $[M + H]^+$ Calcd. for $[C_{13}H_{26}NOS]^+$ 244.1735; Found. 244.1738

(*R*)-1-((*S*)-*tert*-butylsulfinyl)-2-(2-methylbut-3-en-2-yl)pyrrolidine (*R*,*S*-7a)

Obtained as a colorless oil in 82% isolated yield (0.4 g, 1.64 mmol), starting from (*S*)-**4a** (0.508 g, 2 mmol) and bromide **6a** (0.447 g, 3 mmol), following general procedure B (Table 1, entry 2).

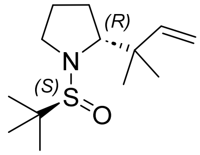

$[\alpha]_D^{20} = +96$ ° (c = 1.07 in $CHCl_3$). **^{1}H NMR** (600 MHz, $CDCl_3$) δ 5.83 (dd, *J* = 17.5, 10.9 Hz, 1H), 5.00 (dd, *J* = 5.8, 1.4 Hz, 1H), 4.96 (dd, *J* = 13.8, 1.4 Hz, 1H), 3.84 – 3.73 (m, 1H), 3.59 (dd, *J* = 7.9, 6.4 Hz, 1H), 2.64 (ddd, *J* = 10.7, 8.7, 6.3 Hz, 1H), 1.96 – 1.82 (m, 1H), 1.81 – 1.72 (m, 1H), 1.70 – 1.55 (m, 2H), 1.26 (s, 9H), 1.01 (s, 3H), 1.00 (s, 3H). **^{13}C NMR** (150 MHz, $CDCl_3$) δ 145.4, 112.3, 75.0, 58.9, 58.9, 44.3, 41.8, 27.8, 27.7, 24.9, 24.7, 23.9. **LRMS** (ESI) *m/z* = 244 $[M + H]^+$, 266 $[M + Na]^+$, 307 $[M + Na + CH_3CN]^+$, 509 $[2M + Na]^+$, 768 $[3M+K]^+$. **HRMS** *m*/z: $[M + H]^+$ Calcd. for $[C_{13}H_{26}NOS]^+$ 244.1735; Found. 244.1722

(*S*)-1-((*S*)-*tert*-butylsulfinyl)-2-(1-vinylcyclopentyl)pyrrolidine (*S*,*S*-7b)

Obtained as a colorless oil in 87% isolated yield (0.468 g, 1.74 mmol), starting from (*S*)-**4a** (0.508 g, 2 mmol) and bromide **6b** (0.525 g, 3 mmol), following general procedure A (Table 1, entry 4).

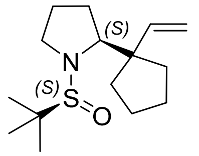

$[\alpha]_D^{20} = -82°$ (c = 0.81 in $CHCl_3$). **^{1}H NMR** (600 MHz, $CDCl_3$) δ 5.90 (ddd, *J* = 17.6, 10.9, 0.8 Hz, 1H), 5.09 (dd, *J* = 10.8, 1.5 Hz, 1H), 5.03 (dd, *J* = 17.6, 1.5 Hz, 1H), 3.62 (dd, *J* = 7.8, 4.6 Hz, 1H), 3.24 (dt, *J* = 10.9, 7.3 Hz, 1H), 3.15 (ddd, *J* = 10.9, 7.6, 4.9 Hz, 1H), 1.96 (ddt, *J* = 9.6, 4.9, 2.0 Hz, 1H), 1.91 – 1.77 (m, 2H), 1.75 – 1.49 (m, 8H), 1.38 (dt, *J* = 12.9, 8.7 Hz, 1H), 1.21 (d, *J* = 1.0 Hz, 9H). **^{13}C NMR** (150 MHz, $CDCl_3$) δ 142.2, 113.9, 69.4, 58.6, 54.7, 47.3, 37.3, 33.9, 28.9, 24.8, 23.9, 23.7, 22.9. **LRMS** (ESI) *m/z* = 270 $[M + H]^+$, 292 $[M + Na]^+$, 333 $[M + Na + CH_3CN]^+$, 561 $[2M + Na]^+$. **HRMS** *m*/z: $[M + H]^+$ Calcd. for $[C_{15}H_{28}NOS]^+$ 270.1892; Found. 270.1877

(*R*)-1-((*S*)-*tert*-butylsulfinyl)-2-(1-vinylcyclopentyl)pyrrolidine (*R*,*S*-7b)

Obtained as a colorless oil in 85% isolated yield (0.46 g, 1.71 mmol), starting from (*S*)-**4a** (0.508 g, 2 mmol) and bromide **6b** (0.525 g, 3 mmol), following general procedure B (Table 1, entry 5).

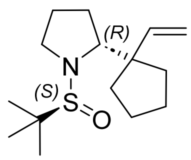

$[\alpha]_D^{20}$ = +117 ° (c = 1.33 in $CHCl_3$). **^{1}H NMR** (600 MHz, $CDCl_3$) δ (ddd, *J* = 17.6, 10.9, 0.7 Hz, 1H), 5.09 (dd, *J* = 10.9, 1.5 Hz, 1H), 5.00 (dd, *J* = 17.6, 1.5 Hz, 1H), 3.83 – 3.73 (m, 1H), 3.71 – 3.61 (m, 1H), 2.64 (ddd, *J* = 10.6, 8.7, 6.4 Hz, 1H), 1.97 – 1.83 (m, 1H), 1.83 – 1.73 (m, 1H), 1.72 – 1.51 (m, 9H), 1.37 (ddd, *J* = 12.8, 9.4, 6.0 Hz, 1H), 1.25 (s, 9H). **^{13}C NMR** (150 MHz, $CDCl_3$) δ 142.0, 113.7, 74.9, 58.5, 54.8, 44.1, 36.3, 33.5, 28.8, 27.5, 24.7, 23.6, 23.2. **LRMS** (ESI) *m/z* = 270 $[M + H]^+$, 292 $[M + Na]^+$, 333 $[M + Na + CH_3CN]^+$, 561 $[2M + Na]^+$. **HRMS** *m*/z: $[M + H]^+$ Calcd. for $[C_{15}H_{28}NOS]^+$ 270.1892; Found. 270.1890

(*R*)-1-((*S*)-*tert*-butylsulfinyl)-2-(1-vinylcyclohexyl)pyrrolidine (*R*,*S*-7c)

Obtained as a colorless oil in 69% isolated yield (0.39 g, 1.38 mmol), starting from (*S*)-**4a** (0.508 g, 2 mmol) and bromide **6c** (0.567 g, 3 mmol), following general procedure B (Table 1, entry 7).

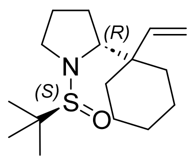

$[\alpha]_D^{20}$ = +96 ° (c = 1.55 in $CHCl_3$). **^{1}H NMR** (400 MHz, $CDCl_3$) δ 5.62 (dd, *J* = 17.9, 11.0 Hz, 1H), 5.20 (dd, *J* = 11.2, 1.4 Hz, 1H), 5.01 (dd, *J* = 17.8, 1.5 Hz, 1H), 3.75 (ddd, *J* = 10.7, 7.3, 3.5 Hz, 1H), 3.65 (t, *J* = 7.0 Hz, 1H), 2.64 – 2.52 (m, 1H), 1.87 – 1.77 (m, 1H), 1.77 – 1.68 (m, 2H), 1.69 – 1.30 (m, 9H), 1.28 (s, 9H), 1.27 – 1.16 (m, 2H). **^{13}C NMR** (150 MHz, $CDCl_3$) δ 142.5, 115.7, 74.9, 58.9, 44.6, 44.4, 33.5, 32.8, 27.8, 26.8, 26.2, 24.9, 21.9, 21.8. **LRMS** (ESI) *m/z* = 284 $[M + H]^+$, 306 $[M + Na]^+$, 347 $[M + Na + CH_3CN]^+$, 589 $[2M + Na]^+$. **HRMS** *m*/z: $[M + H]^+$ Calcd. for $[C_{16}H_{30}NOS]^+$ 284.2048; Found. 284.2064

(*R*)-1-((*S*)-*tert*-butylsulfinyl)-2-(1-vinylcycloheptyl)pyrrolidine (*R*,*S*-7d)

Obtained as a colorless oil in 74% isolated yield (0.44 g, 1.48 mmol), starting from (*S*)-**4a** (0.508 g, 2 mmol) and bromide **6d** (0.609 g, 3 mmol), following general procedure B at 50 °C (Table 1, entry 10).

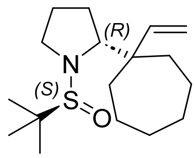

$[\alpha]_D^{20}$ = +84 ° (c = 1.0 in $CHCl_3$). **^{1}H NMR** (600 MHz, $CDCl_3$) δ 5.68 (dd, *J* = 17.8, 11.0 Hz, 1H), 5.07 (dd, *J* = 11.0, 1.5 Hz, 1H), 4.97 (dd, *J* = 17.8, 1.5 Hz, 1H), 3.80 – 3.64 (m, 2H), 2.58 (ddd, *J* = 10.6, 8.4, 6.4 Hz, 1H), 1.92 – 1.77 (m, 2H), 1.77 – 1.69 (m, 2H), 1.67 – 1.50 (m, 6H), 1.50 – 1.34 (m, 6H), 1.26 (s, 9H). **^{13}C NMR** (150 MHz, $CDCl_3$) δ 143.7, 113.3, 74.2, 59.0, 47.8, 44.4, 35.3, 34.2, 30.3, 30.1, 27.8, 27.4, 24.9, 22.5, 22.0. **LRMS** (ESI) *m/z* = 298 $[M + H]^+$, 320 $[M + Na]^+$, 361 $[M + Na + CH_3CN]^+$, 617 $[2M + Na]^+$. **HRMS** *m*/z: $[M + H]^+$ Calcd. for $[C_{17}H_{32}NOS]^+$ 298.2205; Found. 298.2198

***tert*-butyl (*R*)-2-(2-methylbut-3-en-2-yl)pyrrolidine-1-carboxylate (*R*-9a)**

Obtained as a colorless oil in 98% isolated yield (0.35 g, 1.47 mmol), starting from (*R*,*S*)-**7a** (0.365 g, 1.5 mmol) and following general procedure C.

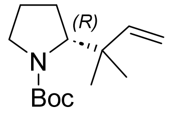

$[\alpha]_D^{20}$ = +40 ° (c = 1.7 in $CHCl_3$). **^{1}H NMR** (400 MHz, $CDCl_3$) δ 5.82 (dd, *J* = 17.8, 10.5 Hz, 1H), 4.99 – 4.96 (m, 1H), 4.96 – 4.91 (m, 1H), 3.85 (broad s, 1H), 3.66 (broad s, 1H), 3.13 (ddd, *J* = 11.2, 7.7, 5.5 Hz, 1H), 1.86 – 1.63 (m, 4H), 1.46 (s,

9H), 0.99 (s, 6H). **^{13}C NMR** (100 MHz, $CDCl_3$) δ 155.1, 145.6, 111.8, 79.2, 64.8, 47.7 (broad), 42.7, 28.4, 26.9 (broad), 24.8, 24.4, 22.0. **LRMS** (ESI) *m/z* = 184 [M − CH_2=C(CH_3)$_2$ + H]$^+$, 240 [M + H]$^+$, 262 [M + Na]$^+$, 303 [M + Na + CH_3CN]$^+$, 501 [2M + Na]$^+$. **HRMS** *m*/z: [M + H]$^+$ Calcd. for [$C_{14}H_{26}NO_2$]$^+$ 240.1964; Found. 240.1966

***tert*-butyl (*R*)-2-(1-vinylcyclopentyl)pyrrolidine-1-carboxylate (*R*-9b)**

Obtained as a colorless oil in 95% isolated yield (0.38 g, 1.9 mmol), starting from (*R*,*S*)-**7b** (0.27 g, 1.5 mmol) and following general procedure C.

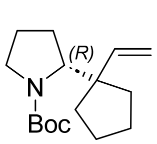

$[\alpha]_D^{20}$ = +76 ° (c = 1.0 in $CHCl_3$). **^{1}H NMR** (600 MHz, $CDCl_3$) δ 5.74 (dd, *J* = 17.6, 10.8 Hz, 1H), 5.06 (d, *J* = 10.9 Hz, 1H), 5.00 (d, *J* = 17.5 Hz, 1H), 3.93 (broad s, 1H), 3.60 (broad s, 1H), 3.15 (dtd, *J* = 11.6, 5.6, 2.8 Hz, 1H), 1.88 – 1.78 (m, 2H), 1.76 – 1.49 (m, 8H), 1.46 (s, 9H), 1.45 – 1.37 (m, 2H). **^{13}C NMR** (150 MHz, $CDCl_3$) δ 156.0, 142.6, 113.6, 79.1 (broad), 64.2, 55.7, 47.9, 34.6, 33.6, 28.5, 23.0, 22.6. **LRMS** (ESI) *m/z* = 210 [M − CH_2=C(CH_3)$_2$ + H]$^+$, 266 [M + H]$^+$, 288 [M + Na]$^+$, 329 [M + Na + CH_3CN]$^+$, 553 [2M + Na]$^+$. **HRMS** *m*/z: [M + H]$^+$ Calcd. for [$C_{16}H_{28}NO_2$]$^+$ 266.2120; Found. 266.2102

***tert*-butyl (*R*)-2-(1-vinylcyclohexyl)pyrrolidine-1-carboxylate (*R*-9c)**

Obtained as a colorless oil in 98% isolated yield (0.41 g, 1.47 mmol), starting from (*R*,*S*)-**7c** (0.425 g, 1.5 mmol) and following general procedure C.

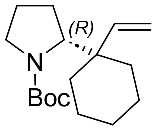

11c

$[\alpha]_D^{20}$ = +60 ° (c = 1.53 in $CHCl_3$). **^{1}H NMR** (400 MHz, $CDCl_3$) δ 5.57 (dd, *J* = 17.9, 11.0 Hz, 1H), 5.20 (d, *J* = 11.0 Hz, 1H), 5.03 (d, *J* = 17.9 Hz, 1H), 3.83 (broad s, 1H), 3.65 (broad s, 1H), 3.14 – 3.02 (m, 1H), 1.86 – 1.73 (m, 3H), 1.74 – 1.61 (m, 4H), 1.47 (s, 9H), 1.43 – 1.22 (m, 6H), 1.22 – 1.09 (m, 1H). **^{13}C NMR** (150 MHz, $CDCl_3$) δ 156.4, 142.6, 116.1, 78.9 (broad), 66.1, 47.8, 45.9, 33.1, 28.5, 26.4, 22.1, 22.0. **LRMS** (ESI) *m/z* = 224 [M − CH_2=C(CH_3)$_2$ + H]$^+$, 280 [M + H]$^+$, 302 [M + Na]$^+$, 343 [M + Na + CH_3CN]$^+$, 581 [2M + Na]$^+$. **HRMS** *m*/z: [M + H]$^+$ Calcd. for [$C_{17}H_{30}NO_2$]$^+$ 280.2277; Found. 280.2283

***tert*-butyl (*R*)-2-(1-vinylcycloheptyl)pyrrolidine-1-carboxylate (*R*-9d)**

Obtained as a colorless oil in 98% isolated yield (0.43 g, 1.47 mmol), starting from (*R*,*S*)-**7d** (0.446 g, 1.5 mmol) and following general procedure C.

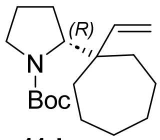

11d

$[\alpha]_D^{20}$ = +52 ° (c = 1.6 in $CHCl_3$). **^{1}H NMR** (400 MHz, $CDCl_3$) δ 5.64 (dd, *J* = 17.7, 11.0 Hz, 1H), 5.03 (dd, *J* = 11.0, 1.5 Hz, 1H), 4.96 (dd, *J* = 17.7, 1.5 Hz, 1H), 3.96 (broad s, 1H), 3.66 (broad s, 1H), 3.07 (ddd, *J* = 11.4, 8.0, 6.0 Hz, 1H), 1.86 – 1.69 (m, 5H), 1.68 – 1.49 (m, 6H), 1.47 (s, 9H), 1.46 – 1.35 (m, 5H). **^{13}C NMR** (150 MHz, $CDCl_3$) δ 156.4, 144.5, 112.9, 79.2 (broad), 64.7, 48.3 (broad), 34.9, 34.2, 30.8, 27.4, 26.4 (broad), 24.3 (broad), 22.5. **LRMS** (ESI) *m/z* = 238 [M − CH_2=C(CH_3)$_2$ + H]$^+$, 294 [M + H]$^+$, 316 [M + Na]$^+$, 357 [M + Na + CH_3CN]$^+$, 609 [2M + Na]$^+$. **HRMS** *m*/z: [M + H]$^+$ Calcd. for [$C_{14}H_{25}NO_2$]$^+$ 294.2433; Found. 294.2420

***tert*-butyl (*R*)-2-(2-methyl-1-oxopropan-2-yl)pyrrolidine-1-carboxylate (*R*-10a)**

Obtained as a colorless oil in 75% isolated yield (0.18 g, 0.75 mmol), starting from (*R*)-**9a** (0.24 g, 1 mmol) and following general procedure D.

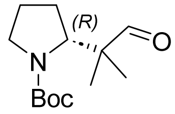

$[\alpha]_D^{20}$ = +16 ° (c = 1.0 in $CHCl_3$). **^{1}H NMR** (400 MHz, $CDCl_3$) δ 9.55 (broad s, 1H), 4.10 (broad s, 1H), 3.81 – 3.53 (broad m, 1H), 3.15 (td, *J* = 11.0, 6.8 Hz, 2H), 1.99 (broad s, 2H), 1.89 – 1.70 (m, 4H), 1.44 (s, 9H), 1.01 (broad s, 3H), 0.98 (broad s, 9H). **^{13}C NMR** (100 MHz, $CDCl_3$) δ 203.9 (broad), 155.7 (broad), 79.5 (broad), 61.2, 50.4, 47.7, 28.3, 24.2 (broad), 20.0 (broad), 15.6 (broad). **LRMS** (ESI) *m/z* = 186 [M − CH_2=$C(CH_3)_2$ + H]$^+$, 264 [M + Na]$^+$, 305 [M + Na + CH_3CN]$^+$, 505 [2M + Na]$^+$. **HRMS** *m*/z: [M + Na]$^+$ Calcd. for $[C_{13}H_{23}NNaO_3]^+$ 264.1576; Found. 264.1597

***tert*-butyl (*R*)-2-(1-formylcyclopentyl)pyrrolidine-1-carboxylate (*R*-10b)**

Obtained as a colorless oil in 63% isolated yield (0.168 g, 0.63 mmol), starting from (*R*)-**9b** (0.265 g, 1 mmol) and following general procedure D.

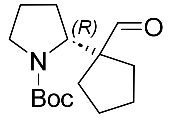

$[\alpha]_D^{20}$ = +72 ° (c = 1.12 in $CHCl_3$). **^{1}H NMR** (600 MHz, $CDCl_3$) δ 9.55 (broad s, 1H), 4.24 (broad s, 1H), 3.77 – 3.52 (broad m, 1H), 3.14 (dt, *J* = 11.2, 7.2 Hz, 1H), 2.12 – 1.98 (m, 2H), 1.88 – 1.80 (m, 1H), 1.69 – 1.60 (m, 4H), 1.69 – 1.60 (m, 1H), 1.61 – 1.55 (m, 3H), 1.53 – 1.47 (m, 1H), 1.44 (s, 9H). **^{13}C NMR** (150 MHz, $CDCl_3$) δ 203.3, 155.5, 79.7 (broad), 60.6, 47.8, 28.3, 27.7 (broad), 25.2 (broad), 24.7 (broad). **LRMS** (ESI) *m/z* = 212 [M−*t*Bu+H]$^+$, 290 [M + Na]$^+$, 331 [M + Na + CH_3CN]$^+$, 557 [2M + Na]$^+$. **HRMS** *m*/z: [M + Na]$^+$ Calcd. for $[C_{15}H_{25}NNaO_3]^+$ 290.1732; Found. 290.1718

***tert*-butyl (*R*)-2-(1-formylcyclohexyl)pyrrolidine-1-carboxylate (*R*-10c)**

Obtained as a colorless oil in 60% isolated yield (0.17 g, 0.6 mmol), starting from (*R*)-**9c** (0.28 g, 1 mmol) and following general procedure D.

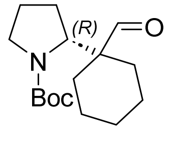

$[\alpha]_D^{20}$ = +58 ° (c = 2.2 in $CHCl_3$). **^{1}H NMR** (600 MHz, $CDCl_3$) δ 9.54 (broad s, 1H), 4.00 (broad s, 1H), 3.81 – 3.48 (broad m, 2H), 3.21 – 2.97 (m, 2H), 2.04 – 1.96 (m, 2H), 1.94 – 1.81 (m, 2H), 1.80 – 1.72 (m, 2H), 1.68 – 1.56 (m, 4H), 1.45 (s, 9H), 1.31 – 1.17 (m, 2H), 1.17 – 1.03 (m, 2H). **^{13}C NMR** (150 MHz, $CDCl_3$) δ 206.4 (broad), 155.9 (broad), 79.6 (broad), 62.4 (broad), 55.1 (broad), 47.5, 28.4, 27.0, 25.7, 22.8, 22.3. **LRMS** (ESI) *m/z* = 226 [M−*t*Bu+H]$^+$, 304 [M + Na]$^+$, 345 [M + Na + CH_3CN]$^+$, 585 [2M + Na]$^+$. **HRMS** *m*/z: [M + Na]$^+$ Calcd. for $[C_{16}H_{27}NNaO_3]^+$ 304.1889; Found. 290.1875

***tert*-butyl (*R*)-2-(1-formylcycloheptyl)pyrrolidine-1-carboxylate (*R*-10d)**

Obtained as a white solid in 72% isolated yield (0.212 g, 0.72 mmol), starting from (*R*)-**9d** (0.29 g, 1 mmol) and following general procedure D.

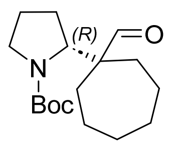

m.p. = 40-41 °C. $[\alpha]_D^{20}$ = +38 ° (c = 1.0 in $CHCl_3$). **^{1}H NMR** (600 MHz, $CDCl_3$) δ 9.48 (broad s, 1H), 4.08 (broad s, 1H), 3.83 – 3.52 (broad m, 1H), 3.13 (dt, *J* = 11.3, 7.2 Hz, 1H), 2.06 (dd, *J* = 13.2, 9.2 Hz, 1H), 1.96 (dq, *J* = 16.3, 8.3 Hz, 1H), 1.85 (dd, *J* = 14.7, 8.9 Hz, 2H), 1.82 – 1.73 (m, 2H), 1.68 (dd, *J* = 14.8, 10.4 Hz, 1H), 1.65 – 1.58 (m, 2H), 1.53 – 1.48 (m, 6H), 1.45 (s, 9H), 1.35 – 1.25 (m, 1H). **^{13}C NMR** (150 MHz, $CDCl_3$) δ 205.1, 156.0 (broad), 79.7 (broad), 62.7, 57.5, 47.9 (broad), 30.7 (broad), 30.2, 30.1, 28.5, 28.3, 23.9 (broad), 23.4. **LRMS** (ESI) *m/z* = 240 $[M{-}tBu{+}H]^+$, 318 $[M + Na]^+$, 359 $[M + Na + CH_3CN]^+$, 613 $[2M + Na]^+$. **HRMS** *m*/z: $[M + Na]^+$ Calcd. for $[C_{17}H_{29}NNaO_3]^+$ 318.2045; Found. 318.2022

(*R*)-2-(1-(*tert*-butoxycarbonyl)pyrrolidin-2-yl)-2-methylpropanoic acid (*R*-11a)

Obtained as a colorless oil in 55% isolated yield (0.14 g, 0.55 mmol), starting from (*R*)-**10a** (0.24 g, 1 mmol) and following general procedure E.

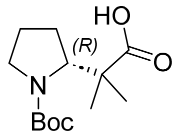

$[\alpha]_D^{20}$ = +80 ° (c = 1.0 in $CHCl_3$). **^{1}H NMR** (600 MHz, $CDCl_3$) δ 4.29 (broad d, *J* = 8.0 Hz, 1H), 3.68 (broad s, 1H), 3.26 – 3.16 (m, 1H), 2.07 – 1.95 (m, 2H), 1.88 – 1.80 (m, 2H), 1.80 – 1.72 (m, 4H), 1.44 (broad s, 9H), 1.21 (broad s, 3H), 1.13 (broad s, 3H). **^{13}C NMR** (150 MHz, $CDCl_3$) δ 182.9, 155.9, 79.7 (broad), 62.6, 47.9, 47.7, 28.4, 27.8 (broad), 24.0 (broad), 20.9 (broad). **LRMS** (ESI) *m/z* = 184 $[M{-}tBu{+}H]^+$, 202 $[M + H]^+$, 280 $[M + Na]^+$, 321 $[M + Na + CH_3CN]^+$, 537 $[2M + Na]^+$, 810 $[3M + K]^+$. **HRMS** *m*/z: $[M + H]^+$ Calcd. for $[C_{13}H_{24}NO_4]^+$ 258.1705; Found. 258.1734

(*R*)-2-(1-(*tert*-butoxycarbonyl)pyrrolidin-2-yl)cyclopentane-1-carboxylic acid (*R*-11b)

Obtained as a colorless oil in 52% isolated yield (0.147 g, 0.52 mmol), starting from (*R*)-**10b** (0.267 g, 1 mmol) and following general procedure E.

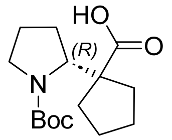

$[\alpha]_D^{20}$ = +11 ° (c = 1.0 in $CHCl_3$). **^{1}H NMR** (600 MHz, $CDCl_3$) δ 4.38 (dd, *J* = 8.5, 2.6 Hz, 1H), 3.66 – 3.56 (m, 1H), 3.23 (ddd, *J* = 10.9, 7.7, 5.3 Hz, 1H), 2.14 – 2.07 (m, 2H), 2.02 – 1.94 (m, 1H), 1.89 – 1.78 (m, 2H), 1.78 – 1.67 (m, 2H), 1.67 – 1.49 (m, 6H), 1.46 (s, 9H). **^{13}C NMR** (150 MHz, $CDCl_3$) δ 182.2, 156.0, 79.7, 61.5, 60.5, 48.0, 34.4, 31.5, 28.9, 28.4, 28.2, 24.2, 23.7. **LRMS** (ESI) *m/z* = 228 $[M{-}tBu{+}H]^+$, 284 $[M + H]^+$, 306 $[M + Na]^+$, 322 $[M + K]^+$, 589 $[2M + Na]^+$. **HRMS** *m*/z: $[M + H]^+$ Calcd. for $[C_{15}H_{26}NO_2]^+$ 284.1862; Found. 284.1841

(*R*)-2-(1-(*tert*-butoxycarbonyl)pyrrolidin-2-yl)cyclohexane-1-carboxylic acid (*R*-11c)

Obtained as a colorless oil in 50% isolated yield (0.149 g, 0.5 mmol), starting from (*R*)-**10c** (0.28 g, 1 mmol) and following general procedure E.

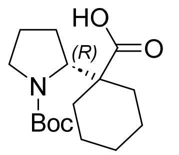

$[\alpha]_D^{20}$ = +35 ° (c = 1.7 in $CHCl_3$). **^{1}H NMR** (600 MHz, $CDCl_3$) δ 4.10 (d, *J* = 8.3 Hz, 1H), 3.70 – 3.55 (m, 1H), 3.17 (ddt, *J* = 10.2, 7.1, 5.3 Hz, 1H), 2.10 (dd, *J* = 29.8, 12.4 Hz, 2H), 2.01 – 1.93 (m, 1H), 1.90 – 1.78 (m, 2H), 1.79 – 1.69 (m, 1H), 1.68 – 1.56 (m, 2H), 1.43 (s, 9H), 1.40 – 1.18 (m, 6H). **^{13}C NMR** (150 MHz, $CDCl_3$) δ 182.2, 156.0, 79.7, 61.5, 60.5, 48.0, 34.4, 31.5, 28.9, 28.4, 28.2, 24.2, 23.7. **LRMS** (ESI) *m/z* = 242 $[M-tBu+H]^+$, 298 $[M + H]^+$, 306 $[M + Na]^+$, 336 $[M + K]^+$, 617 $[2M + Na]^+$. **HRMS** *m*/z: $[M + H]^+$ Calcd. for $[C_{16}H_{28}NO_2]^+$ 298.2018; Found. 298.2037

(*R*)-2-(1-(*tert*-butoxycarbonyl)pyrrolidin-2-yl)cycloheptane-1-carboxylic acid (*R*-11d)

Obtained as a colorless oil in 50% isolated yield (0.156 g, 0.5 mmol), starting from (*R*)-**10d** (0.295 g, 1 mmol) and following general procedure E.

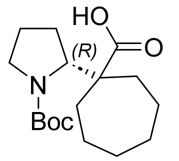

$[\alpha]_D^{20}$ = +37 ° (c = 2.0 in $CHCl_3$). **^{1}H NMR** (600 MHz, $CDCl_3$) δ 4.24 (dd, *J* = 8.4, 3.3 Hz, 1H), 3.74 – 3.60 (m, 1H), 3.21 (dt, *J* = 11.3, 6.9 Hz, 1H), 2.13 (dd, *J* = 14.5, 8.5 Hz, 1H), 2.07 (dd, *J* = 14.3, 8.1 Hz, 1H), 1.98 – 1.85 (m, 2H), 1.82 (dt, *J* = 14.3, 7.1 Hz, 1H), 1.79 – 1.69 (m, 1H), 1.68 – 1.56 (m, 4H), 1.57 – 1.47 (m, 6H), 1.46 (s, 9H). **^{13}C NMR** (150 MHz, $CDCl_3$) δ 181.7, 156.1, 79.7, 64.5, 55.0, 48.0, 32.0 (broad), 29.7, 29.6, 29.5, 28.4, 27.5 (broad), 26.9, 24.0 (broad), 23.8, 23.8.. **LRMS** (ESI) *m/z* = 256 $[M-tBu+H]^+$, 312 $[M + H]^+$, 334 $[M + Na]^+$, 350 $[M + K]^+$, 645 $[2M + Na]^+$. **HRMS** *m*/z: $[M + H]^+$ Calcd. for $[C_{17}H_{30}NO_2]^+$ 312.2175; Found. 312.2203

NMR & HPLC-MS Spectra

(*R*)-1-((*S*)-*tert*-butylsulfinyl)-2-(2-methylbut-3-en-2-yl)pyrrolidine (*R*,*S*-7a)

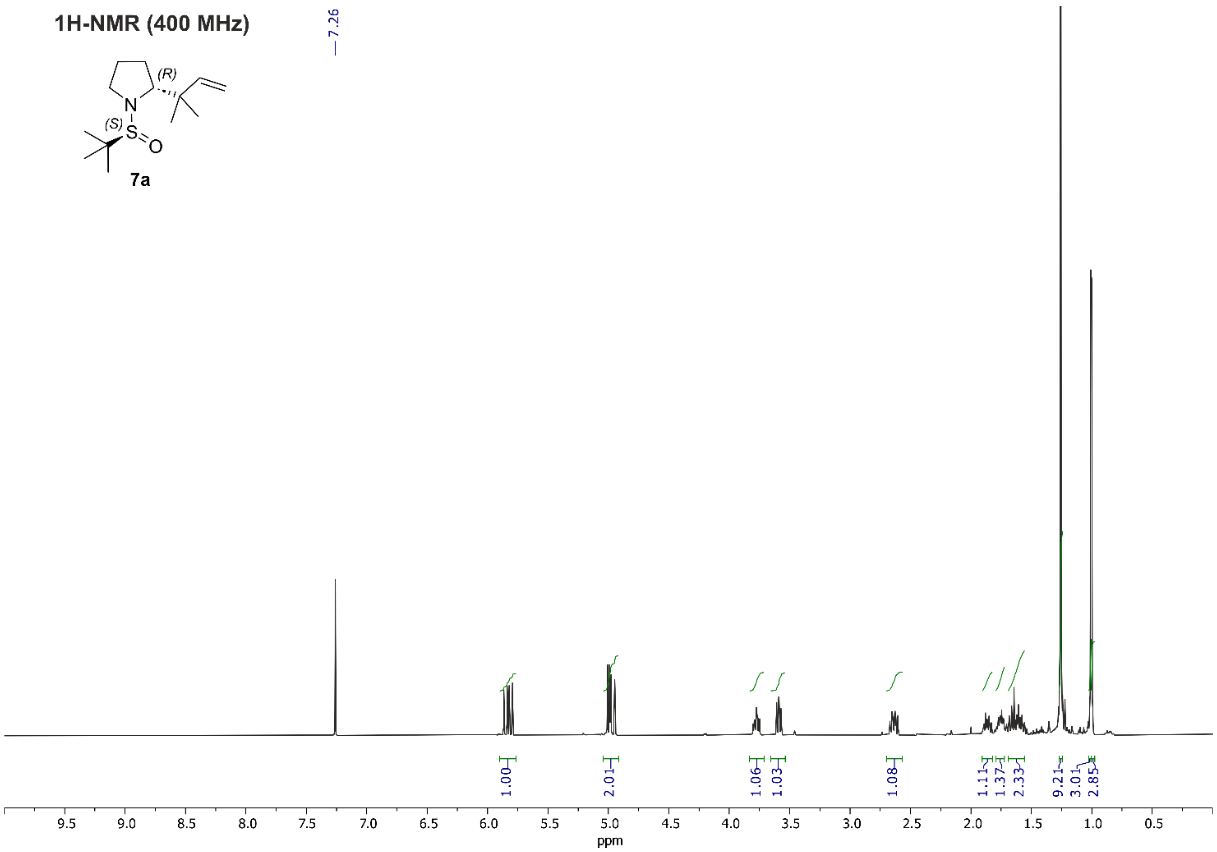

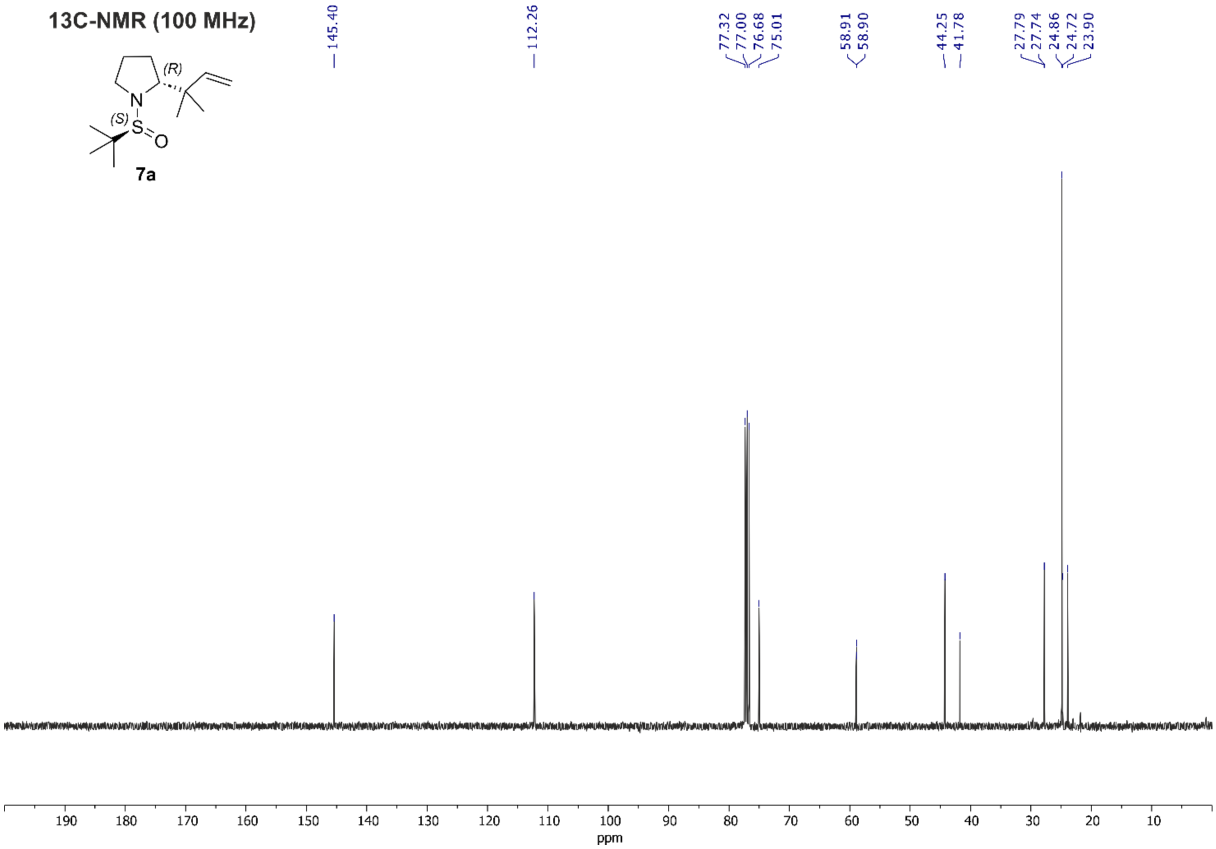
13C-NMR (100 MHz)
(R)
N
(S)
S
O
7a
145.40
112.26
77.32
77.00
76.68
75.01
58.91
58.90
44.25
41.78
27.79
27.74
24.86
24.72
23.90
190 180 170 160 150 140 130 120 110 100 90 80 70 60 50 40 30 20 10
ppm

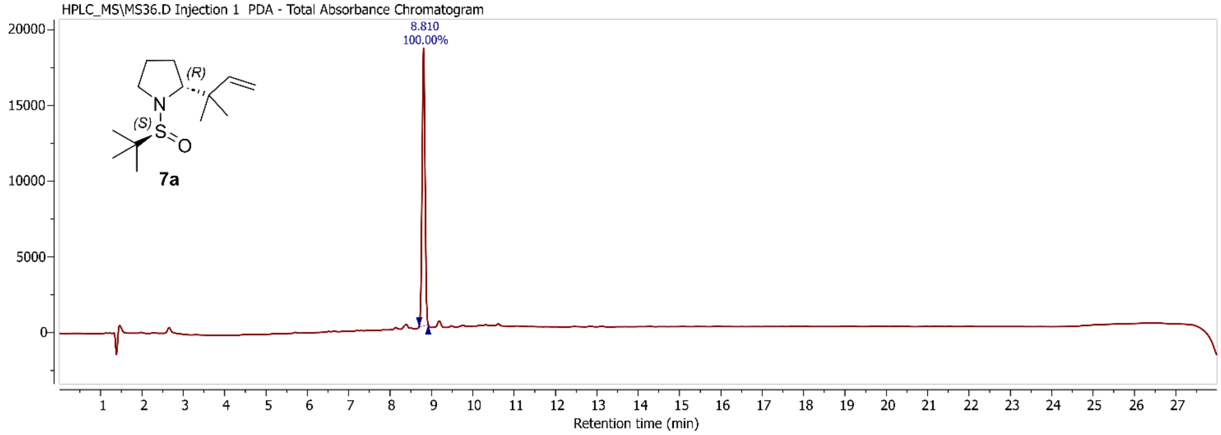
HPLC_MS\MS36.D Injection 1 PDA - Total Absorbance Chromatogram
8.810
100.00%
(R)
N
(S)
S
O
7a
20000
15000
10000
5000
0
1 2 3 4 5 6 7 8 9 10 11 12 13 14 15 16 17 18 19 20 21 22 23 24 25 26 27
Retention time (min)

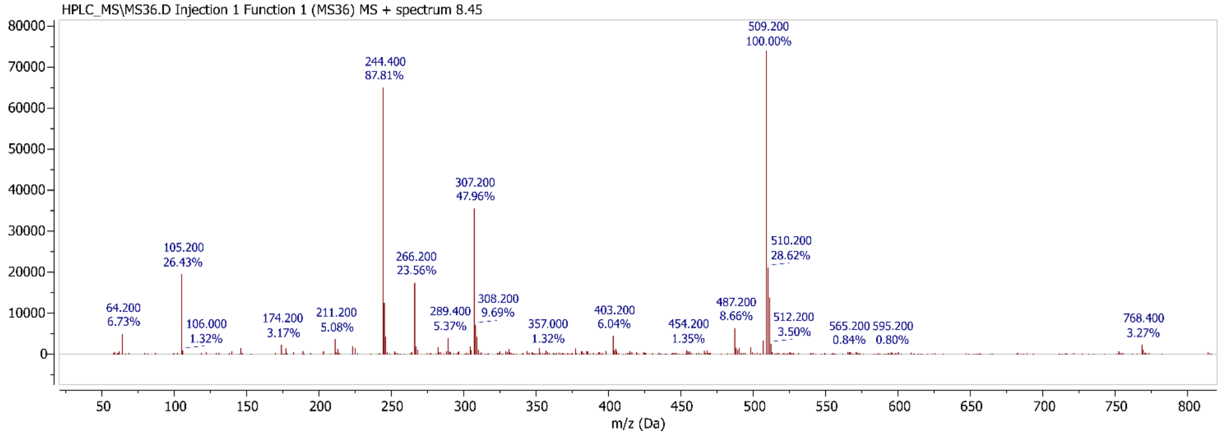
HPLC_MS\MS36.D Injection 1 Function 1 (MS36) MS + spectrum 8.45
509.200
100.00%
244.400
87.81%
307.200
47.96%
510.200
28.62%
105.200
26.43%
266.200
23.56%
64.200
6.73%
106.000
1.32%
174.200
3.17%
211.200
5.08%
289.400
5.37%
308.200
9.69%
357.000
1.32%
403.200
6.04%
454.200
1.35%
487.200
8.66%
512.200
3.50%
565.200
0.84%
595.200
0.80%
768.400
3.27%
80000
70000
60000
50000
40000
30000
20000
10000
0
50 100 150 200 250 300 350 400 450 500 550 600 650 700 750 800
m/z (Da)

(*S*)-1-((*S*)-tert-butylsulfinyl)-2-(2-methylbut-3-en-2-yl)pyrrolidine (*S*,*S*-7a)

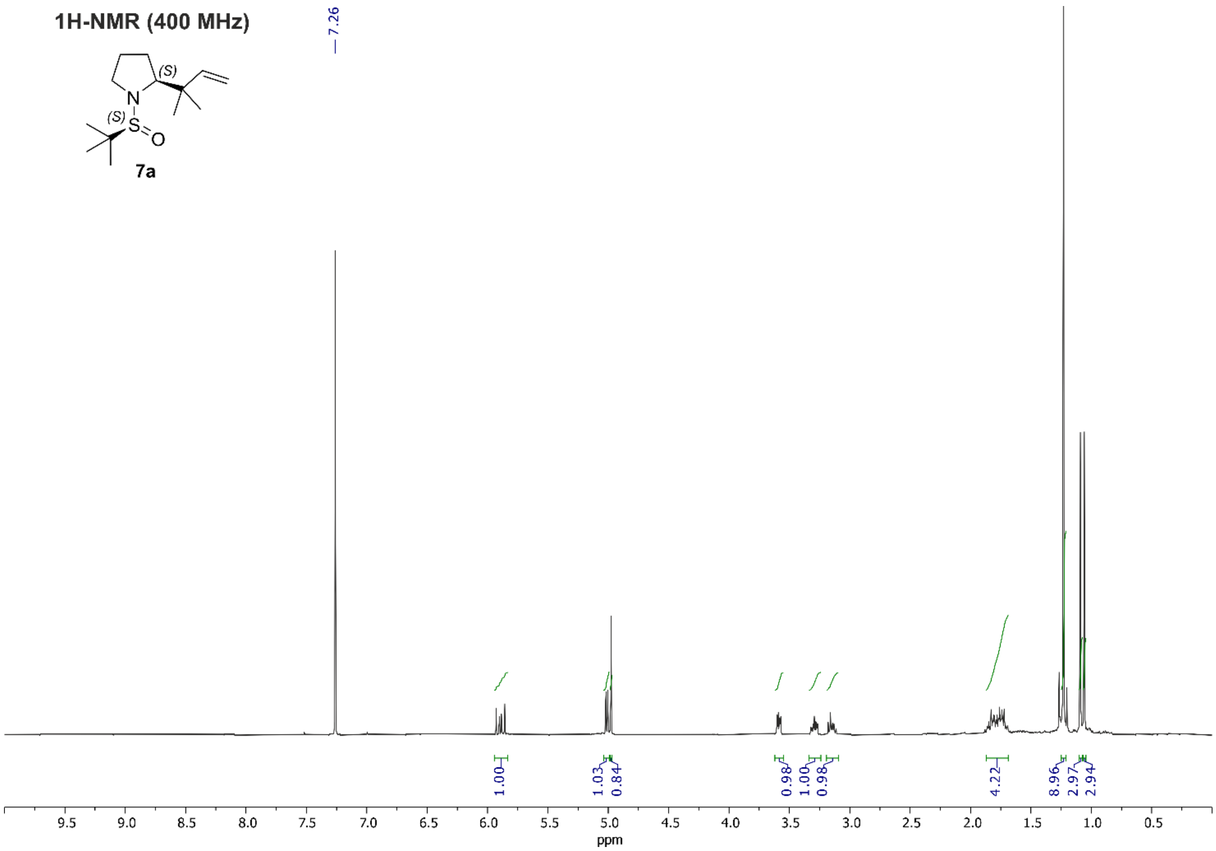

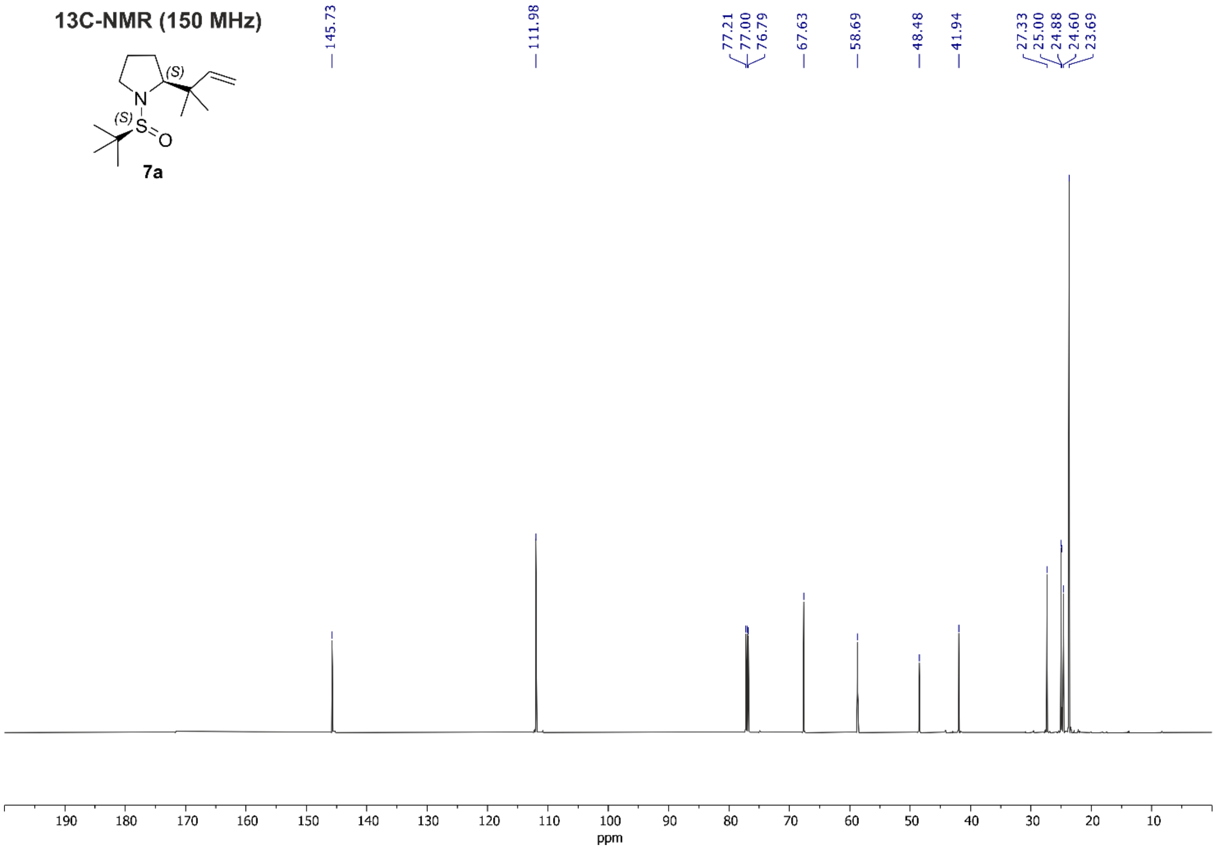

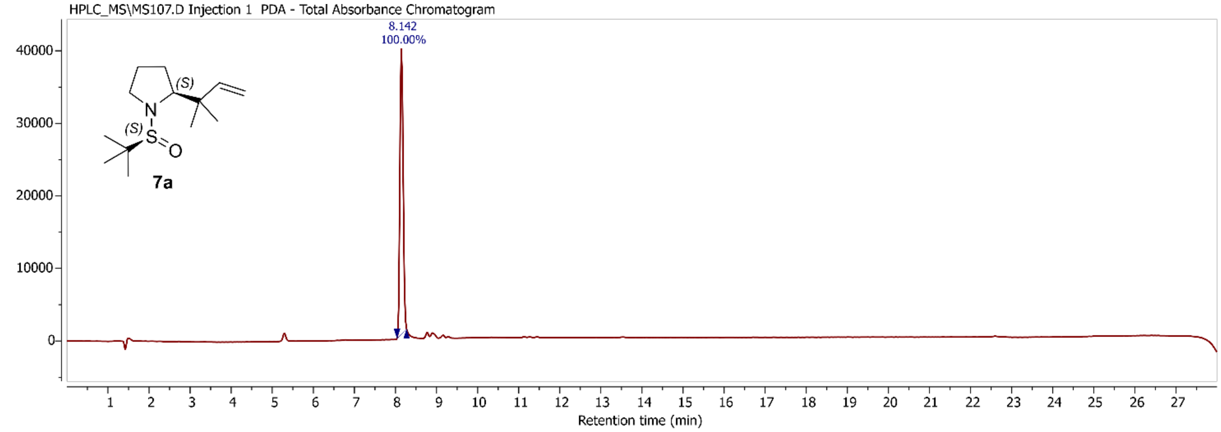
HPLC_MS\MS107.D Injection 1 PDA - Total Absorbance Chromatogram
8.142
100.00%
7a
Retention time (min)

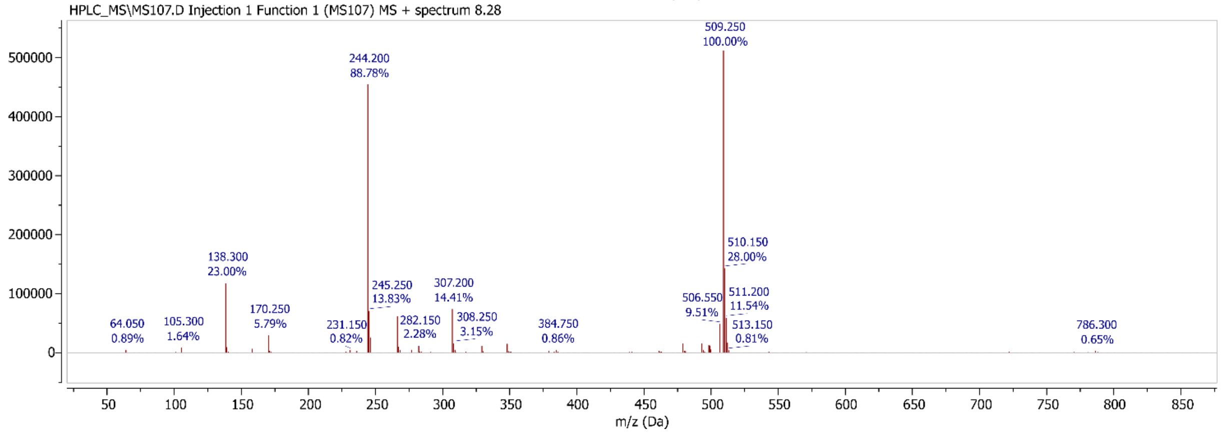
HPLC_MS\MS107.D Injection 1 Function 1 (MS107) MS + spectrum 8.28
509.250
100.00%
244.200
88.78%
138.300
23.00%
510.150
28.00%
511.200
11.54%
245.250
13.83%
307.200
14.41%
506.550
9.51%
170.250
5.79%
282.150
2.28%
308.250
3.15%
64.050
0.89%
105.300
1.64%
231.150
0.82%
384.750
0.86%
513.150
0.81%
786.300
0.65%
m/z (Da)

(*S*)-1-((*R*)-tert-butylsulfinyl)-2-(1-vinylcyclopentyl)pyrrolidine (*R*,*S*-7b)

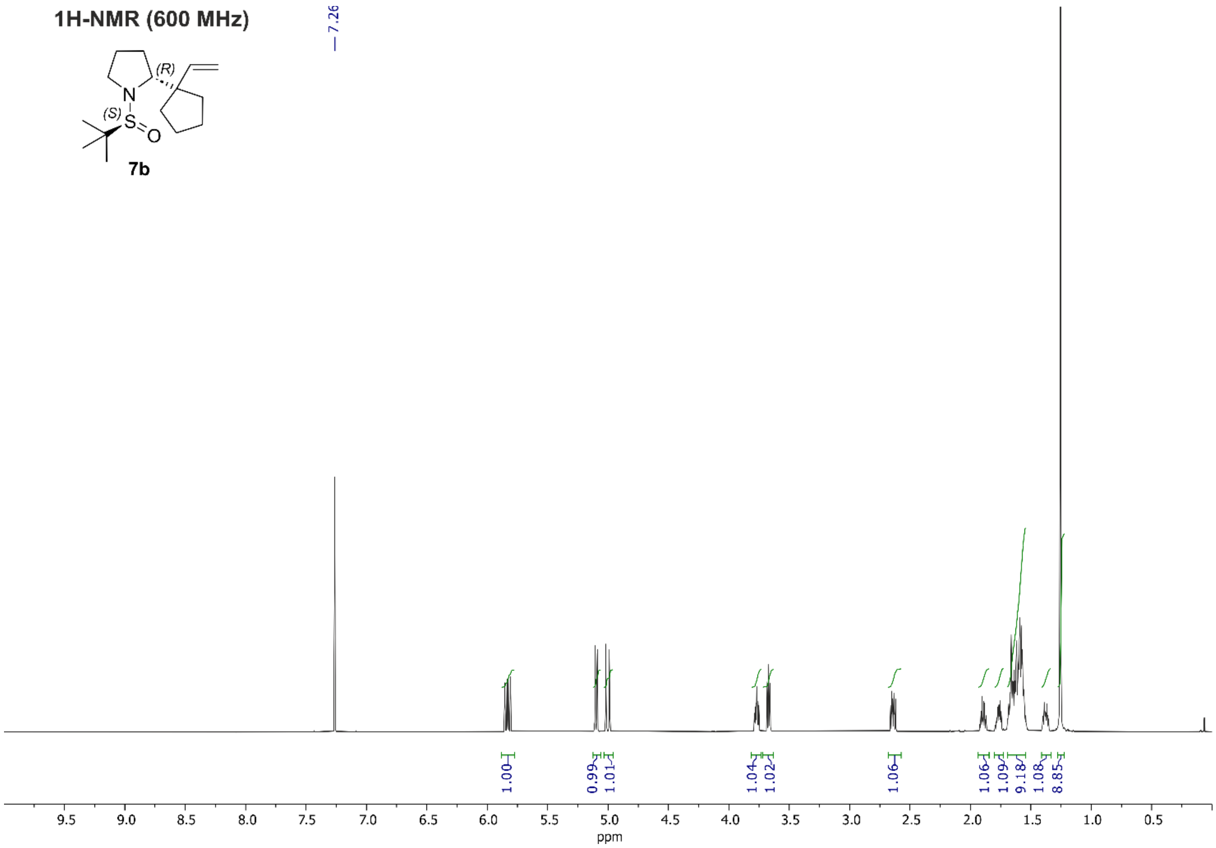

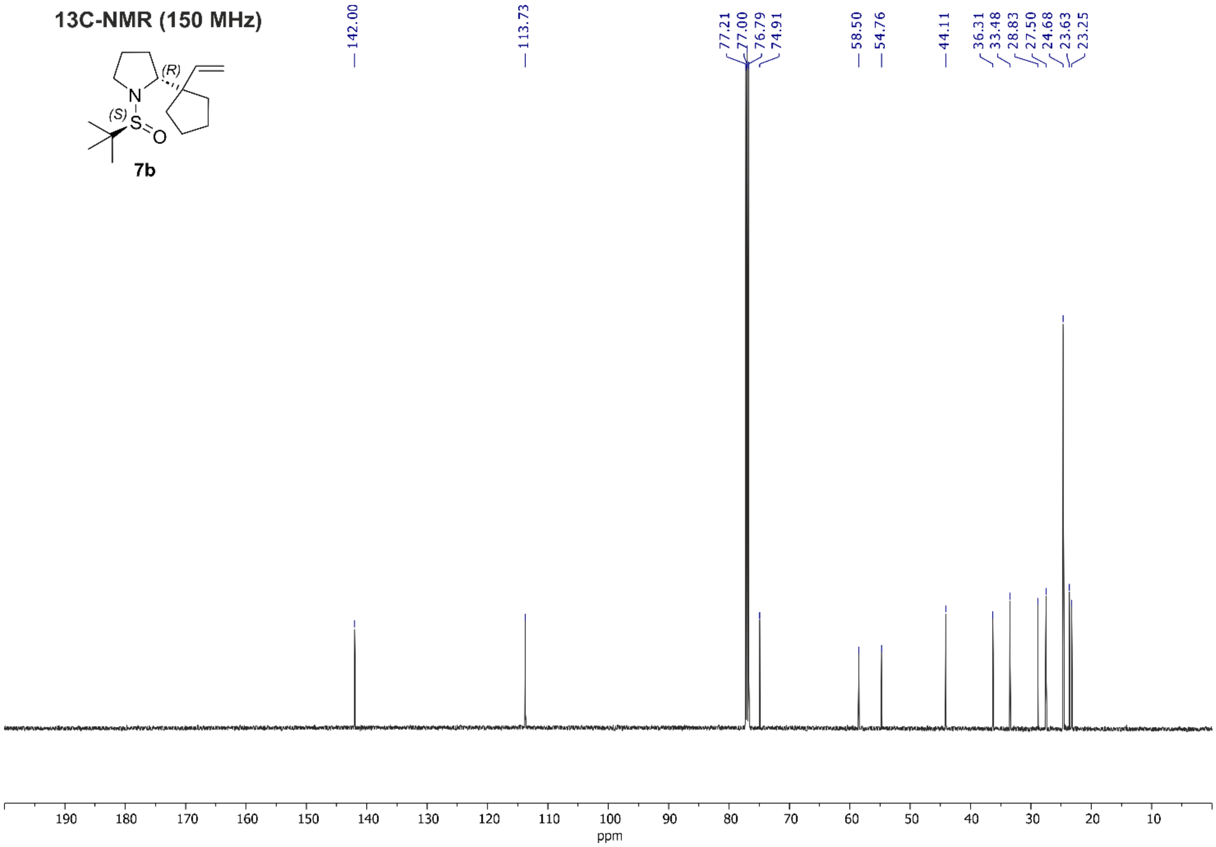

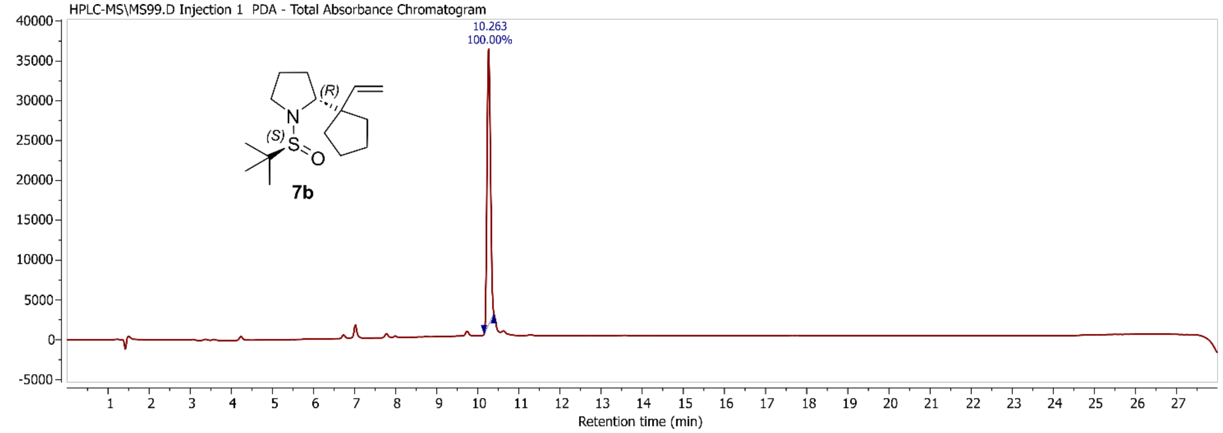

HPLC-MS\MS99.D Injection 1 PDA - Total Absorbance Chromatogram
10.263
100.00%
(R)
N
(S)
S
O
7b
Retention time (min)

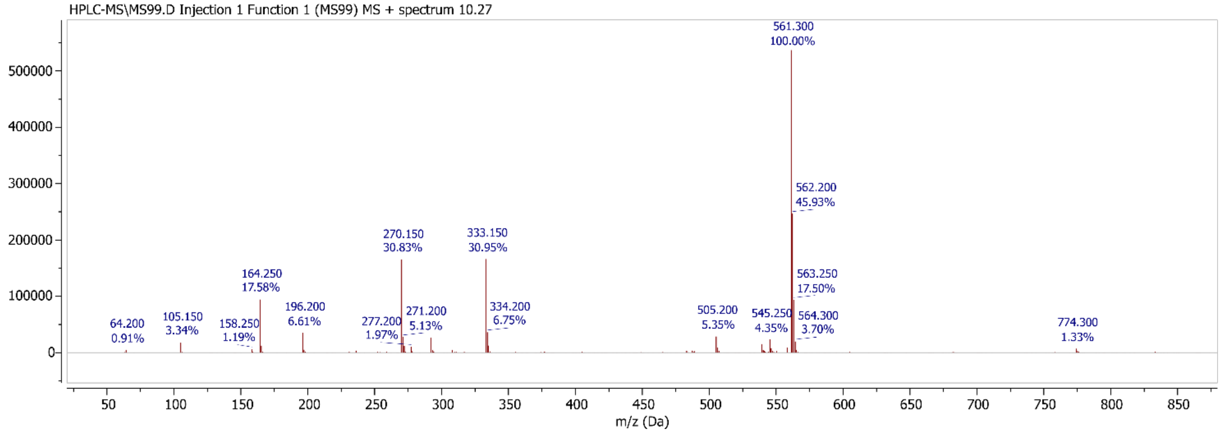

HPLC-MS\MS99.D Injection 1 Function 1 (MS99) MS + spectrum 10.27
561.300
100.00%
562.200
45.93%
563.250
17.50%
564.300
3.70%
270.150
30.83%
333.150
30.95%
164.250
17.58%
196.200
6.61%
271.200
5.13%
277.200
1.97%
334.200
6.75%
105.150
3.34%
158.250
1.19%
64.200
0.91%
505.200
5.35%
545.250
4.35%
774.300
1.33%
m/z (Da)

(*S*)-1-((*S*)-tert-butylsulfinyl)-2-(1-vinylcyclopentyl)pyrrolidine (*S*,*S*-7b)

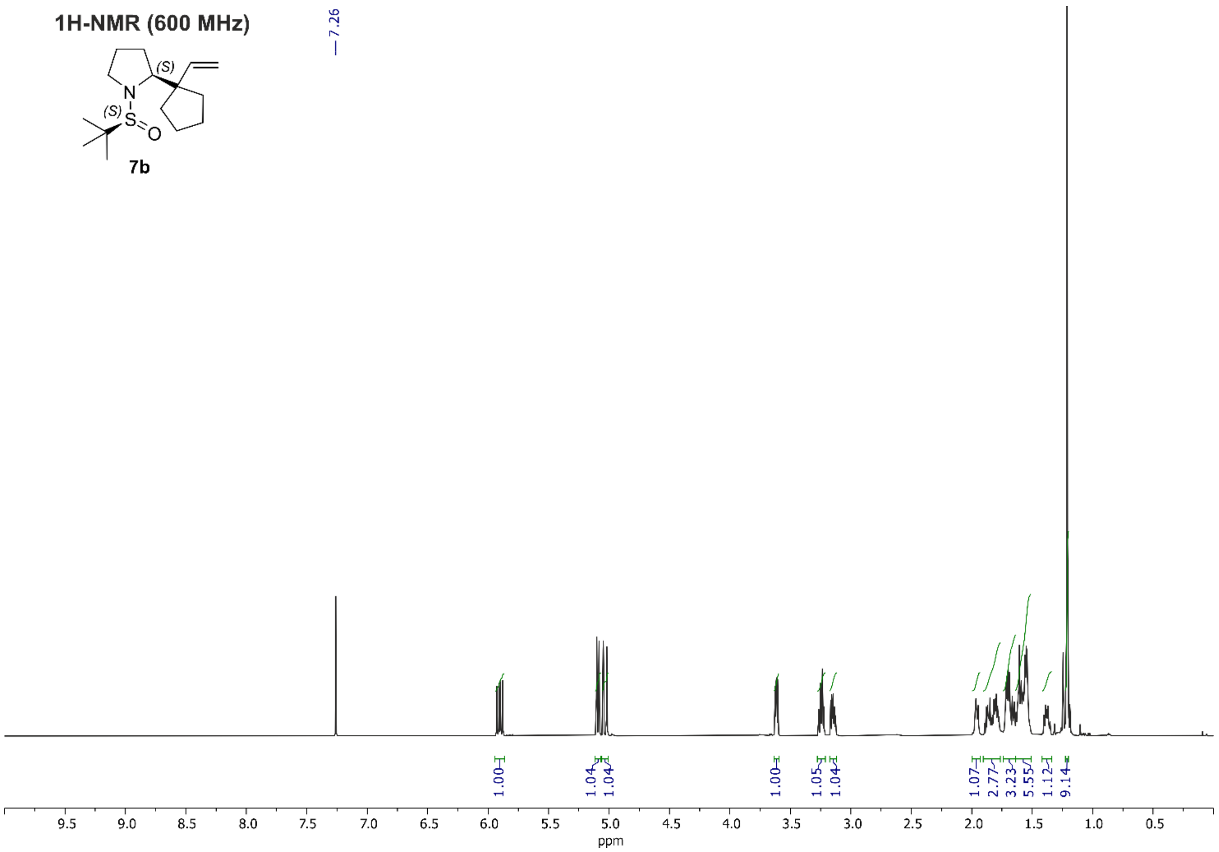

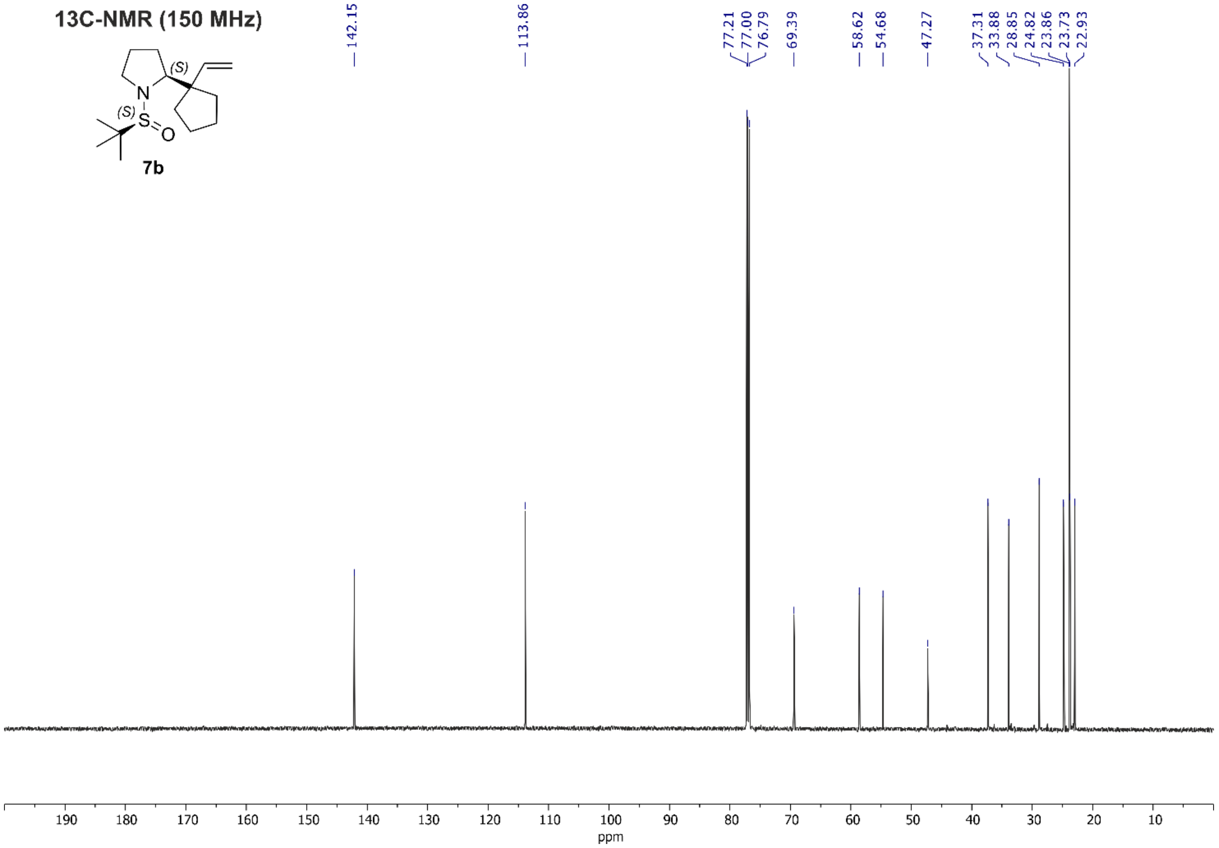

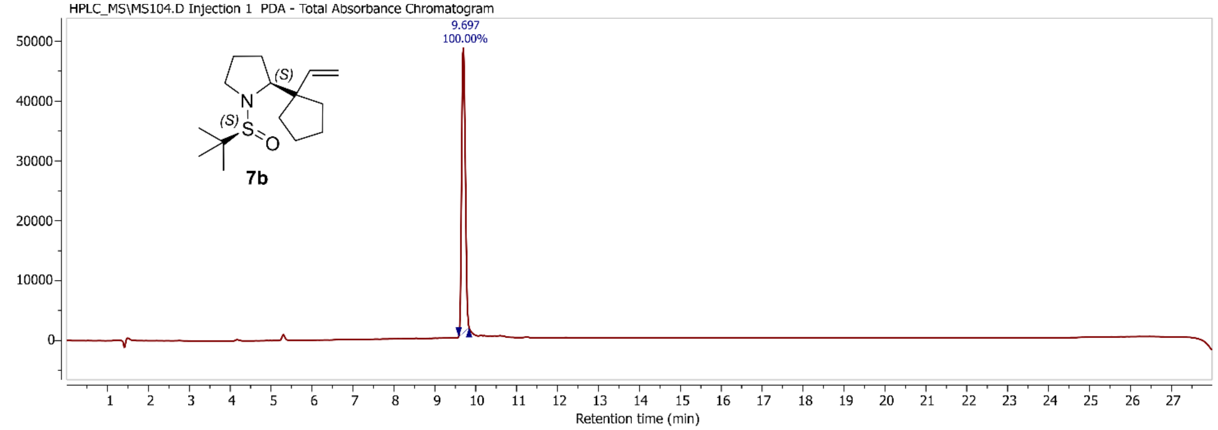
HPLC_MS\MS104.D Injection 1 PDA - Total Absorbance Chromatogram
9.697
100.00%
(S)
N
(S)
S
O
7b
Retention time (min)

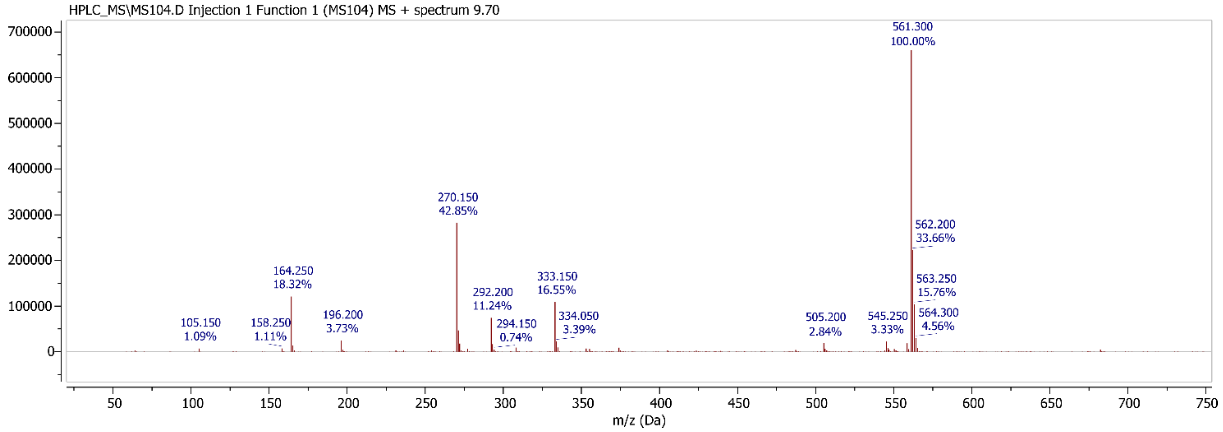
HPLC_MS\MS104.D Injection 1 Function 1 (MS104) MS + spectrum 9.70
561.300
100.00%
270.150
42.85%
562.200
33.66%
164.250
18.32%
333.150
16.55%
563.250
15.76%
292.200
11.24%
564.300
4.56%
105.150
1.09%
158.250
1.11%
196.200
3.73%
294.150
0.74%
334.050
3.39%
505.200
2.84%
545.250
3.33%
m/z (Da)

(*R*)-1-((*S*)-*tert*-butylsulfinyl)-2-(1-vinylcyclohexyl)pyrrolidine (*R*,*S*-7c)

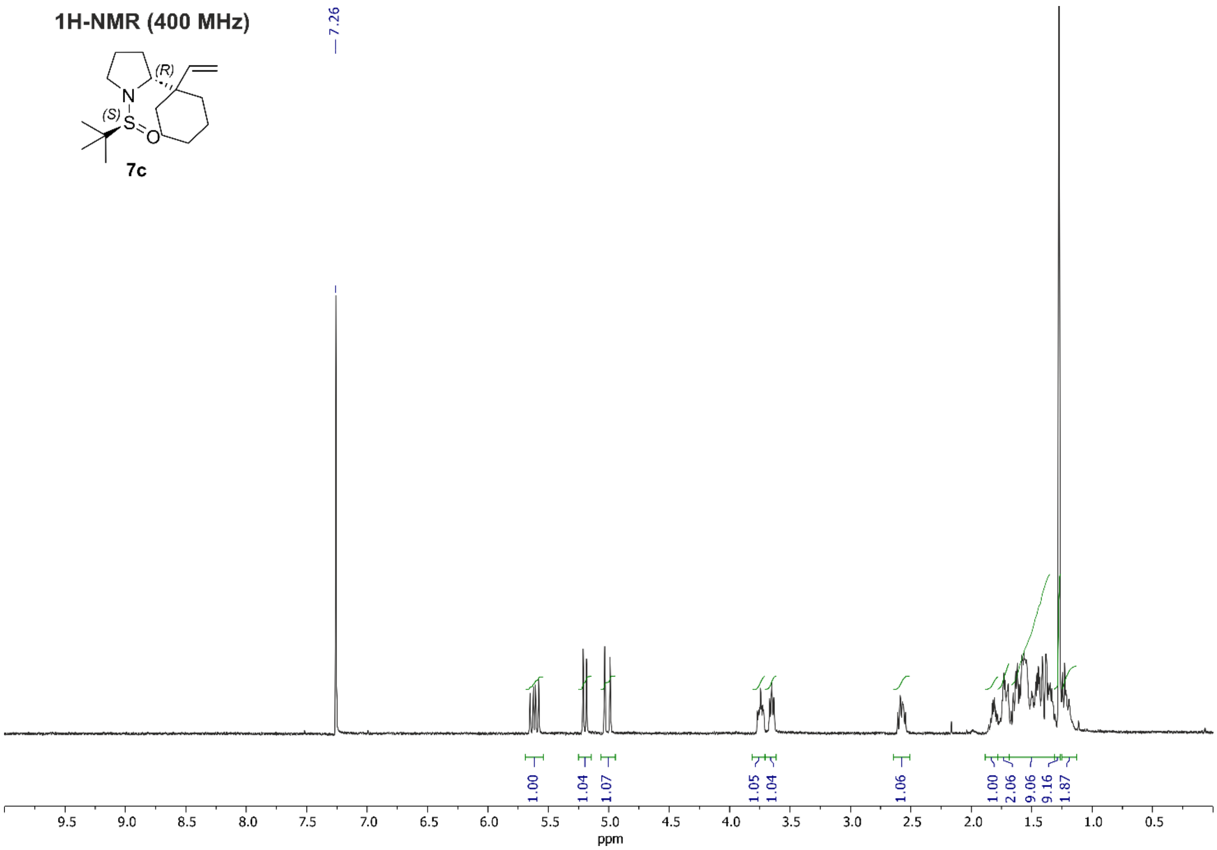

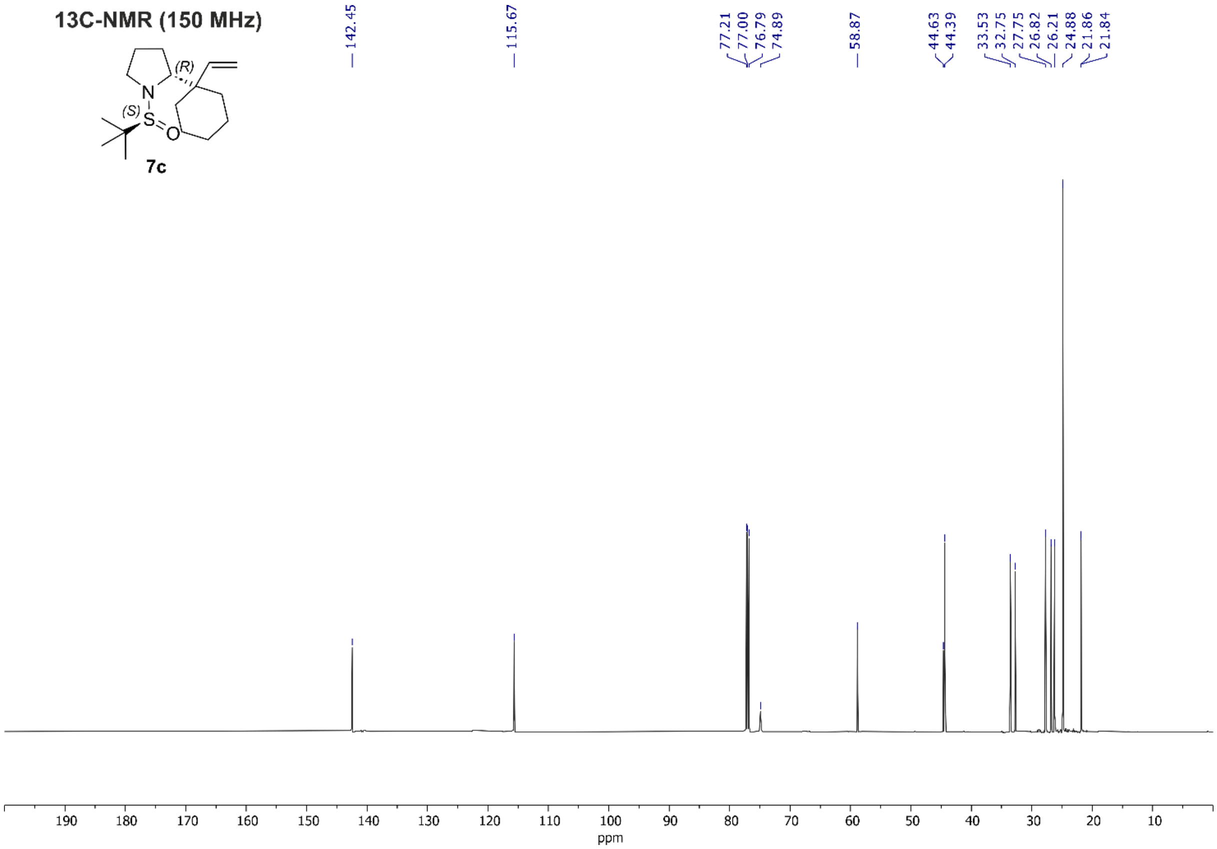

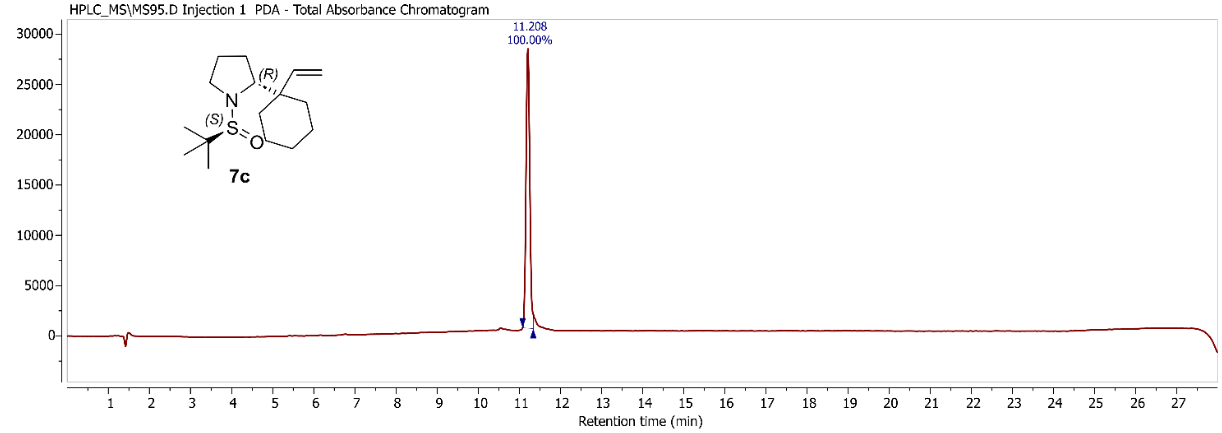
HPLC_MS\MS95.D Injection 1 PDA - Total Absorbance Chromatogram
11.208
100.00%
(R)
N
(S)
S
O
7c
Retention time (min)

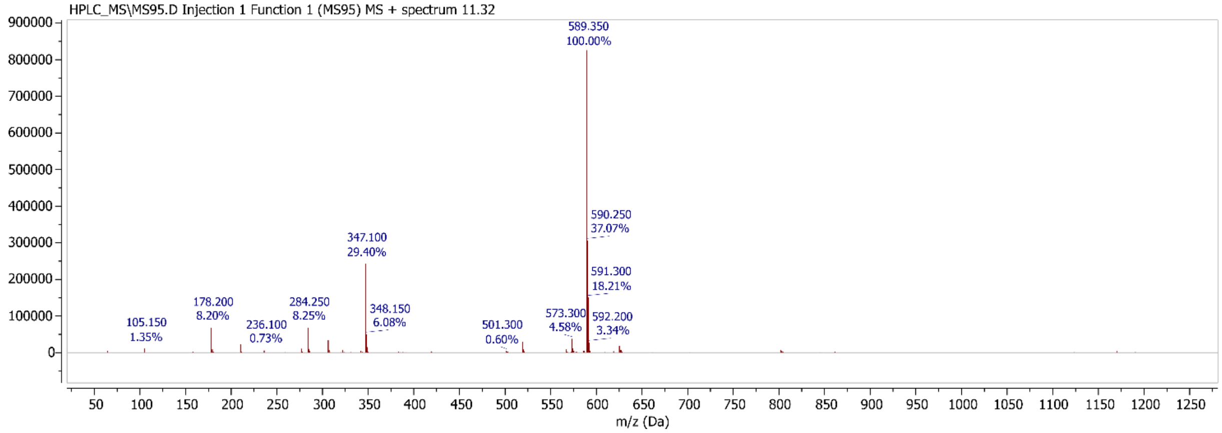
HPLC_MS\MS95.D Injection 1 Function 1 (MS95) MS + spectrum 11.32
589.350
100.00%
590.250
37.07%
591.300
18.21%
592.200
3.34%
573.300
4.58%
501.300
0.60%
347.100
29.40%
348.150
6.08%
284.250
8.25%
236.100
0.73%
178.200
8.20%
105.150
1.35%
m/z (Da)

(*R*)-1-((*S*)-*tert*-butylsulfinyl)-2-(1-vinylcycloheptyl)pyrrolidine (*R*,*S*-7d)

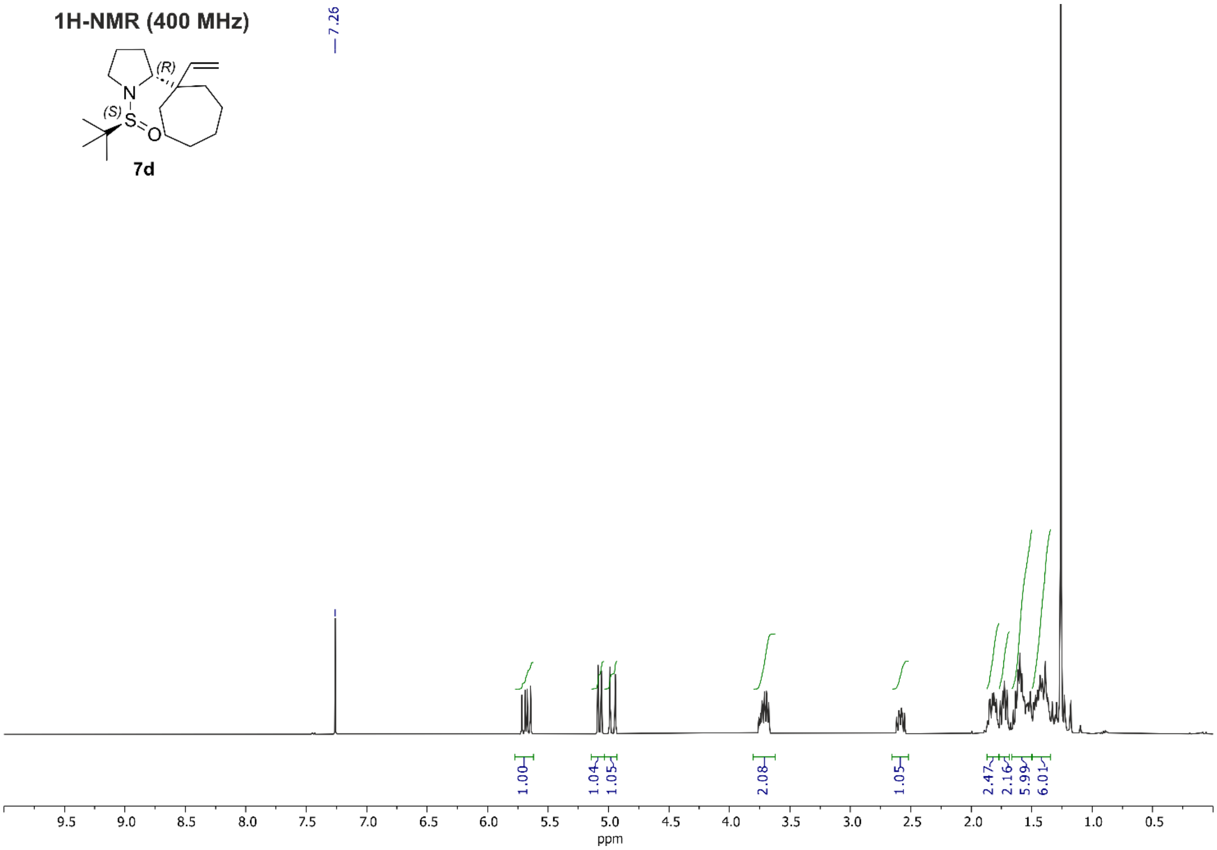

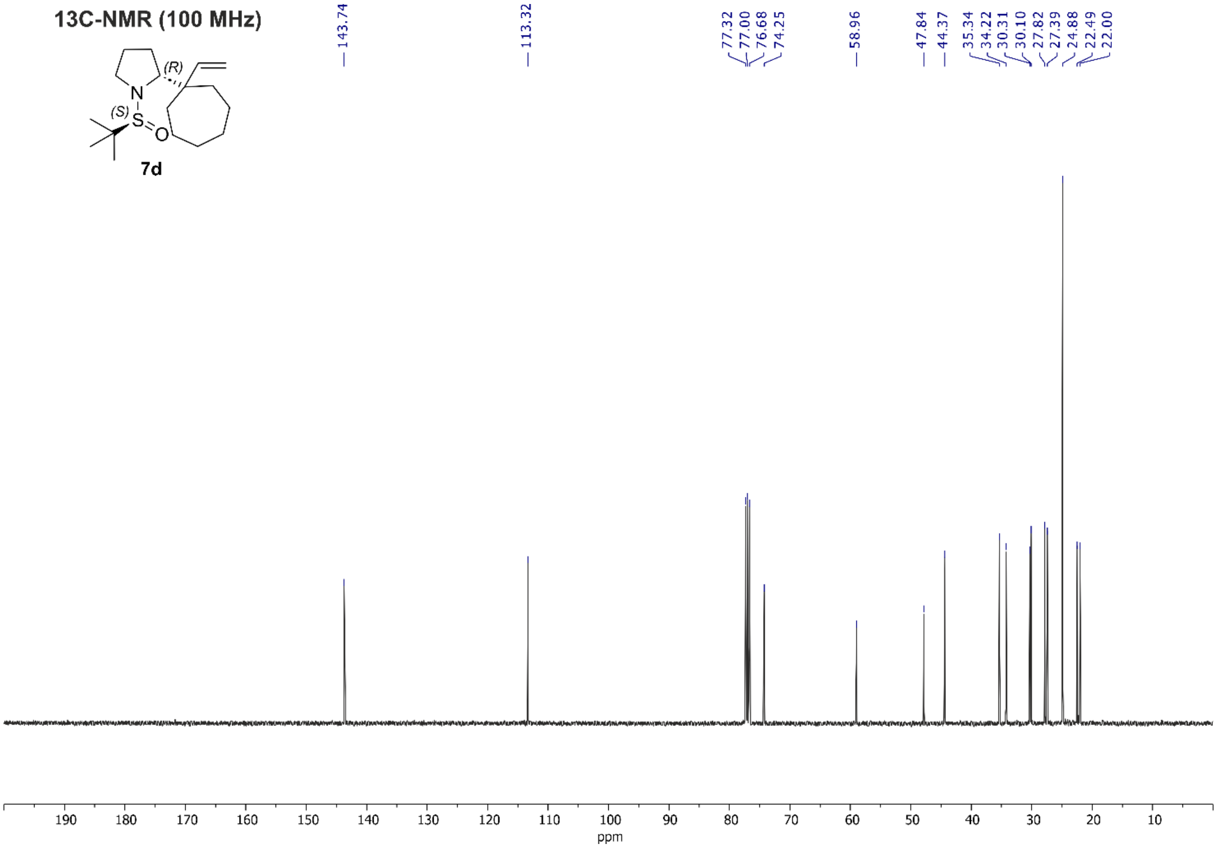

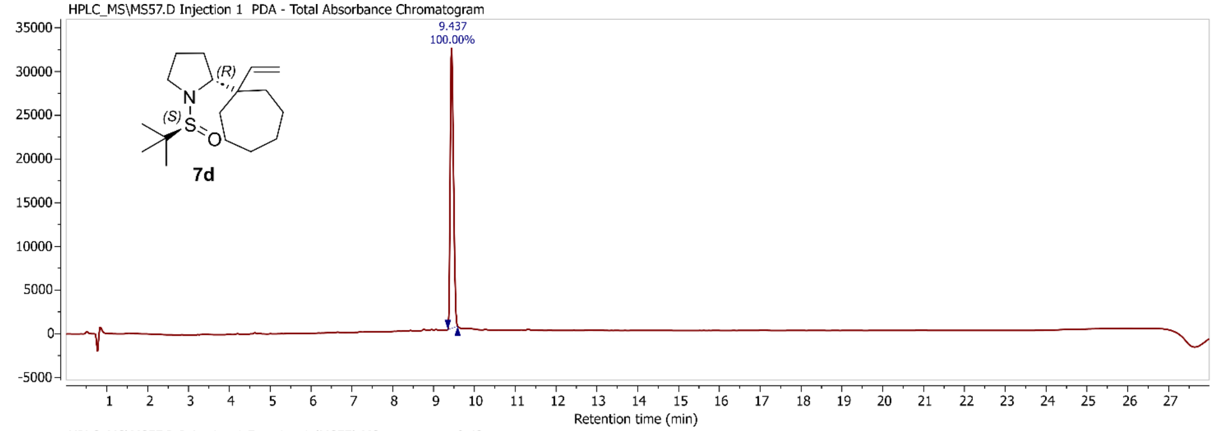
HPLC_MS\MS57.D Injection 1 PDA - Total Absorbance Chromatogram
9.437
100.00%
(R)
(S)
7d
Retention time (min)

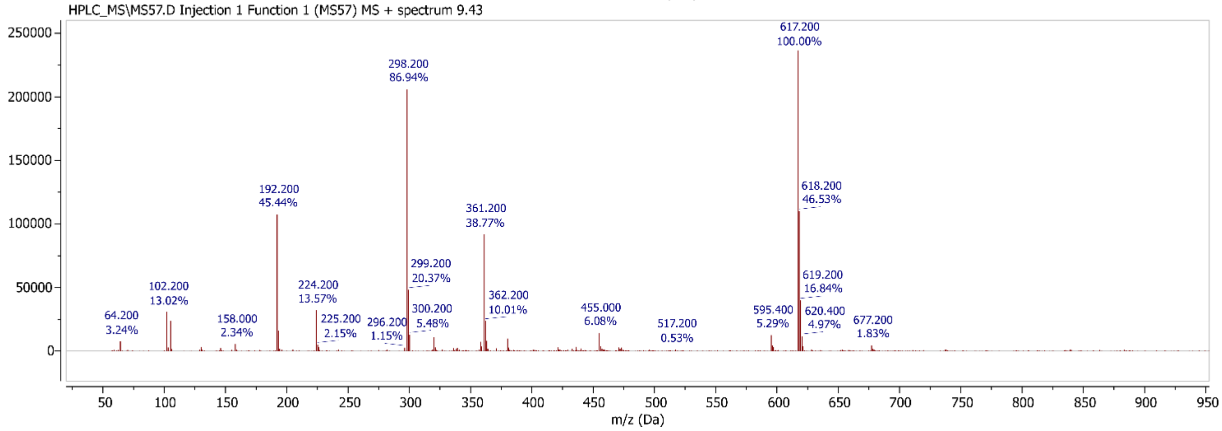
HPLC_MS\MS57.D Injection 1 Function 1 (MS57) MS + spectrum 9.43
617.200
100.00%
298.200
86.94%
618.200
46.53%
192.200
45.44%
361.200
38.77%
299.200
20.37%
619.200
16.84%
224.200
13.57%
102.200
13.02%
362.200
10.01%
455.000
6.08%
300.200
5.48%
595.400
5.29%
620.400
4.97%
64.200
3.24%
158.000
2.34%
225.200
2.15%
677.200
1.83%
296.200
1.15%
517.200
0.53%
m/z (Da)

***tert*-butyl (*R*)-2-(2-methylbut-3-en-2-yl)pyrrolidine-1-carboxylate (*R*-9a)**

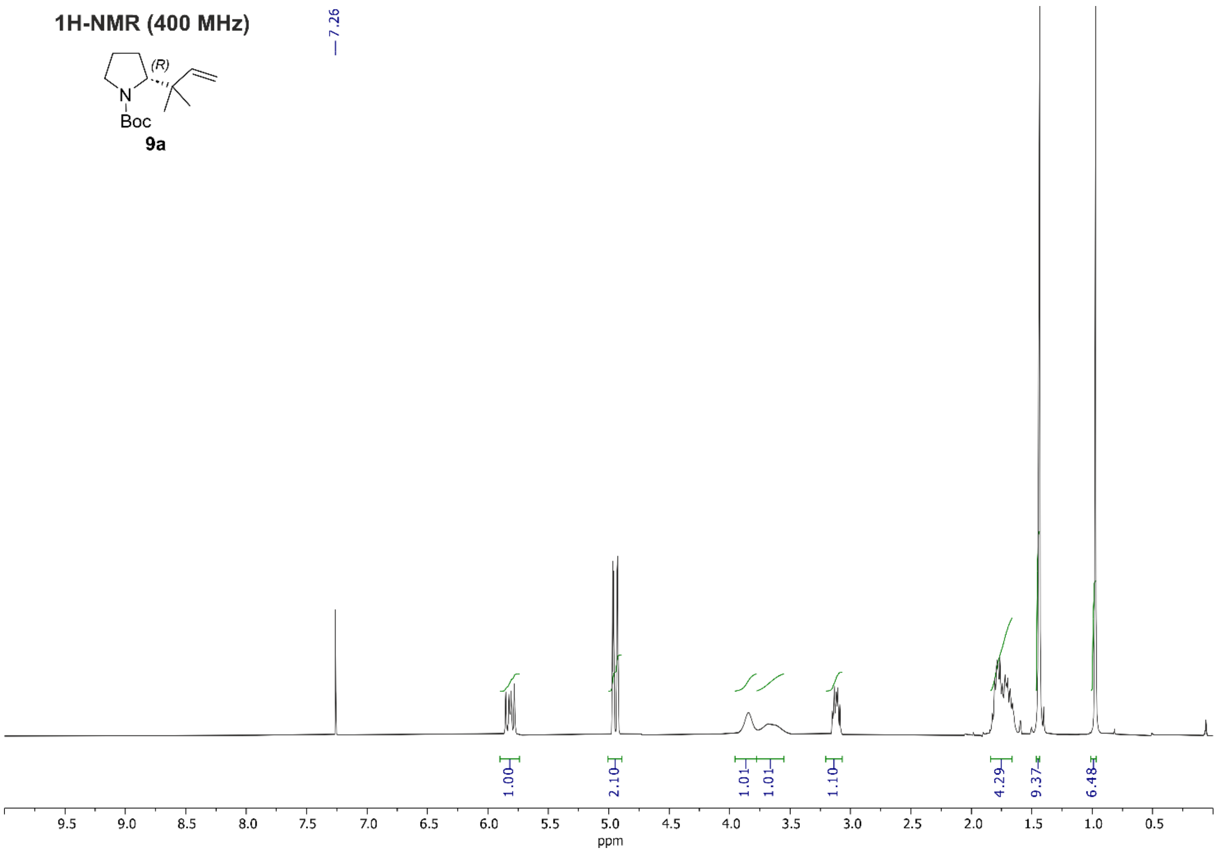

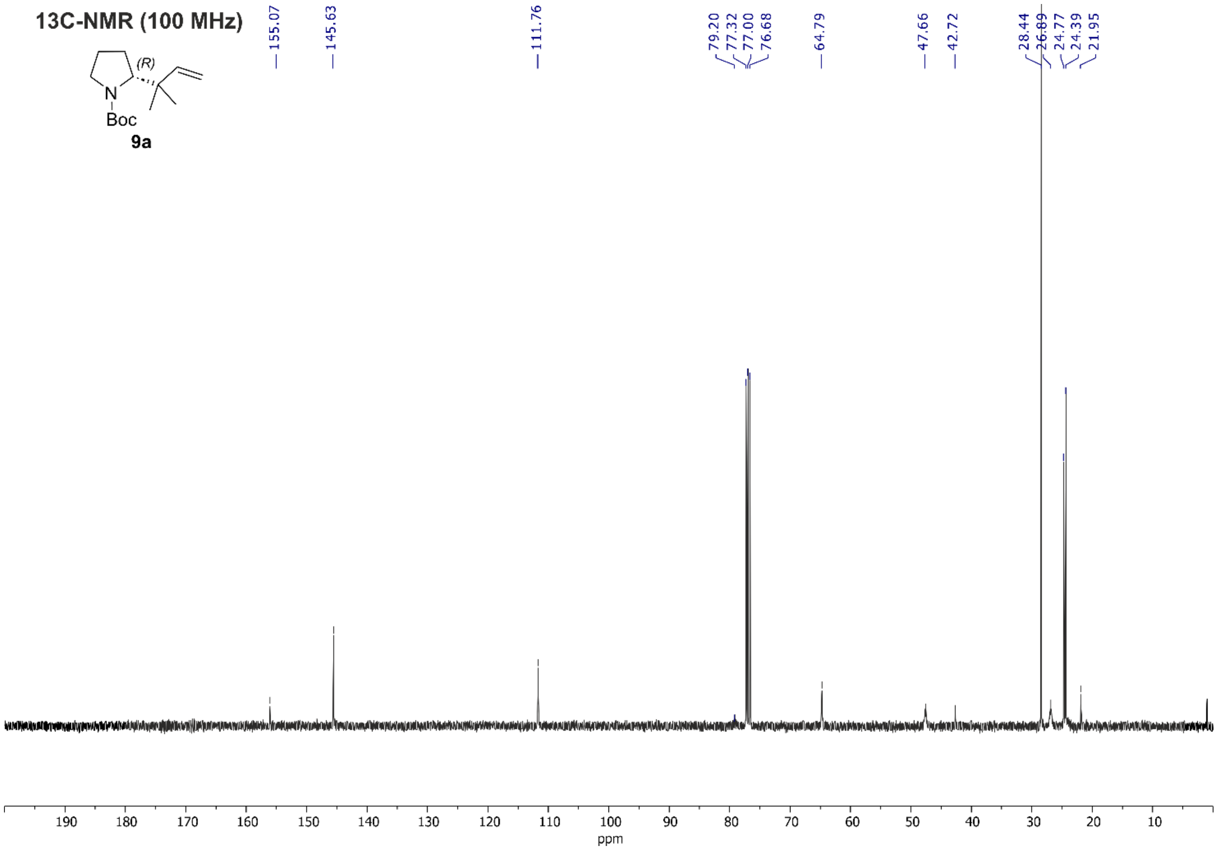

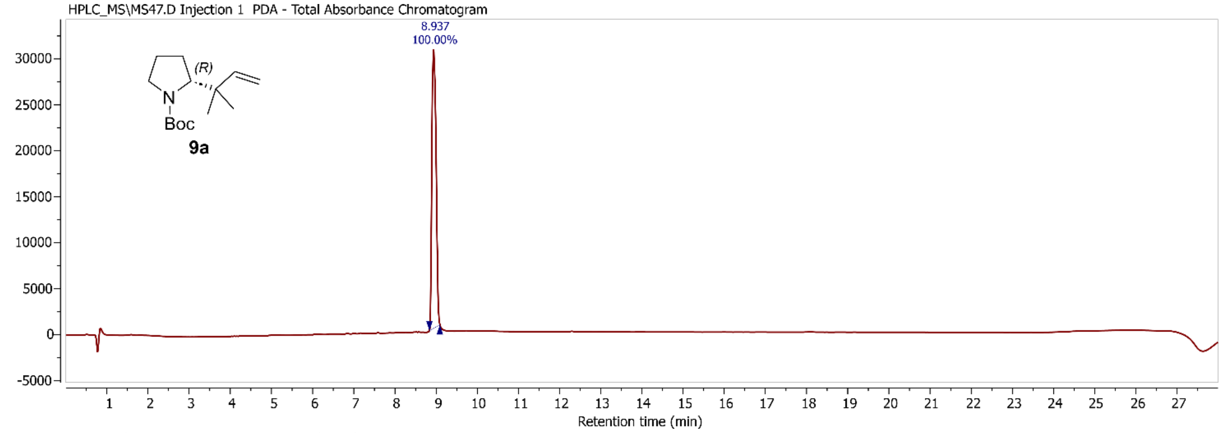

HPLC_MS\MS47.D Injection 1 PDA - Total Absorbance Chromatogram
8.937
100.00%
(R)
N
Boc
9a
Retention time (min)

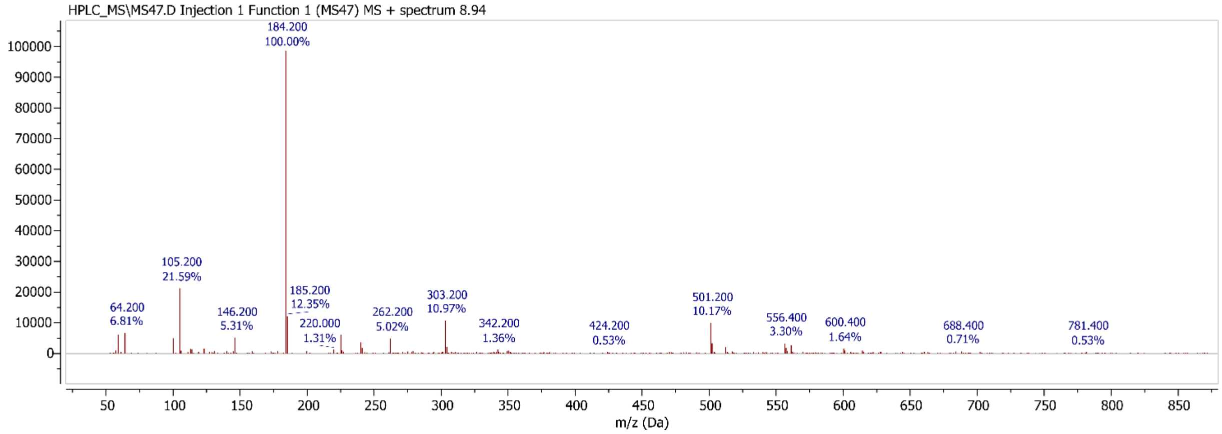

HPLC_MS\MS47.D Injection 1 Function 1 (MS47) MS + spectrum 8.94
184.200
100.00%
105.200
21.59%
64.200
6.81%
146.200
5.31%
185.200
12.35%
220.000
1.31%
262.200
5.02%
303.200
10.97%
342.200
1.36%
424.200
0.53%
501.200
10.17%
556.400
3.30%
600.400
1.64%
688.400
0.71%
781.400
0.53%
m/z (Da)

***tert*-butyl (*R*)-2-(1-vinylcyclopentyl)pyrrolidine-1-carboxylate (*R*-9b)**

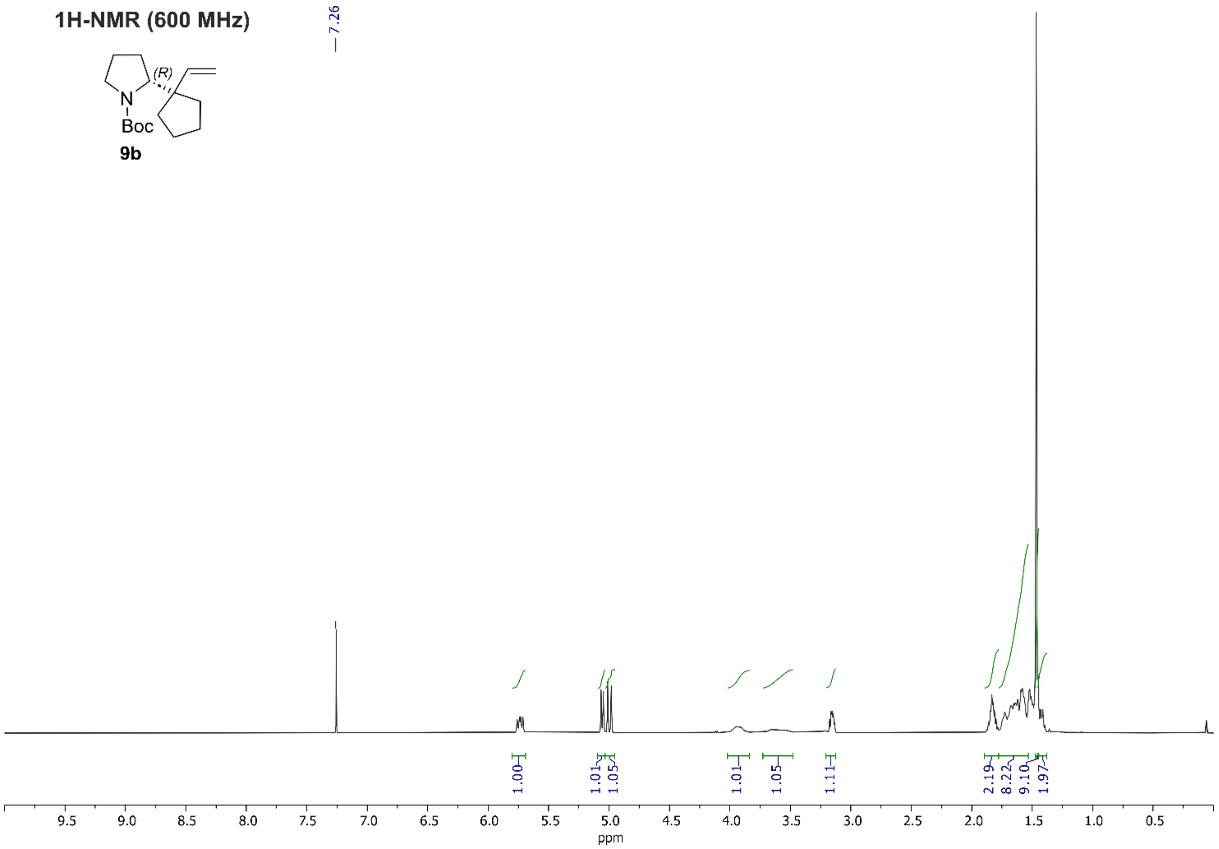

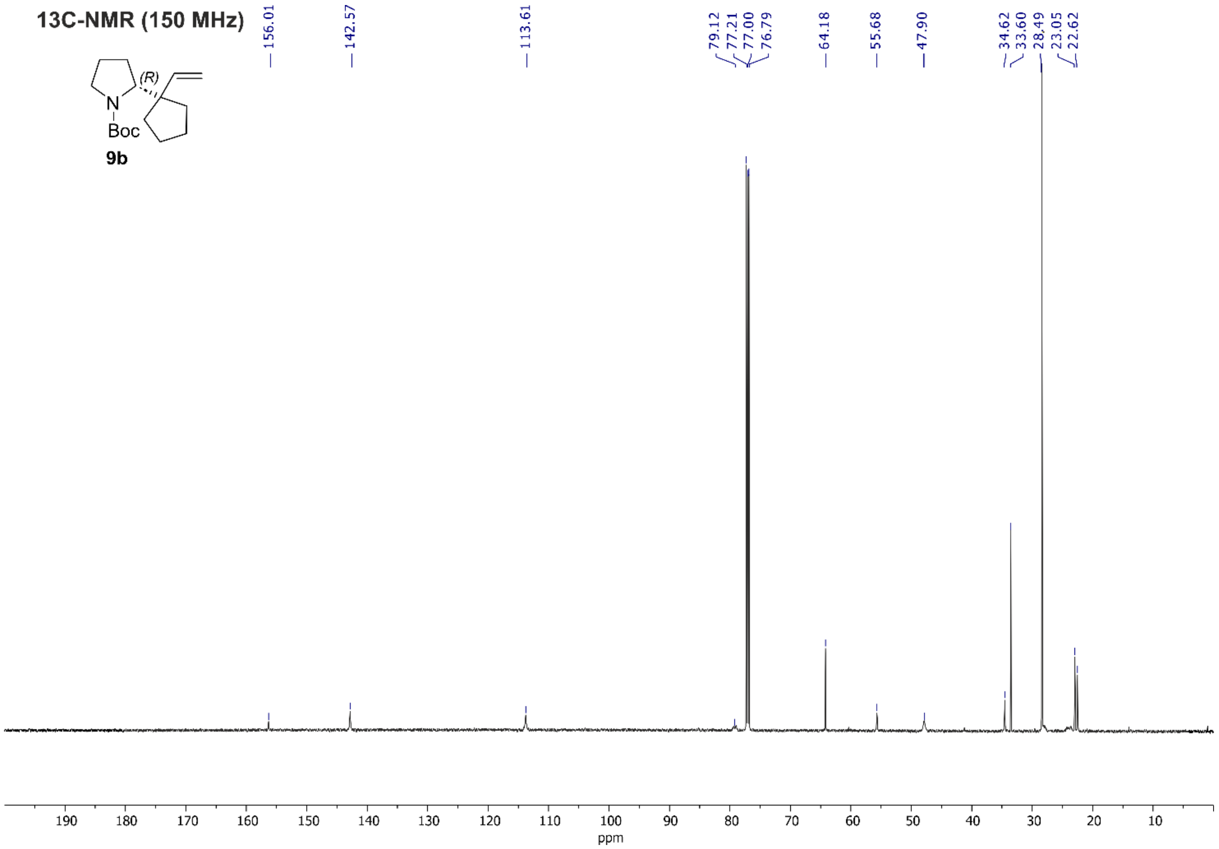

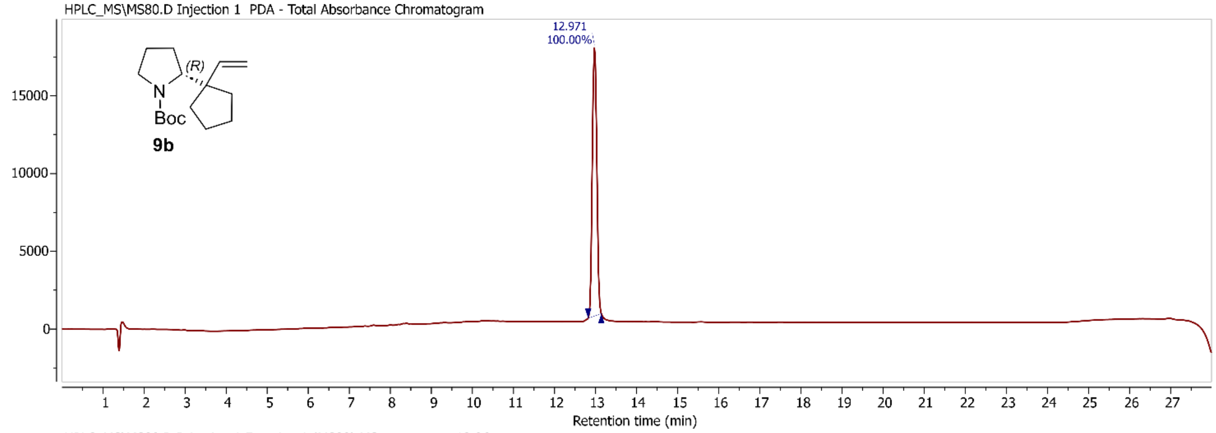
HPLC_MS\MS80.D Injection 1 PDA - Total Absorbance Chromatogram
12.971
100.00%
(R)
N
Boc
9b
Retention time (min)

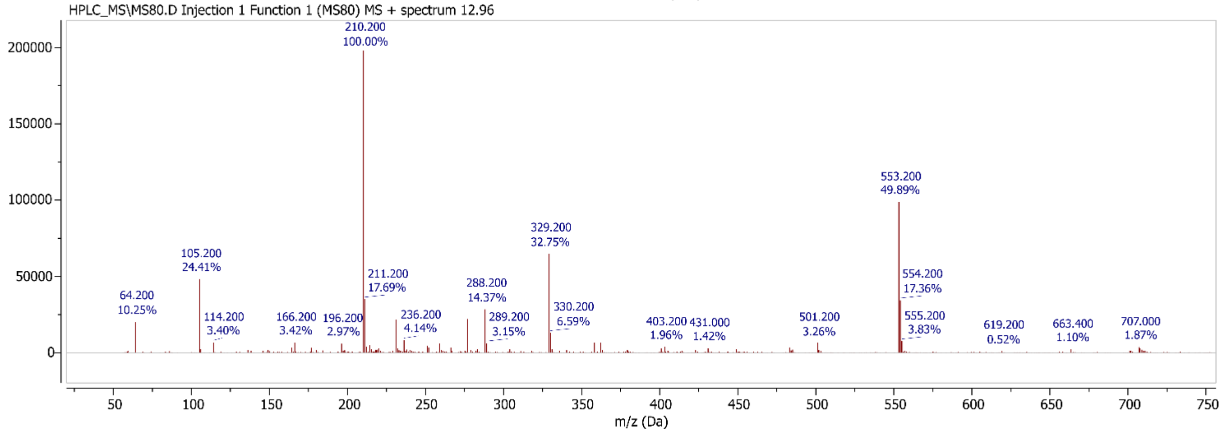
HPLC_MS\MS80.D Injection 1 Function 1 (MS80) MS + spectrum 12.96
210.200
100.00%
553.200
49.89%
329.200
32.75%
105.200
24.41%
211.200
17.69%
288.200
14.37%
554.200
17.36%
64.200
10.25%
330.200
6.59%
114.200
3.40%
166.200
3.42%
196.200
2.97%
236.200
4.14%
289.200
3.15%
403.200
1.96%
431.000
1.42%
501.200
3.26%
555.200
3.83%
619.200
0.52%
663.400
1.10%
707.000
1.87%
m/z (Da)

***tert*-butyl (*R*)-2-(1-vinylcyclohexyl)pyrrolidine-1-carboxylate (*R*-9c)**

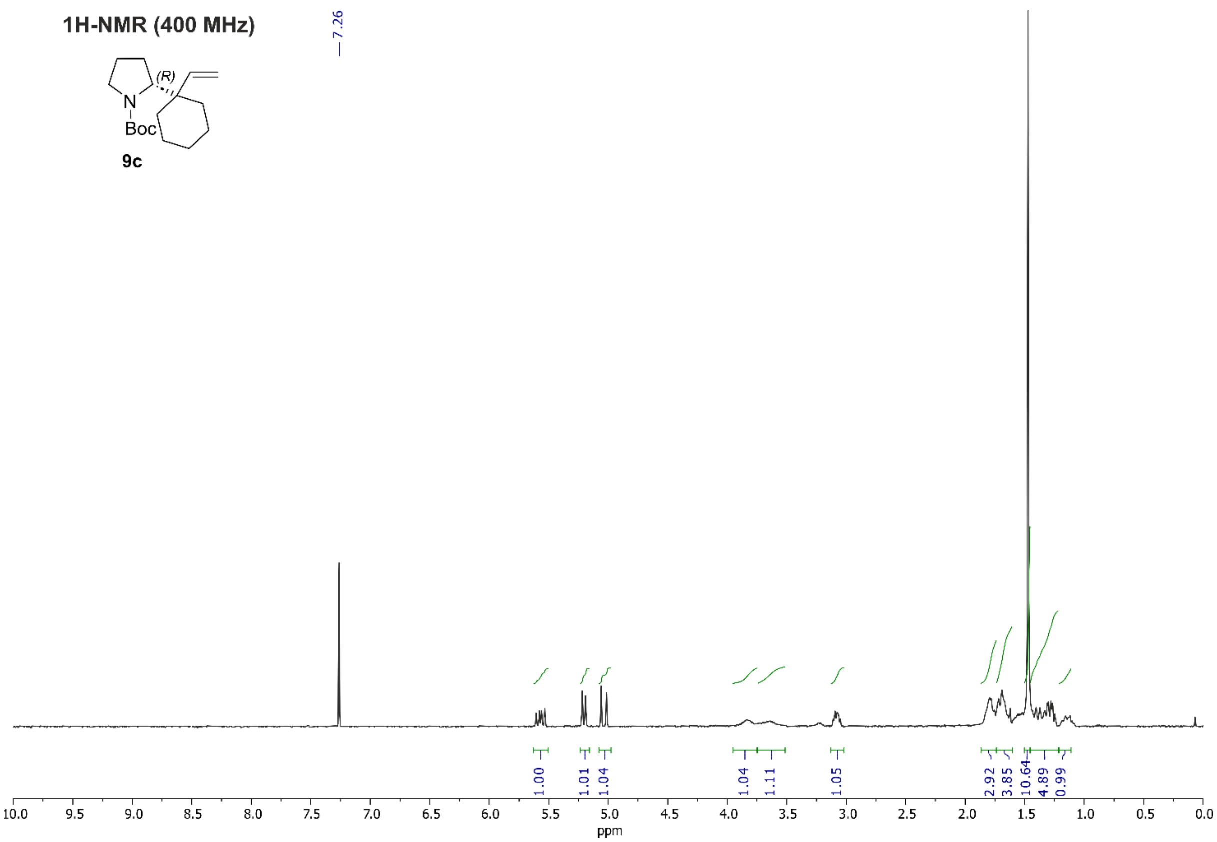

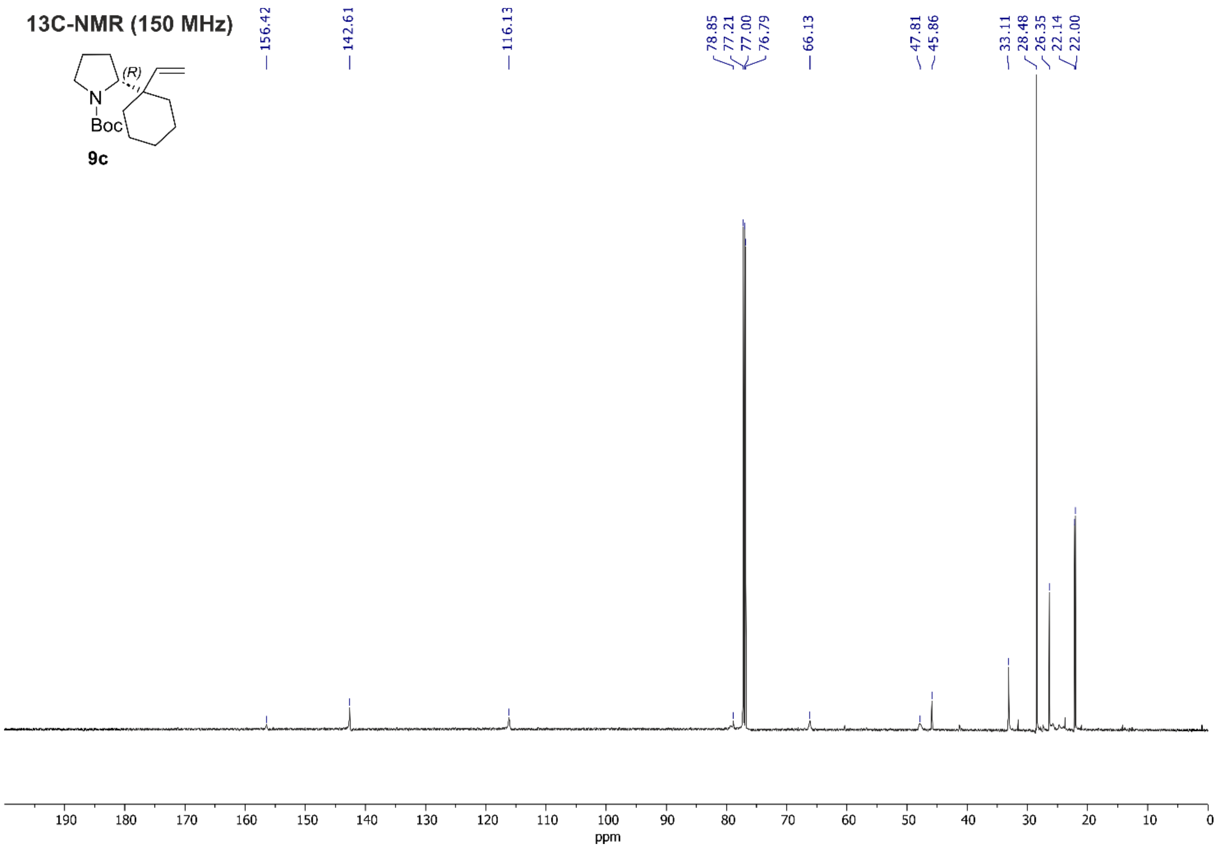

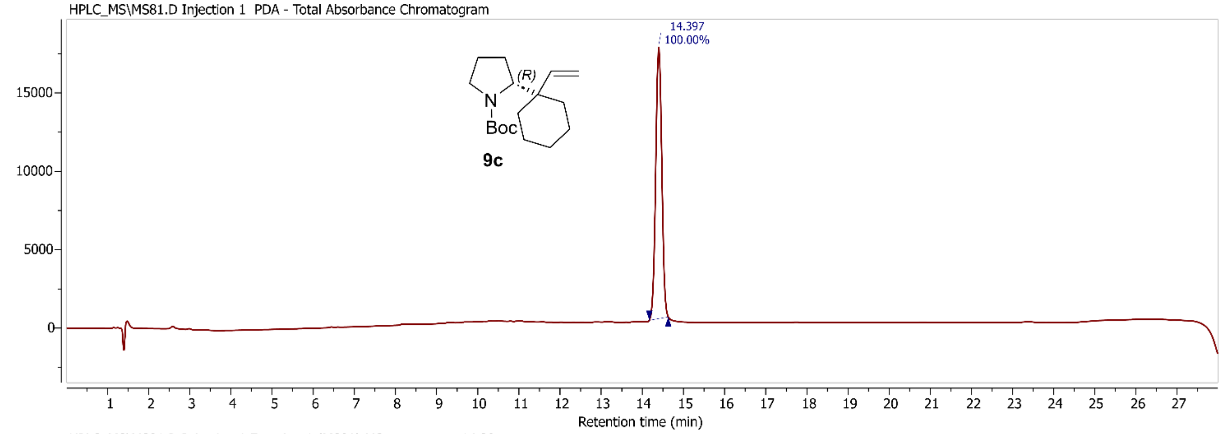
HPLC_MS\MS81.D Injection 1 PDA - Total Absorbance Chromatogram
14.397
100.00%
(R)
N
Boc
9c
Retention time (min)

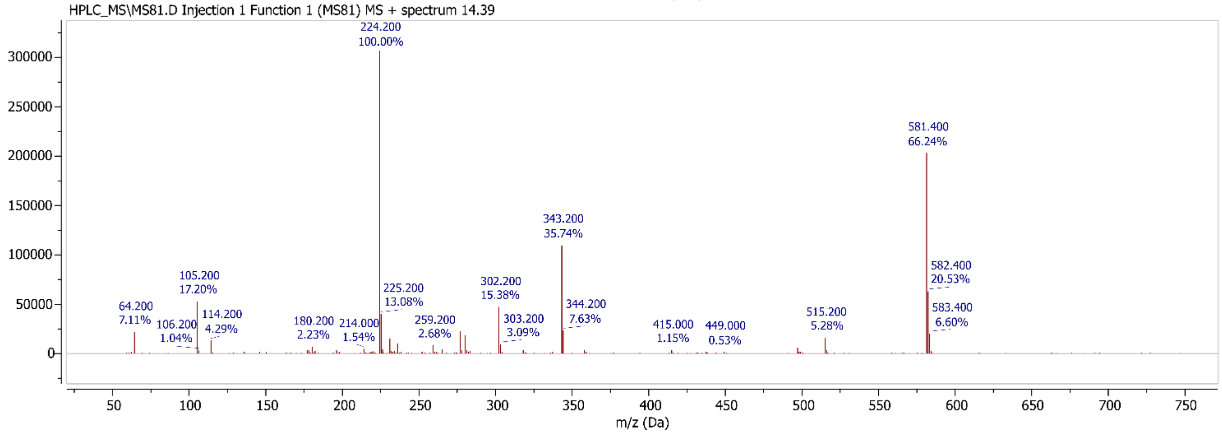
HPLC_MS\MS81.D Injection 1 Function 1 (MS81) MS + spectrum 14.39
224.200
100.00%
581.400
66.24%
343.200
35.74%
582.400
20.53%
105.200
17.20%
302.200
15.38%
225.200
13.08%
64.200
7.11%
344.200
7.63%
583.400
6.60%
515.200
5.28%
114.200
4.29%
303.200
3.09%
259.200
2.68%
180.200
2.23%
214.000
1.54%
106.200
1.04%
415.000
1.15%
449.000
0.53%
m/z (Da)

***tert*-butyl (*R*)-2-(1-vinylcycloheptyl)pyrrolidine-1-carboxylate (*R*-9d)**

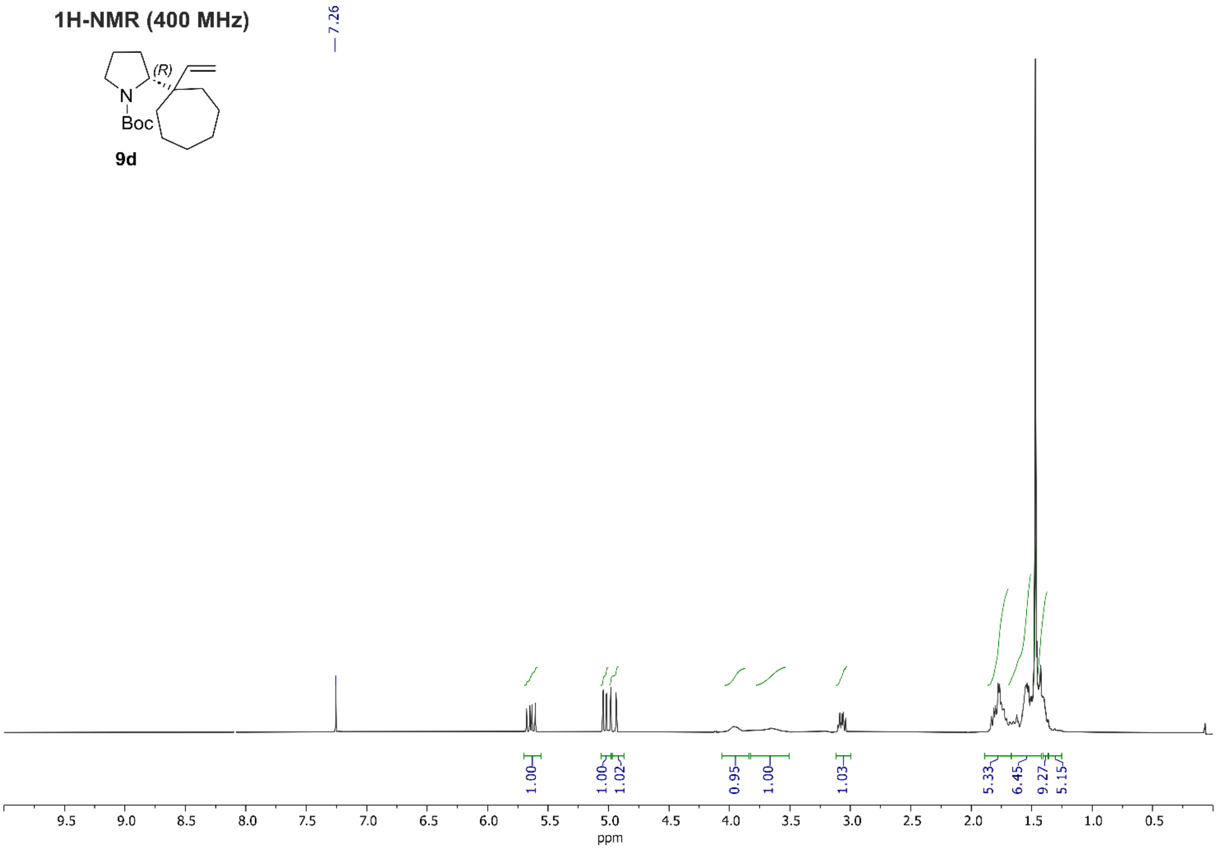

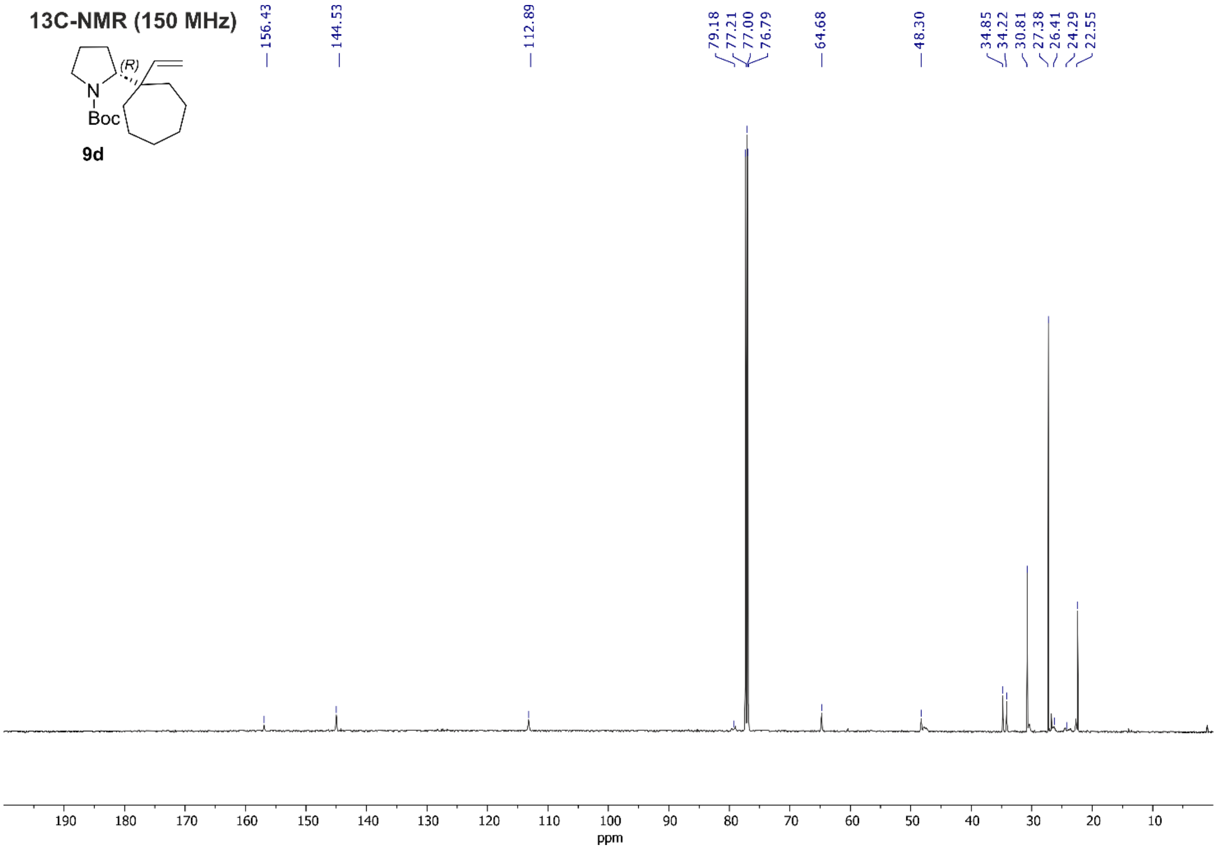

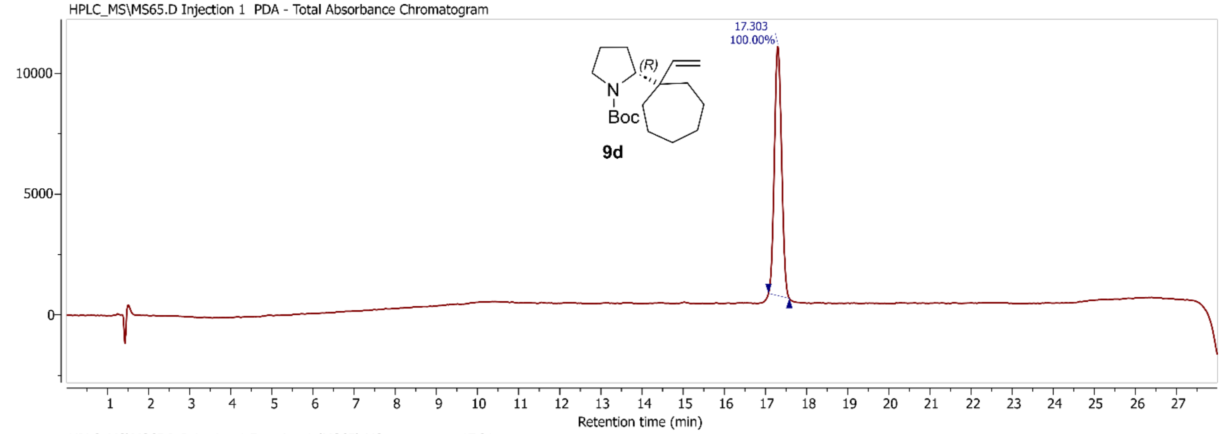
HPLC_MS\MS65.D Injection 1 PDA - Total Absorbance Chromatogram
17.303
100.00%
(R)
N
Boc
9d
Retention time (min)

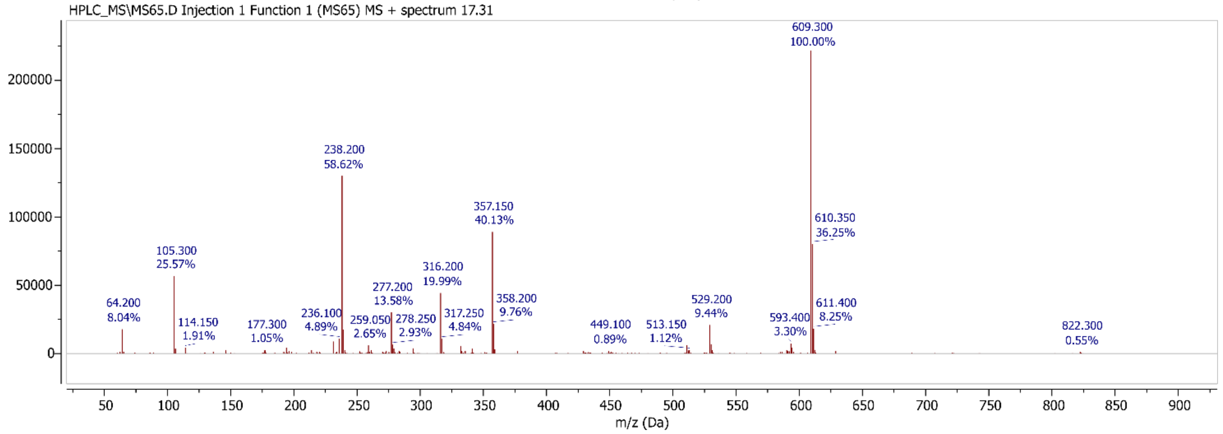
HPLC_MS\MS65.D Injection 1 Function 1 (MS65) MS + spectrum 17.31
609.300
100.00%
238.200
58.62%
357.150
40.13%
610.350
36.25%
105.300
25.57%
316.200
19.99%
277.200
13.58%
358.200
9.76%
529.200
9.44%
611.400
8.25%
64.200
8.04%
236.100
4.89%
317.250
4.84%
593.400
3.30%
278.250
2.93%
259.050
2.65%
114.150
1.91%
513.150
1.12%
177.300
1.05%
449.100
0.89%
822.300
0.55%
m/z (Da)

***tert*-butyl (*R*)-2-(2-methyl-1-oxopropan-2-yl)pyrrolidine-1-carboxylate (*R*-10a)**

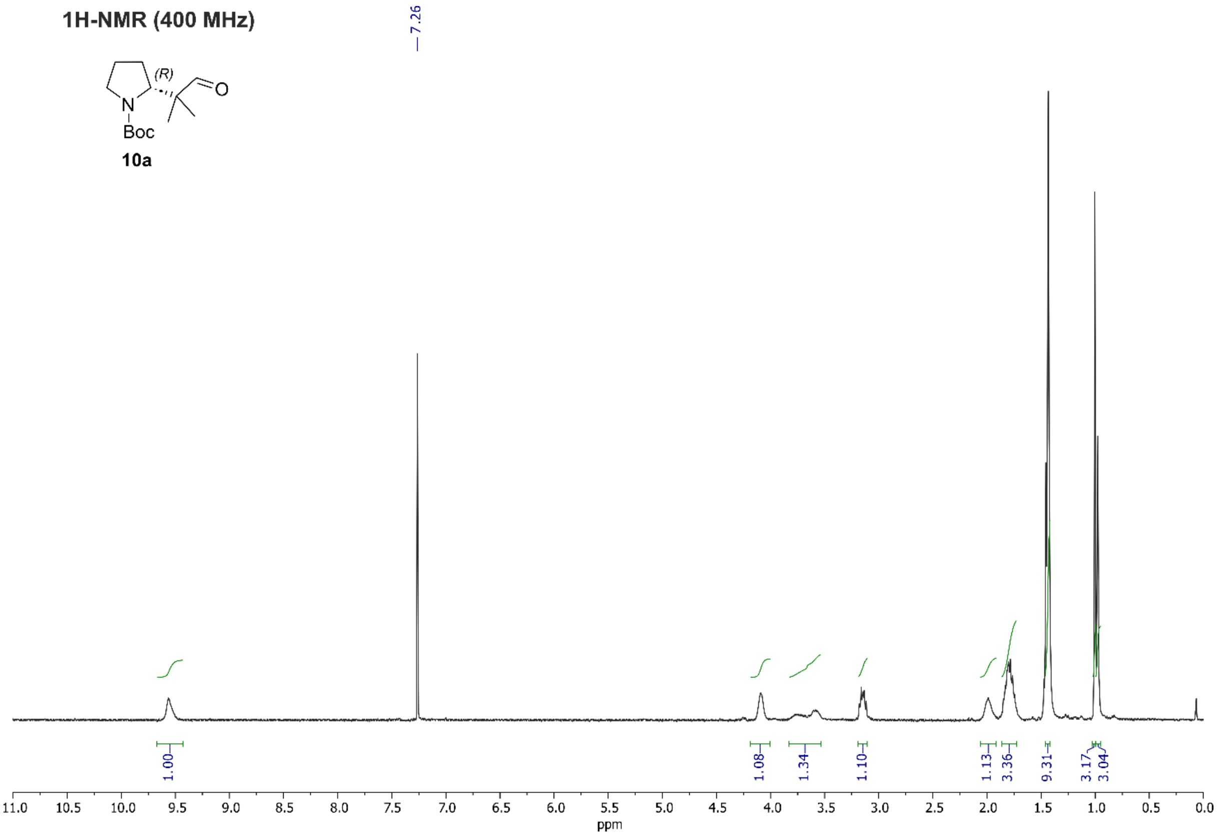

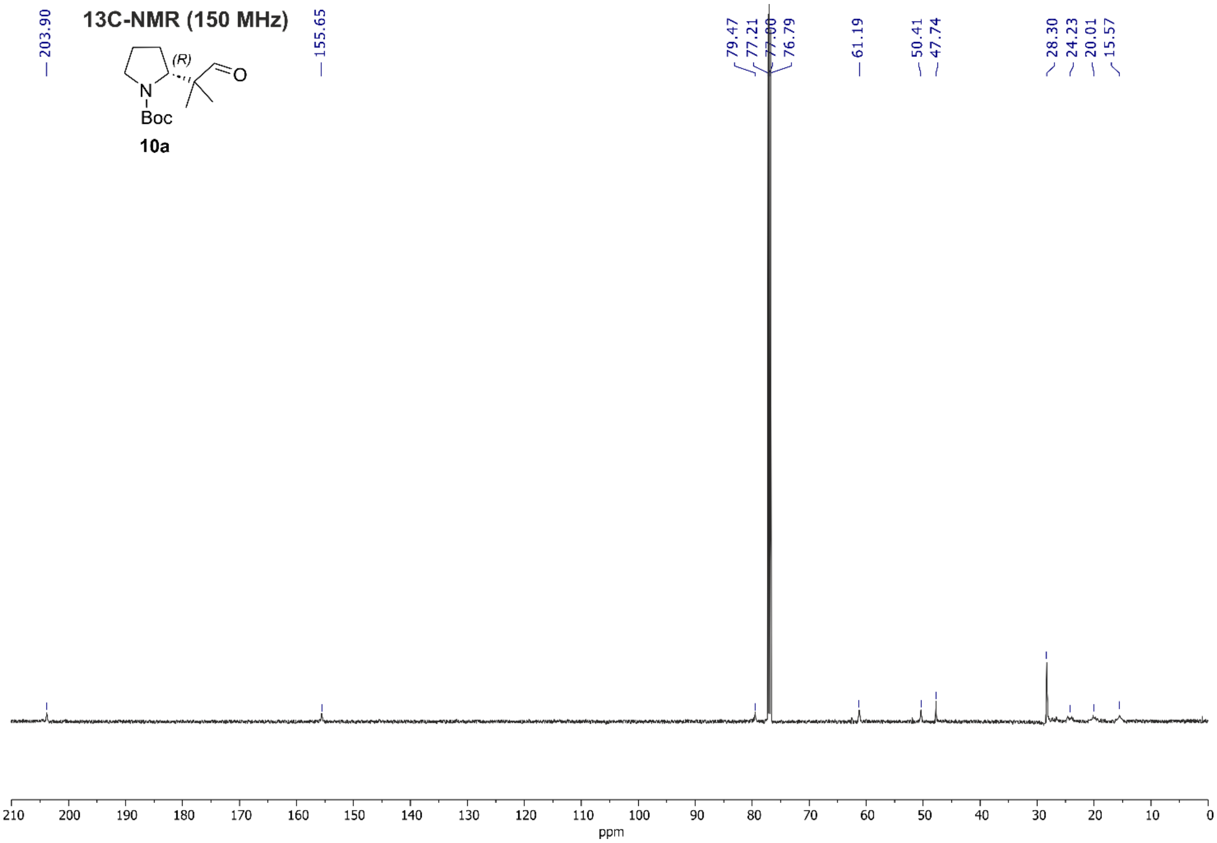

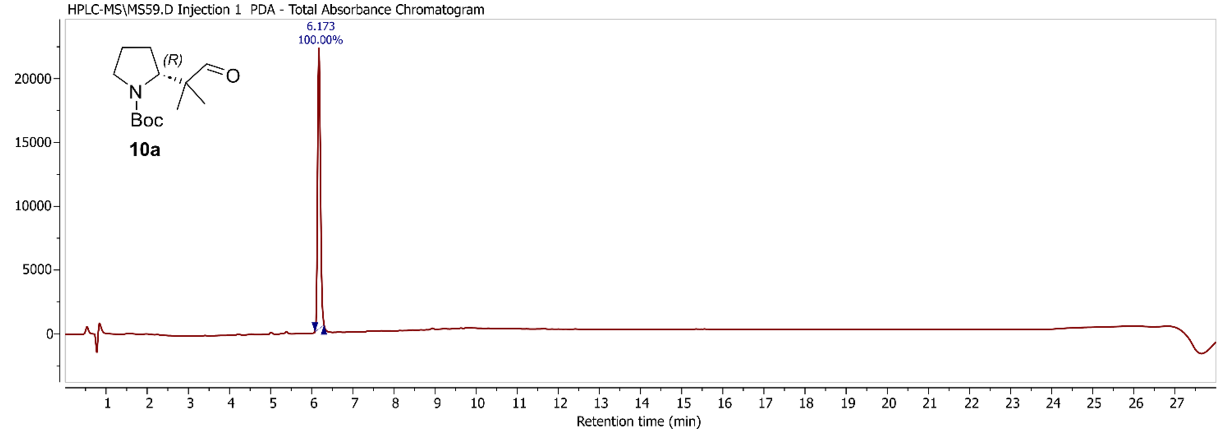
HPLC-MS\MS59.D Injection 1 PDA - Total Absorbance Chromatogram
6.173
100.00%
(R)
N
O
Boc
10a
Retention time (min)

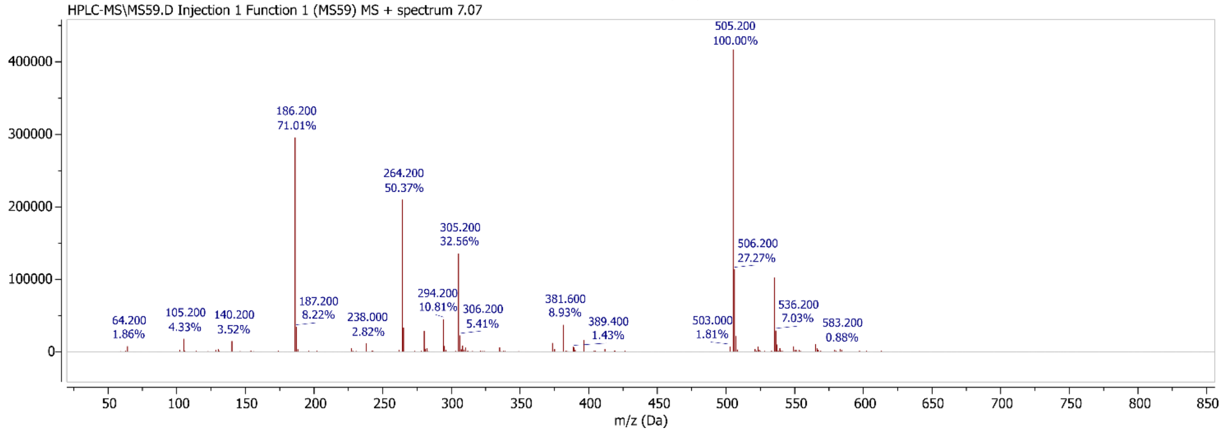
HPLC-MS\MS59.D Injection 1 Function 1 (MS59) MS + spectrum 7.07
505.200
100.00%
186.200
71.01%
264.200
50.37%
305.200
32.56%
506.200
27.27%
294.200
10.81%
381.600
8.93%
187.200
8.22%
536.200
7.03%
306.200
5.41%
105.200
4.33%
140.200
3.52%
238.000
2.82%
64.200
1.86%
503.000
1.81%
389.400
1.43%
583.200
0.88%
m/z (Da)

***tert*-butyl (*R*)-2-(1-formylcyclopentyl)pyrrolidine-1-carboxylate (*R*-10b)**

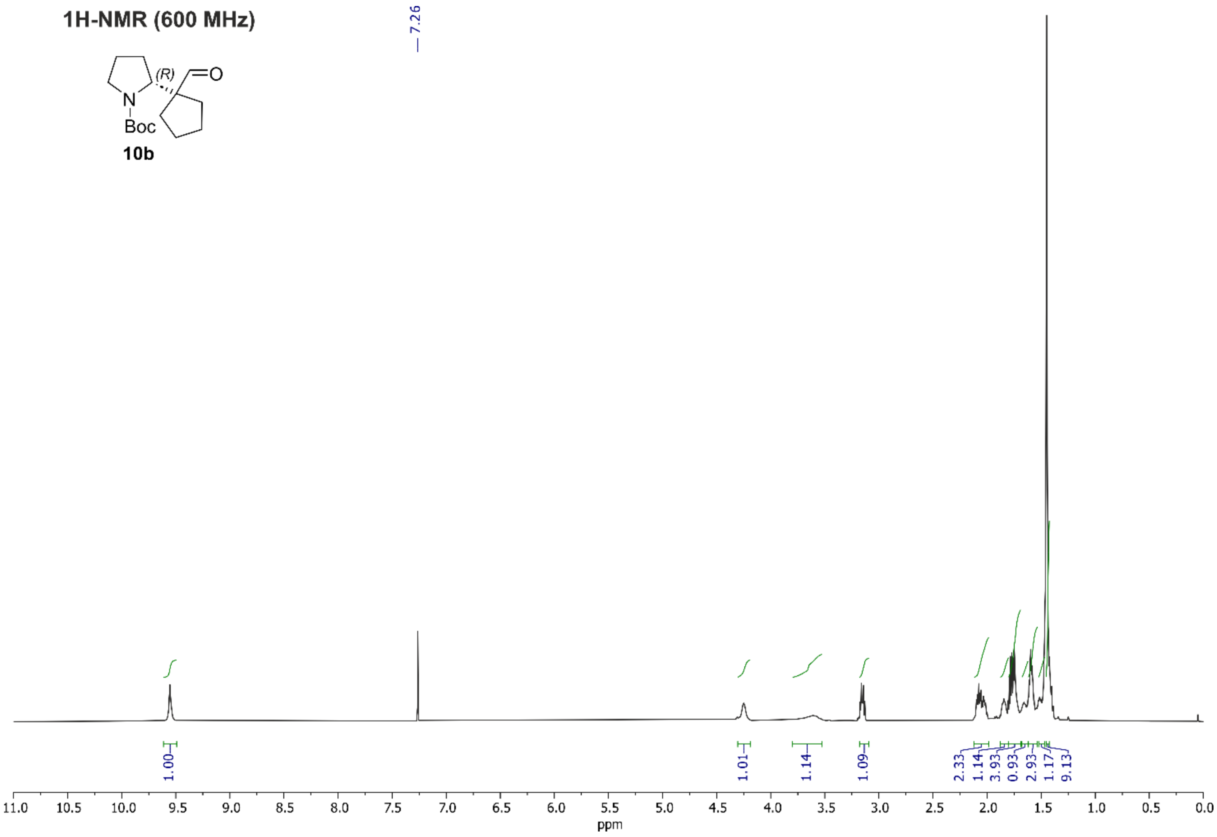

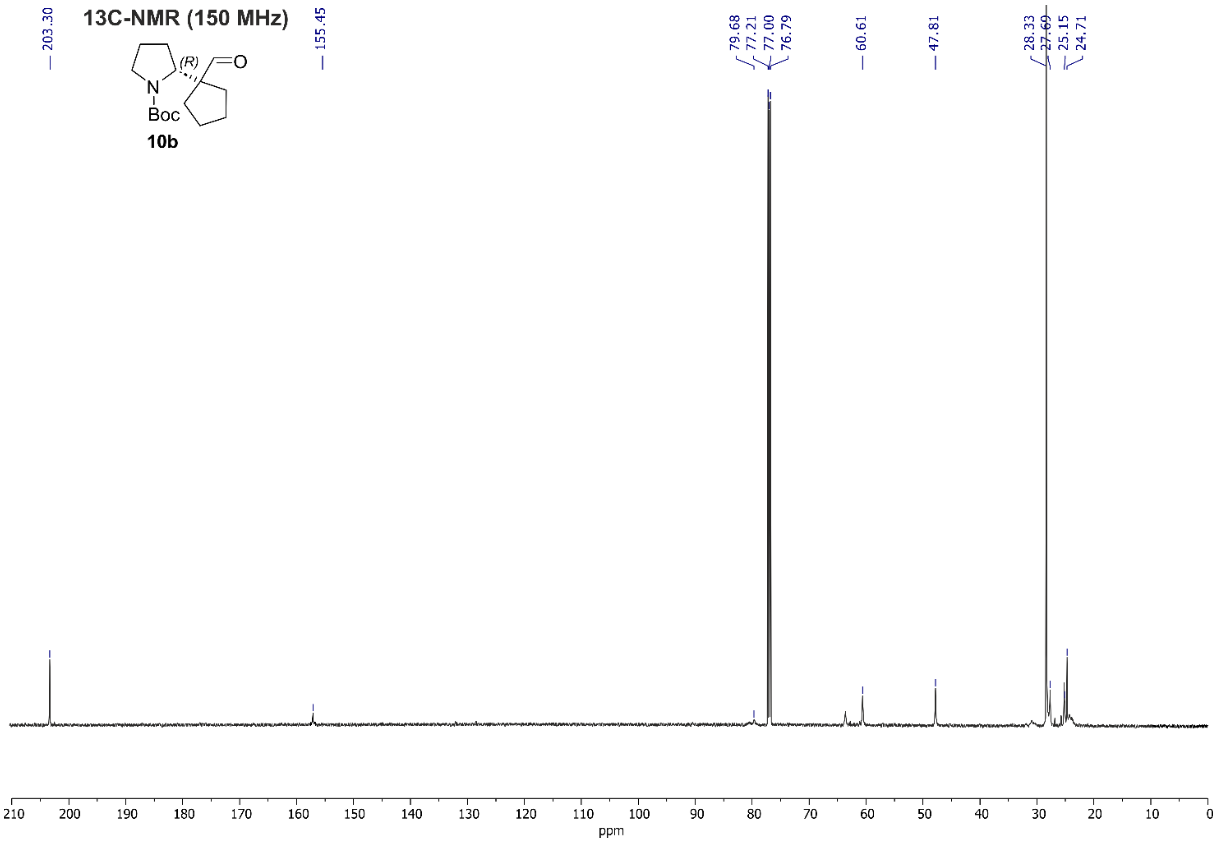

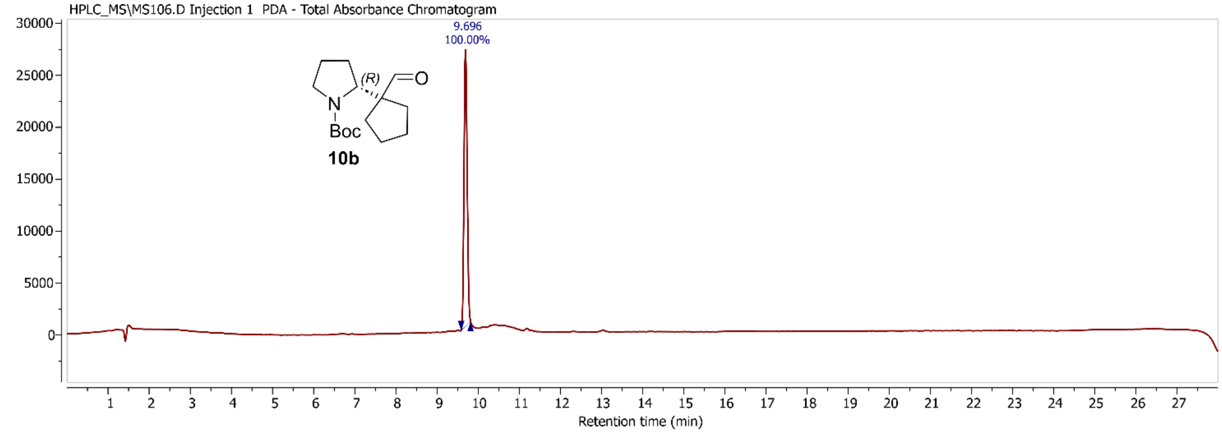
HPLC_MS\MS106.D Injection 1 PDA - Total Absorbance Chromatogram
9.696
100.00%
(R)
N
Boc
O
10b
Retention time (min)

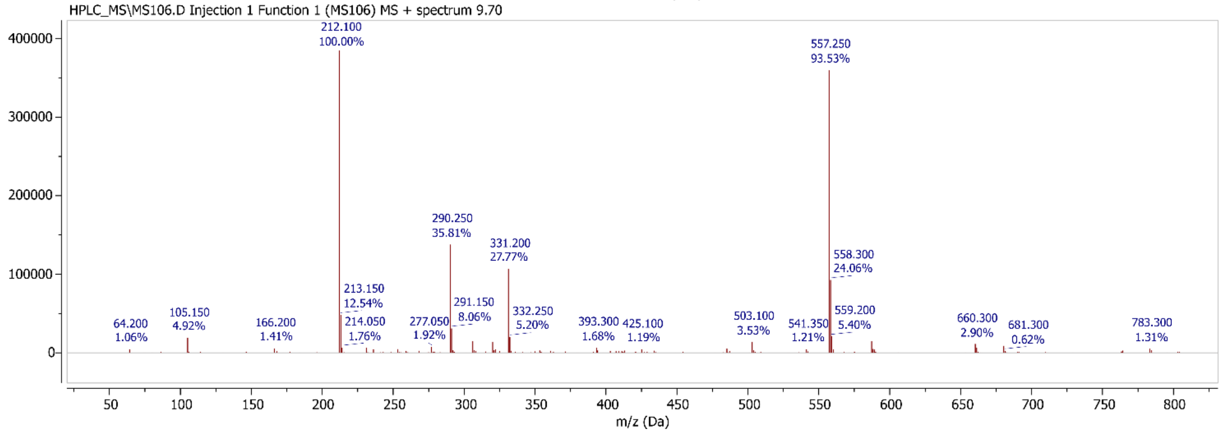
HPLC_MS\MS106.D Injection 1 Function 1 (MS106) MS + spectrum 9.70
212.100
100.00%
557.250
93.53%
290.250
35.81%
331.200
27.77%
558.300
24.06%
213.150
12.54%
105.150
4.92%
m/z (Da)

tert-butyl (*R*)-2-(1-formylcyclohexyl)pyrrolidine-1-carboxylate (*R*-10c)

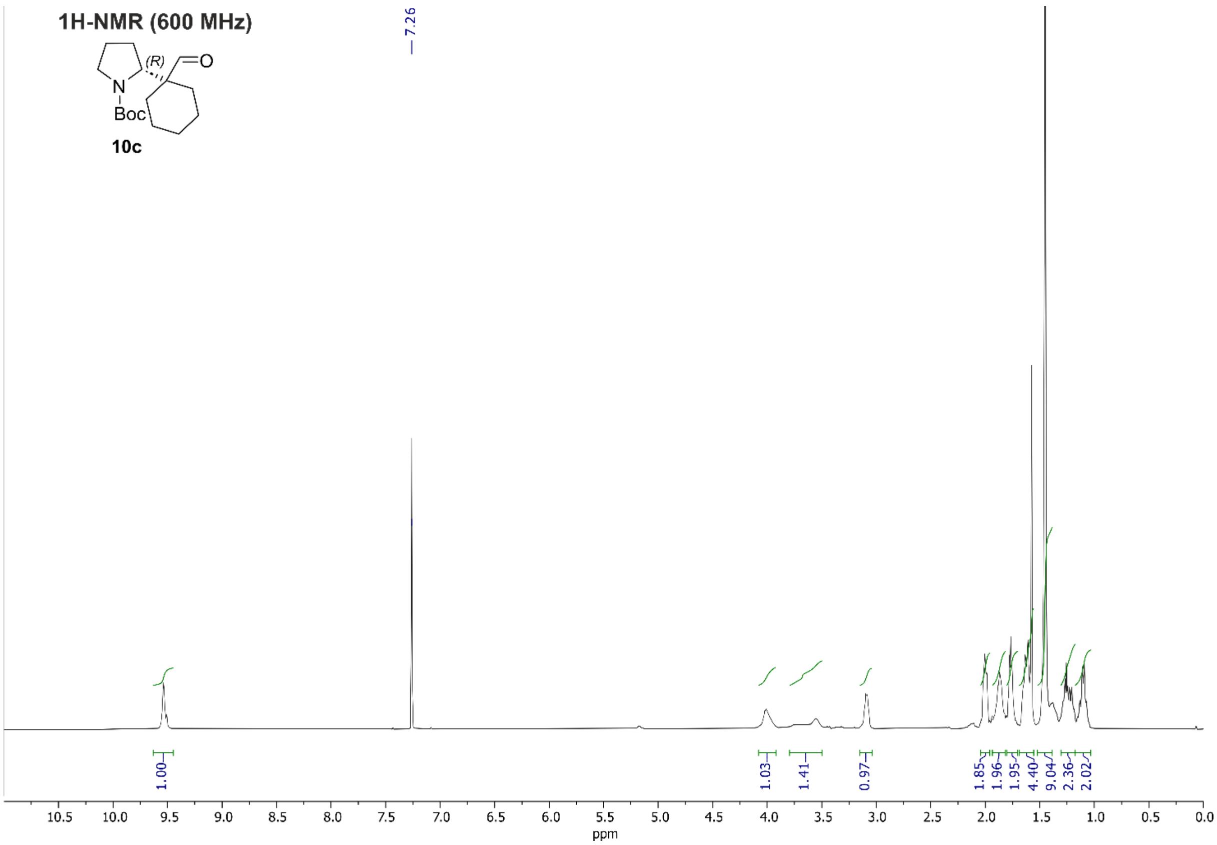

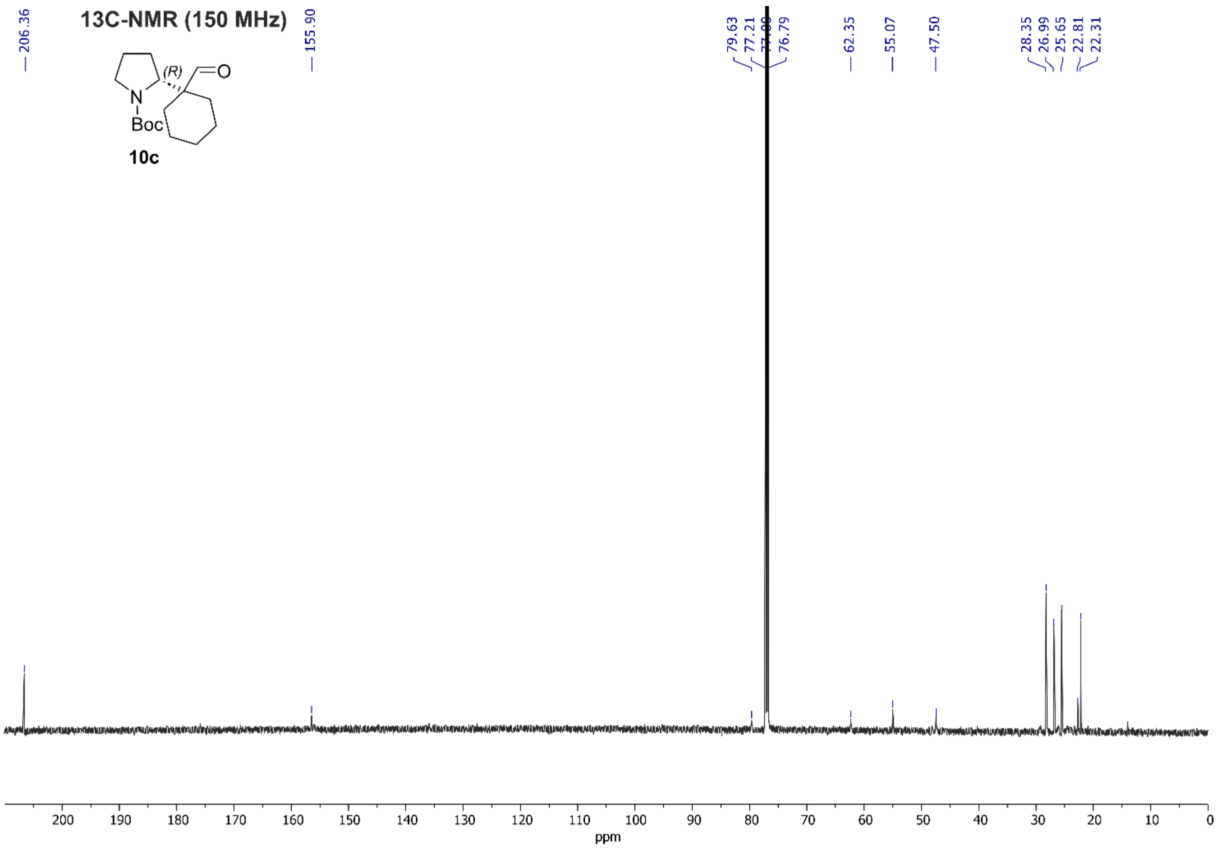

HPLC_MS\MS102.D Injection 1 PDA - Total Absorbance Chromatogram

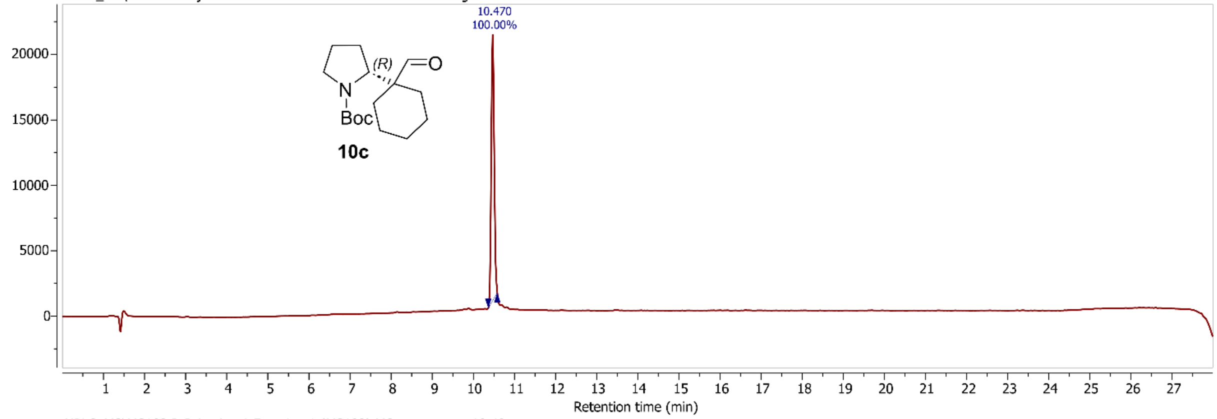

HPLC_MS\MS102.D Injection 1 Function 1 (MS102) MS + spectrum 10.48

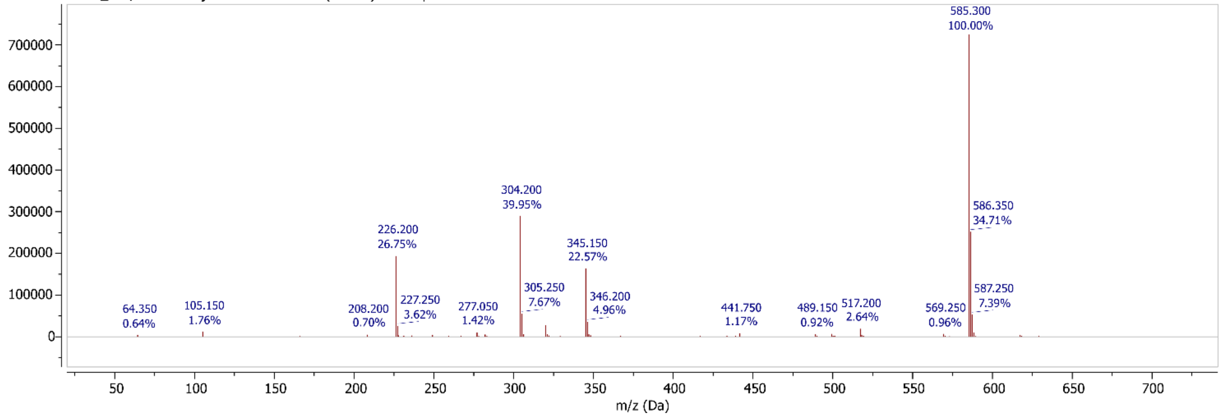

***tert*-butyl (*R*)-2-(1-formylcycloheptyl)pyrrolidine-1-carboxylate (*R*-10d)**

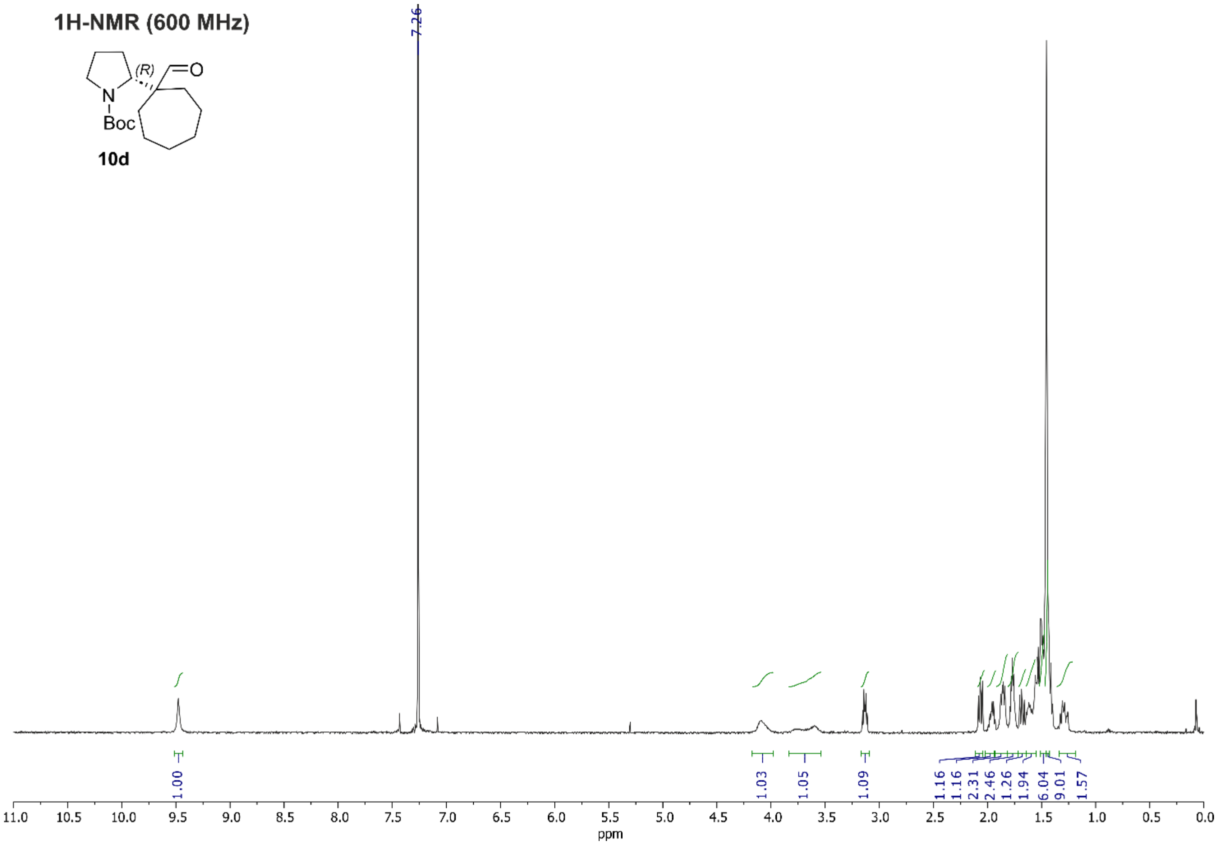

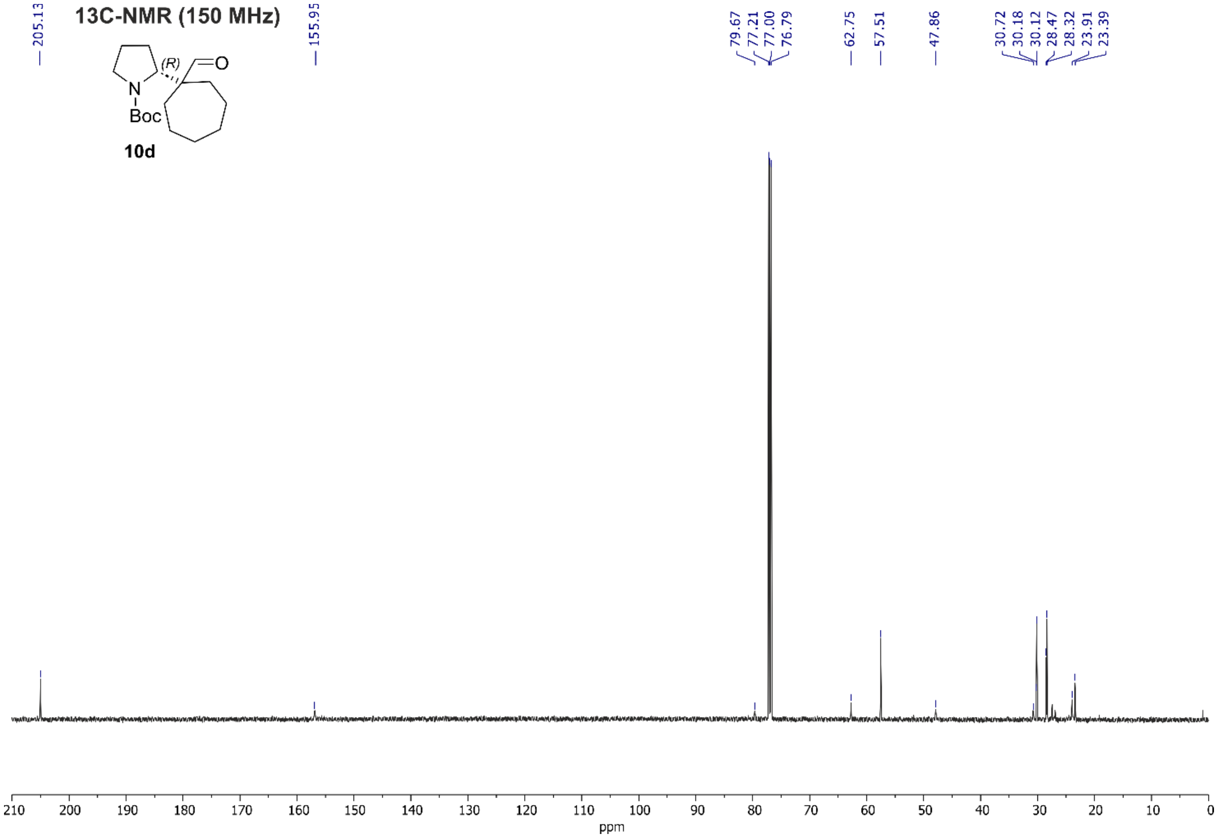

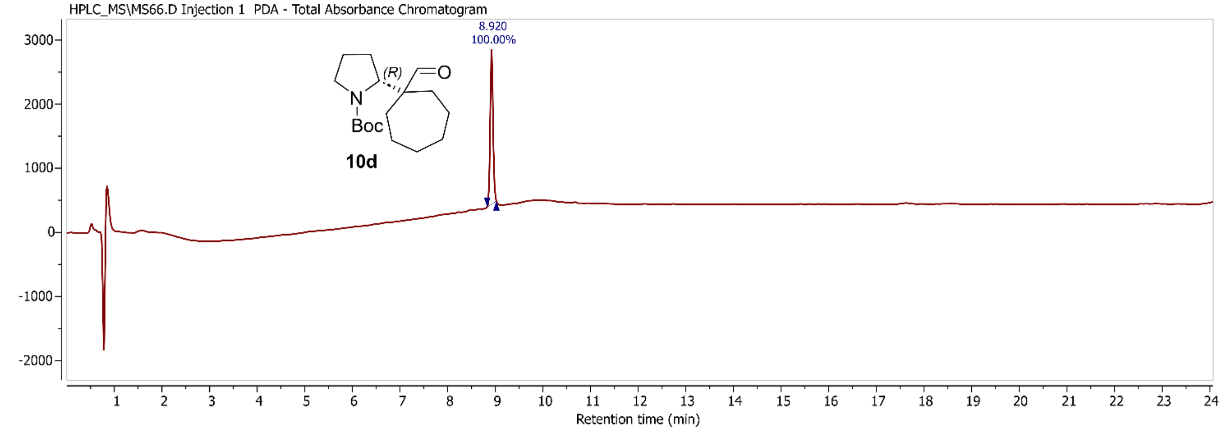

HPLC_MS\MS66.D Injection 1 PDA - Total Absorbance Chromatogram
8.920
100.00%
(R)
N
Boc
O
10d
Retention time (min)

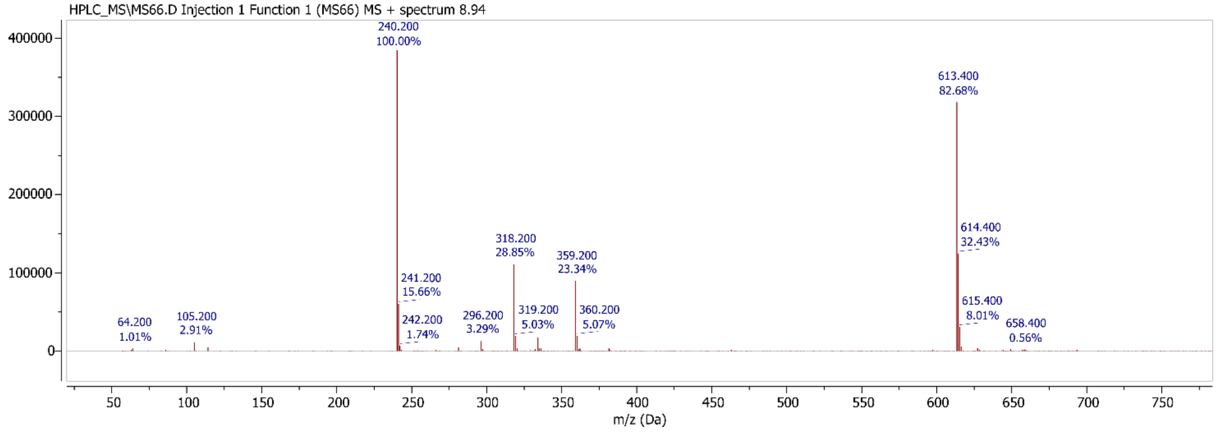

HPLC_MS\MS66.D Injection 1 Function 1 (MS66) MS + spectrum 8.94
240.200 100.00%
613.400 82.68%
614.400 32.43%
318.200 28.85%
359.200 23.34%
241.200 15.66%
615.400 8.01%
64.200 1.01%
105.200 2.91%
242.200 1.74%
296.200 3.29%
319.200 5.03%
360.200 5.07%
658.400 0.56%
m/z (Da)

(*R*)-2-(1-(*tert*-butoxycarbonyl)pyrrolidin-2-yl)-2-methylpropanoic acid (*R*-11a)

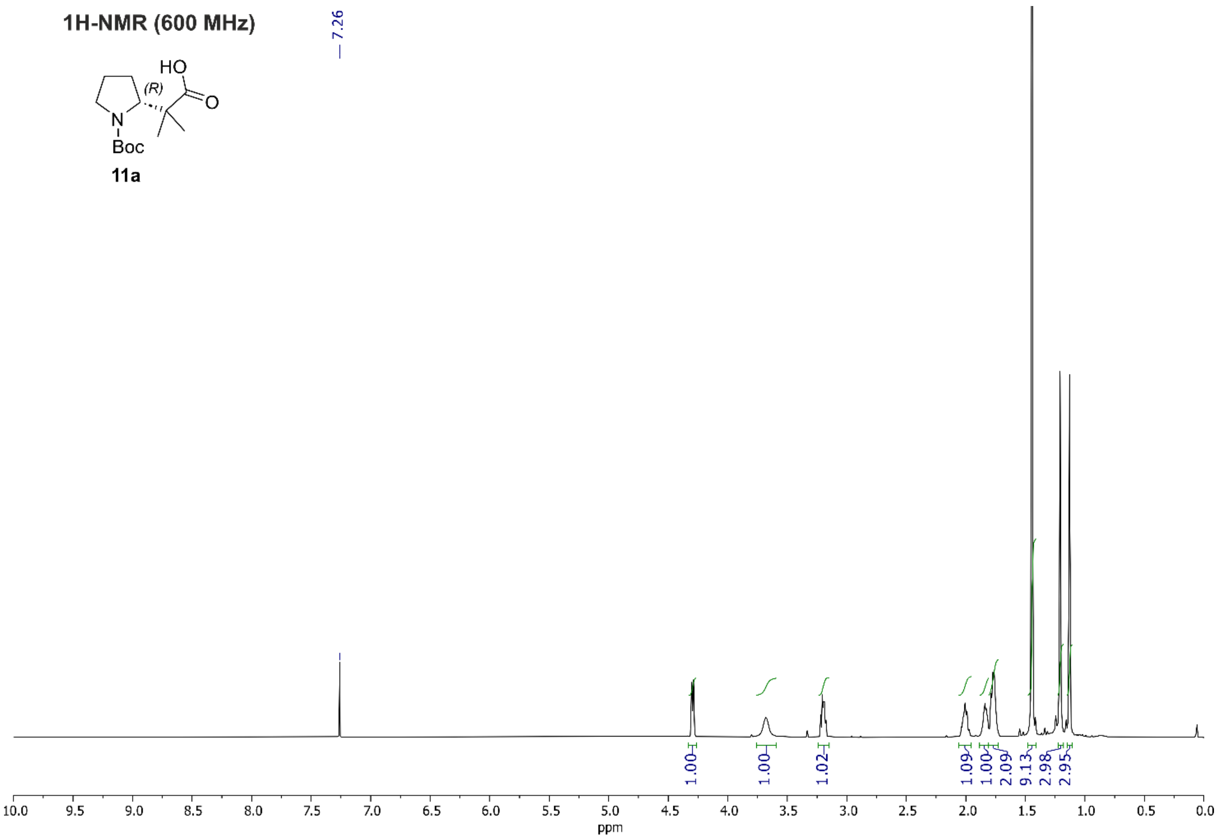

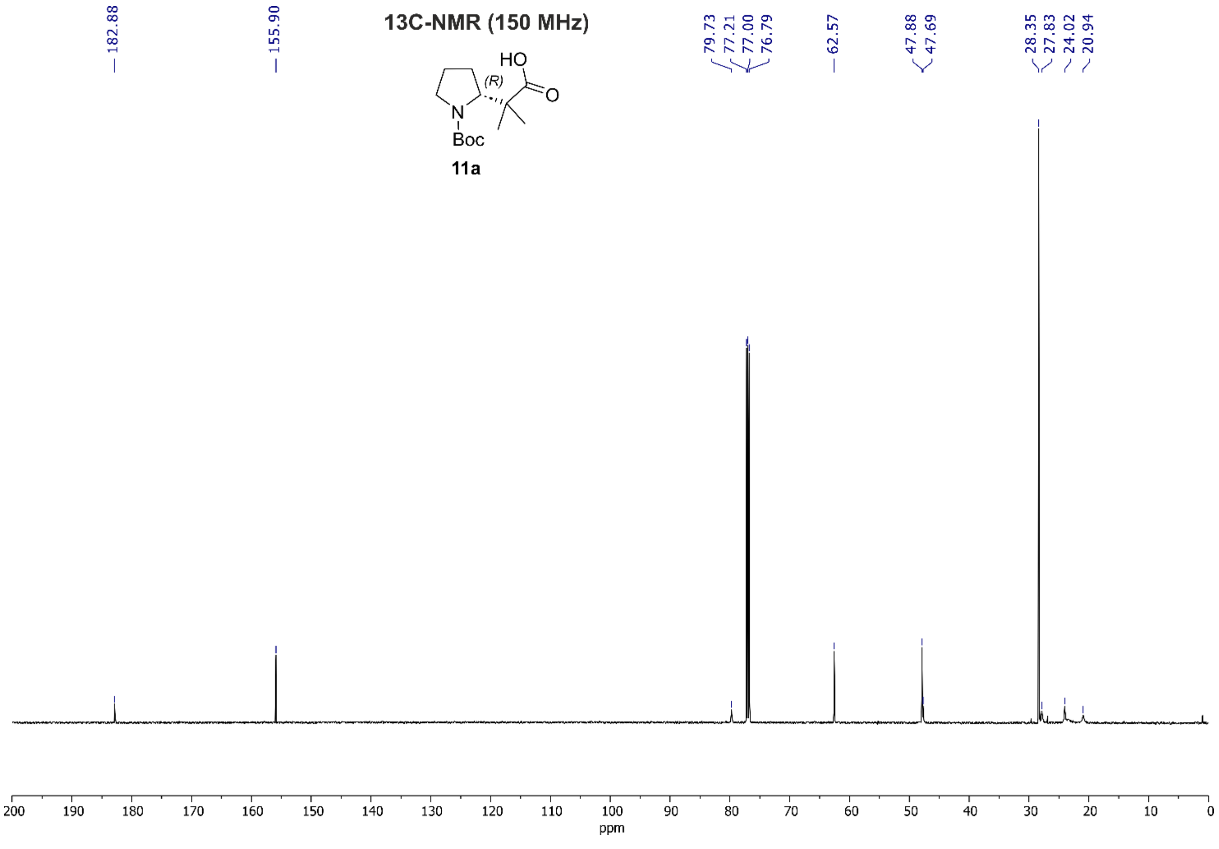

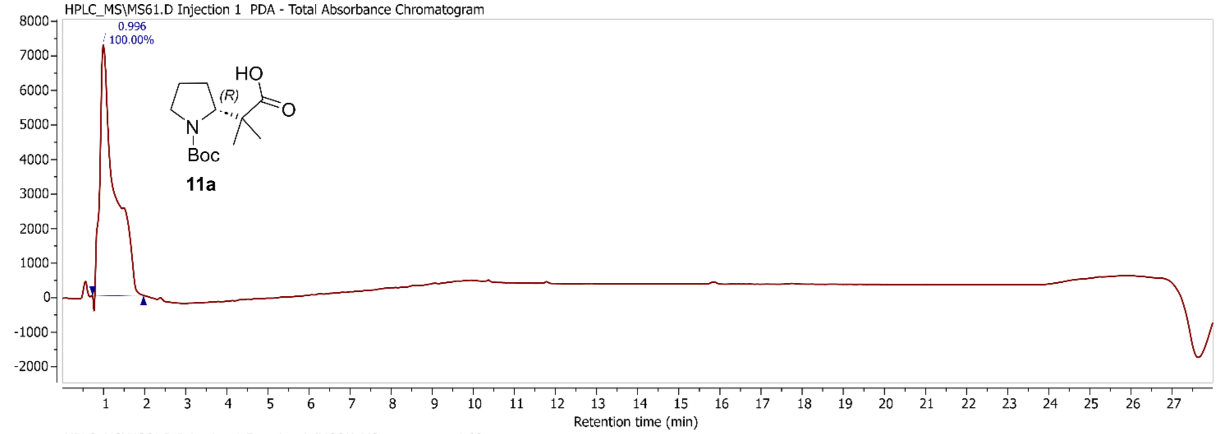

HPLC_MS\MS61.D Injection 1 PDA - Total Absorbance Chromatogram
0.996
100.00%
HO
(R)
O
N
Boc
11a
Retention time (min)

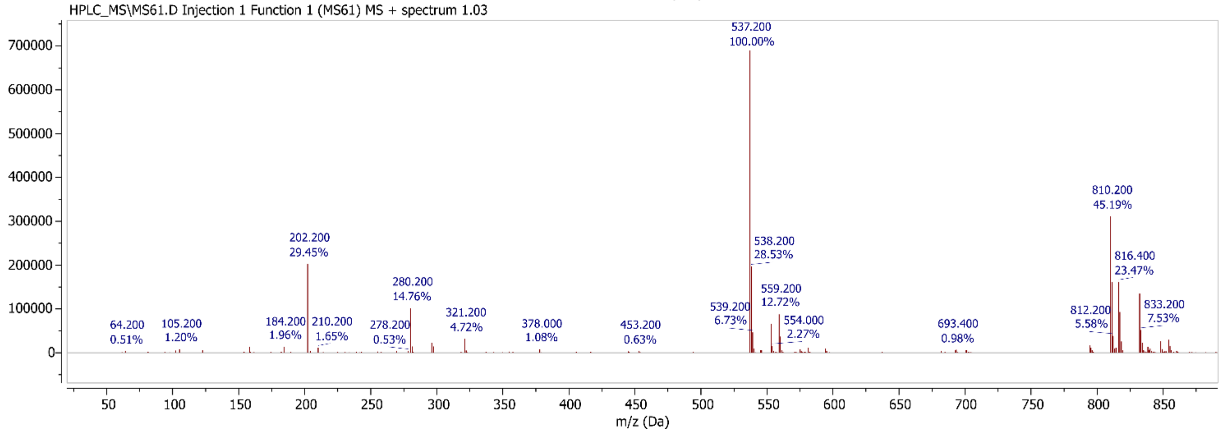

HPLC_MS\MS61.D Injection 1 Function 1 (MS61) MS + spectrum 1.03
537.200
100.00%
810.200
45.19%
202.200
29.45%
538.200
28.53%
816.400
23.47%
280.200
14.76%
559.200
12.72%
539.200
6.73%
812.200
5.58%
833.200
7.53%
321.200
4.72%
554.000
2.27%
184.200
1.96%
210.200
1.65%
105.200
1.20%
378.000
1.08%
693.400
0.98%
453.200
0.63%
278.200
0.53%
64.200
0.51%
m/z (Da)

(*R*)-2-(1-(*tert*-butoxycarbonyl)pyrrolidin-2-yl)cyclopentane-1-carboxylic acid (*R*-11b)

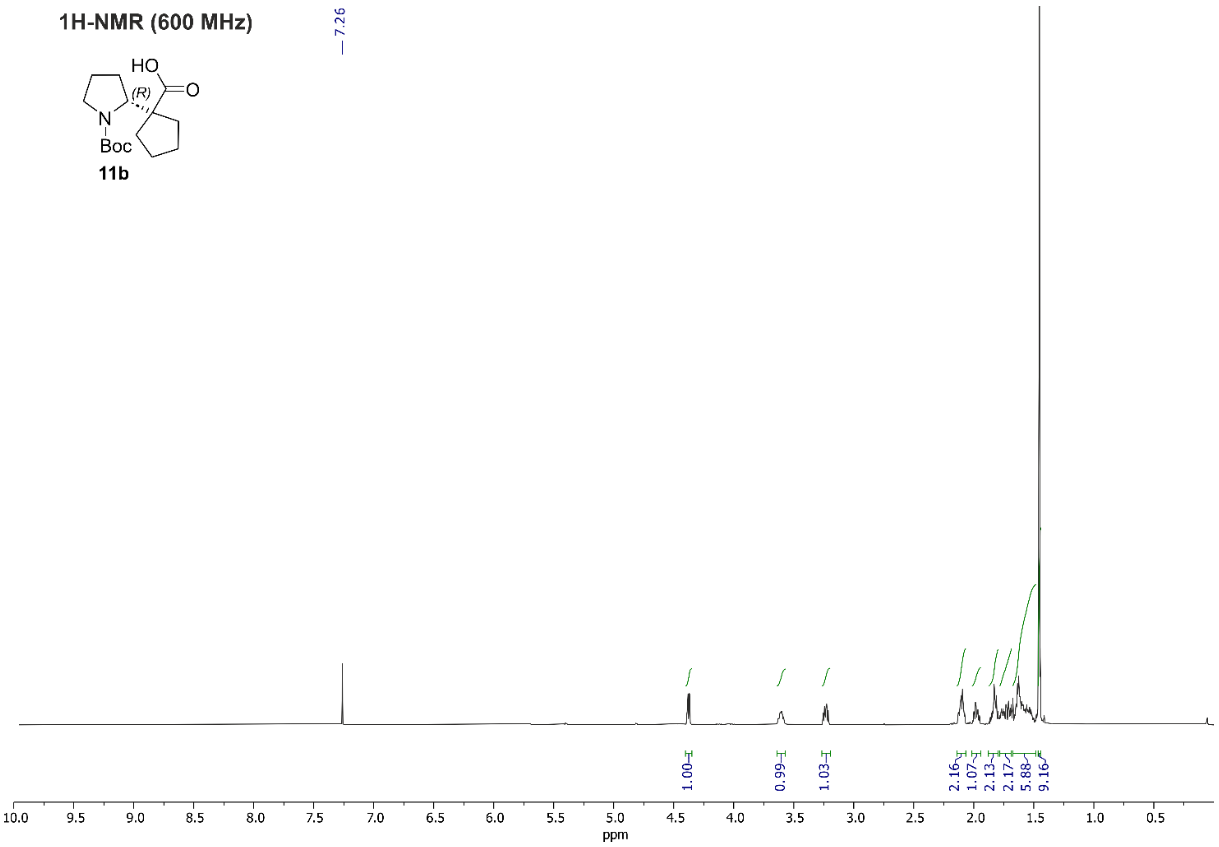

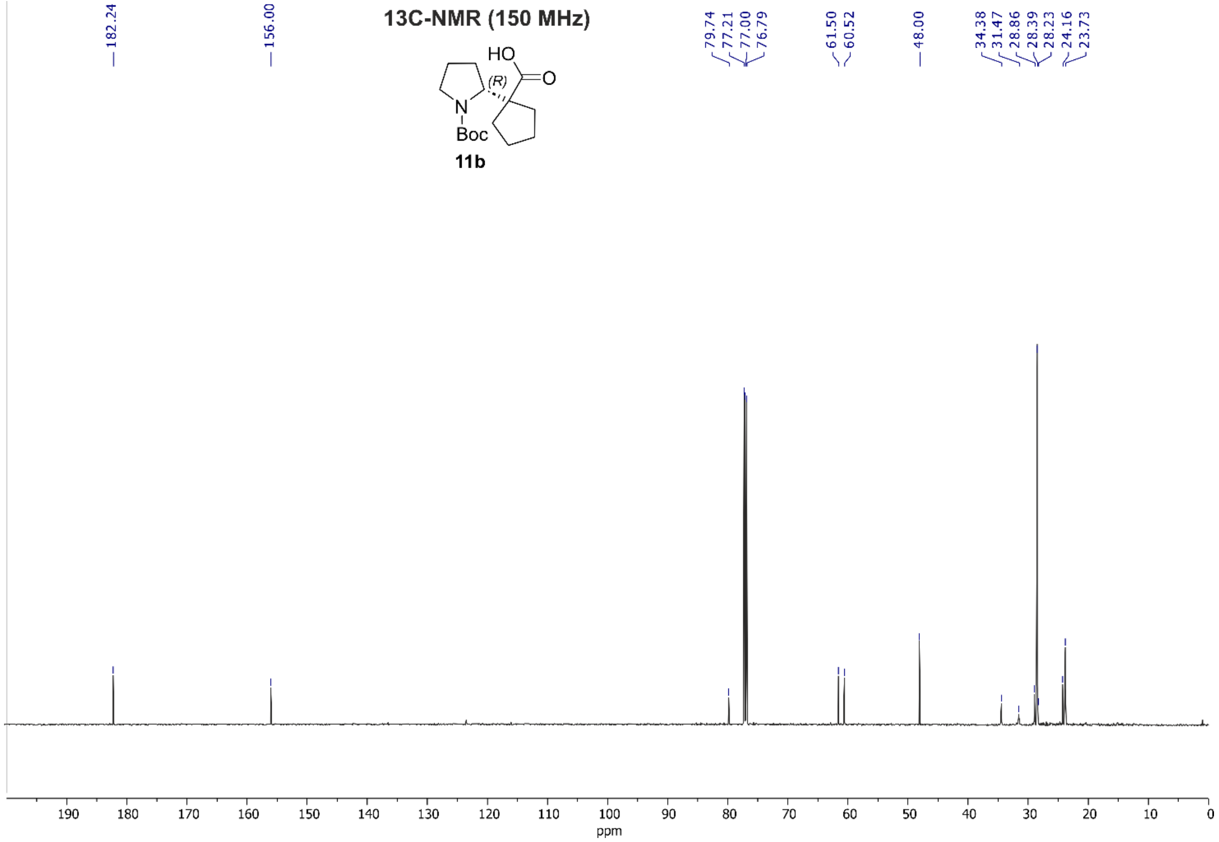

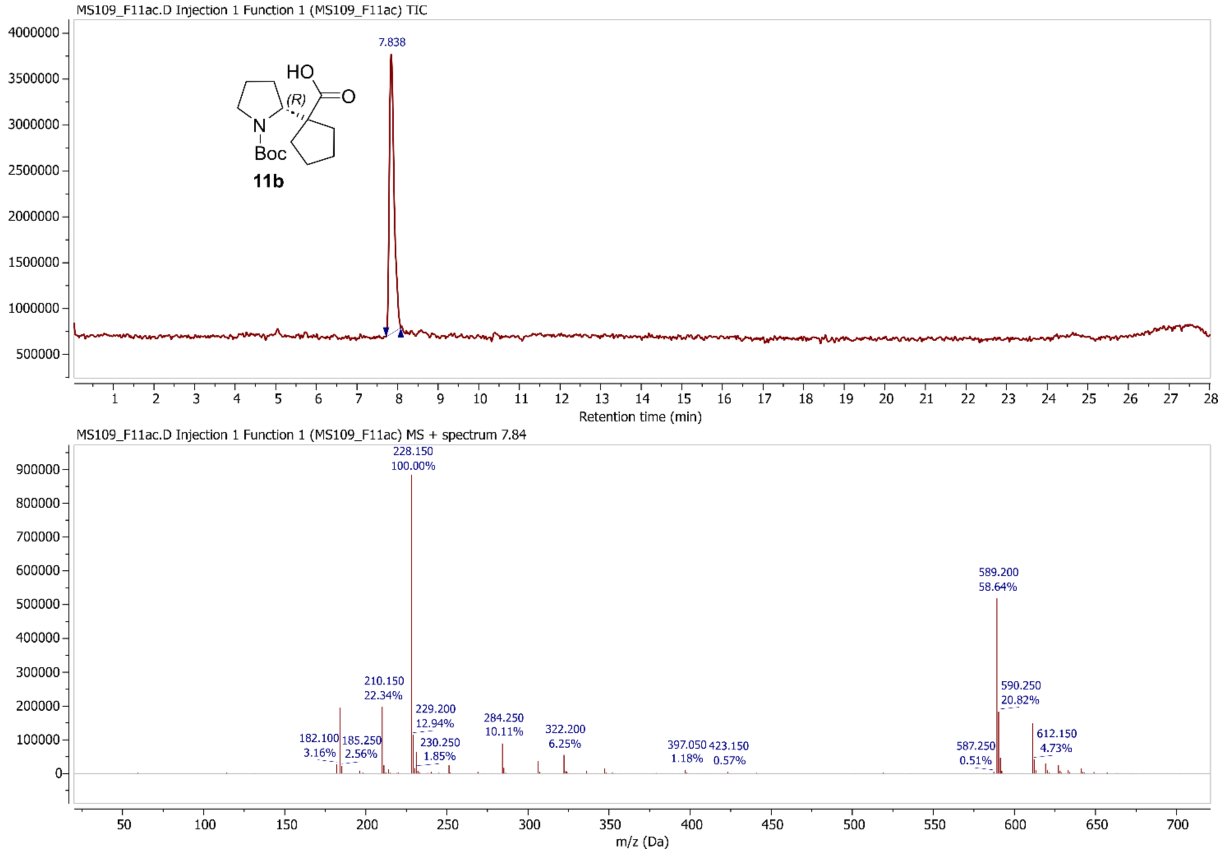
MS109_F11ac.D Injection 1 Function 1 (MS109_F11ac) TIC
7.838
HO
(R)
O
N
Boc
11b
Retention time (min)
MS109_F11ac.D Injection 1 Function 1 (MS109_F11ac) MS + spectrum 7.84
228.150
100.00%
589.200
58.64%
210.150
22.34%
590.250
20.82%
229.200
12.94%
284.250
10.11%
322.200
6.25%
182.100
3.16%
185.250
2.56%
230.250
1.85%
397.050
1.18%
423.150
0.57%
587.250
0.51%
612.150
4.73%
m/z (Da)

(*R*)-2-(1-(*tert*-butoxycarbonyl)pyrrolidin-2-yl)cyclohexane-1-carboxylic acid (*R*-11c)

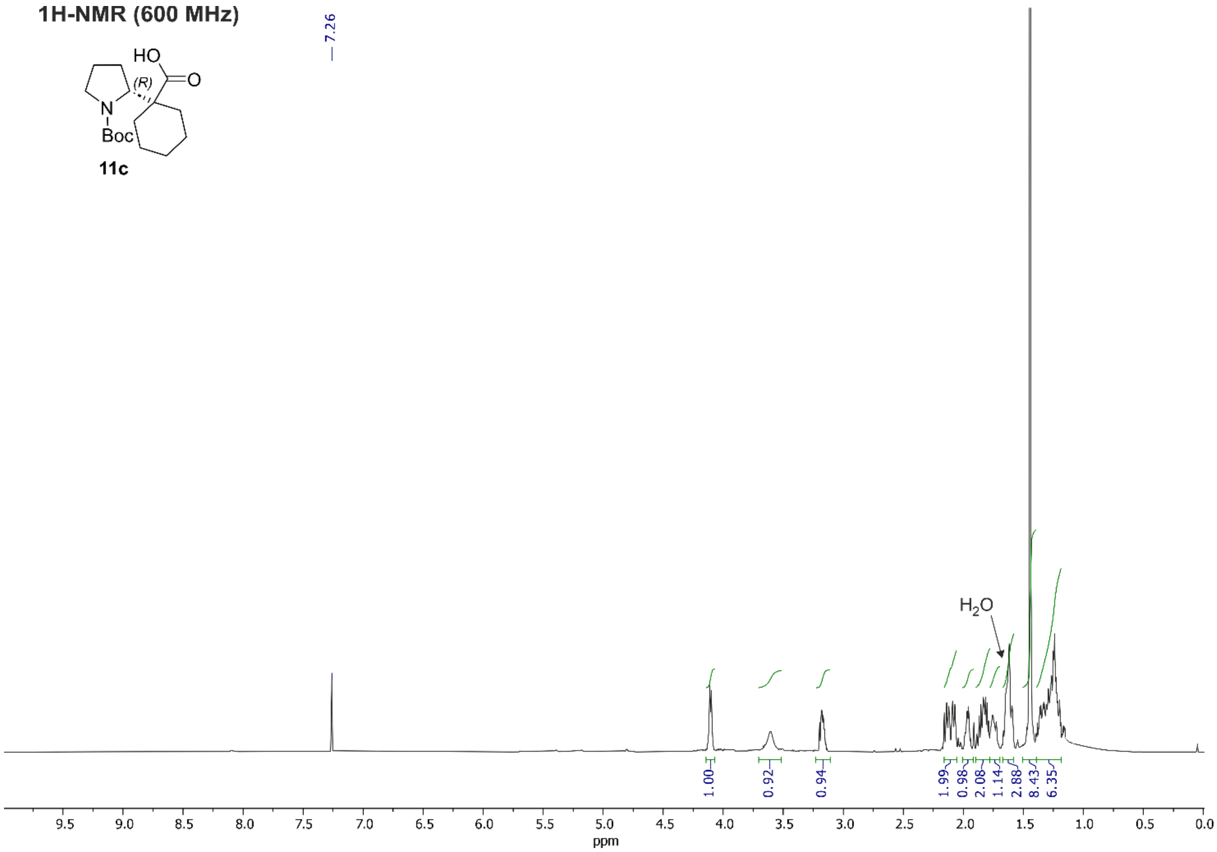

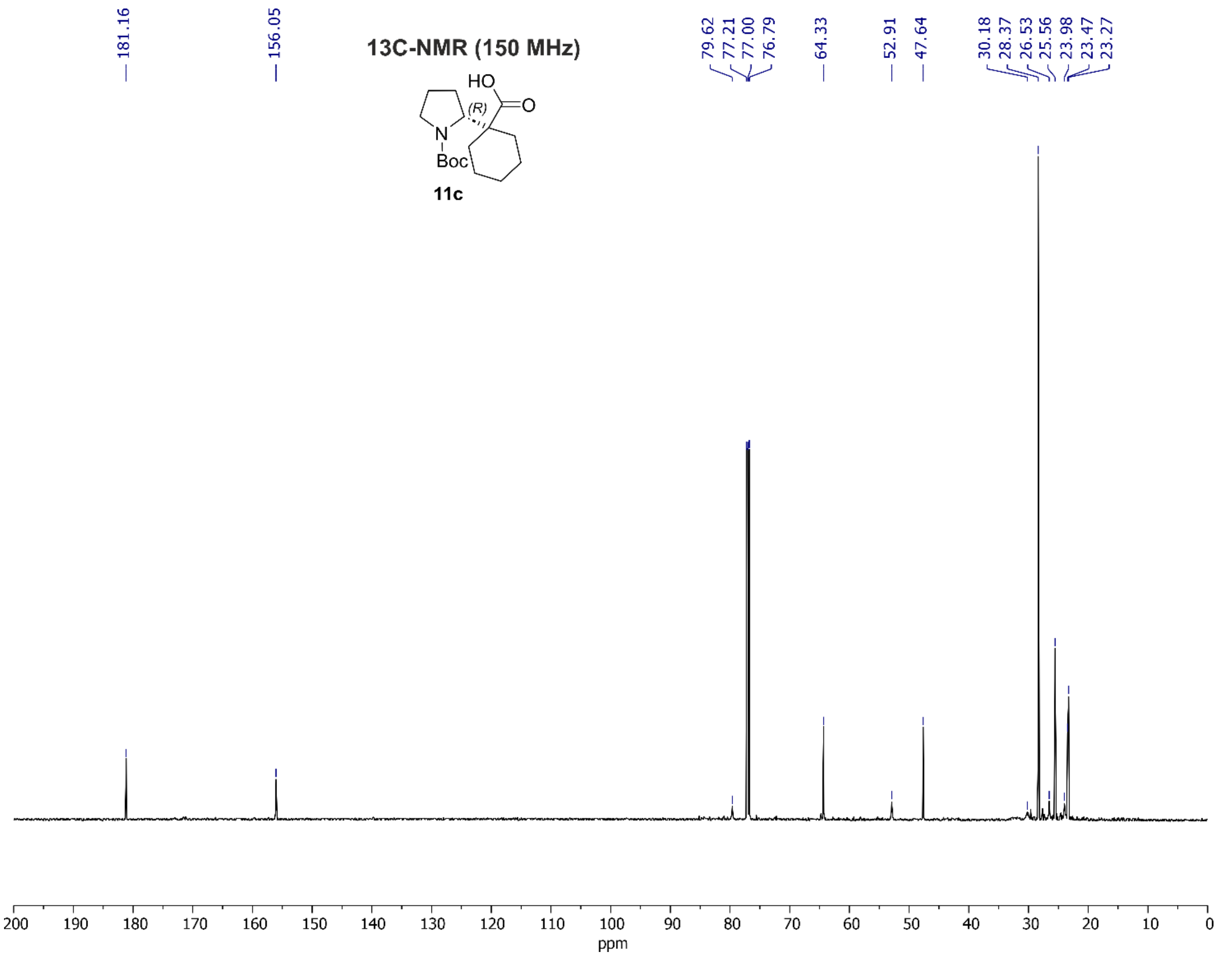

181.16
156.05
13C-NMR (150 MHz)
HO
O
(R)
N
Boc
11c
79.62
77.21
77.00
76.79
64.33
52.91
47.64
30.18
28.37
26.53
25.56
23.98
23.47
23.27
200 190 180 170 160 150 140 130 120 110 100 90 80 70 60 50 40 30 20 10 0
ppm

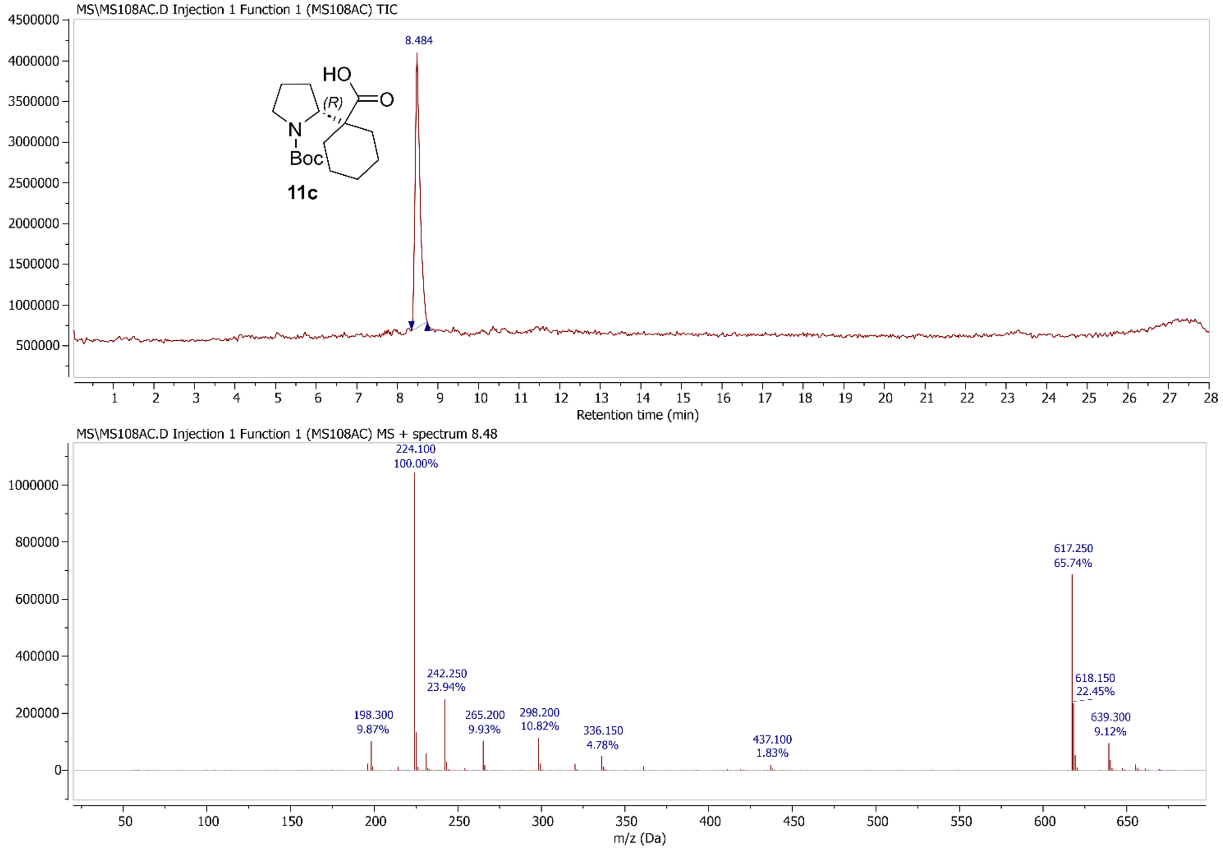
MS\MS108AC.D Injection 1 Function 1 (MS108AC) TIC
8.484
HO
O
(R)
N
Boc
11c
Retention time (min)
MS\MS108AC.D Injection 1 Function 1 (MS108AC) MS + spectrum 8.48
224.100
100.00%
617.250
65.74%
242.250
23.94%
618.150
22.45%
198.300
9.87%
265.200
9.93%
298.200
10.82%
336.150
4.78%
437.100
1.83%
639.300
9.12%
m/z (Da)

(*R*)-2-(1-(*tert*-butoxycarbonyl)pyrrolidin-2-yl)cycloheptane-1-carboxylic acid (*R*-11d)

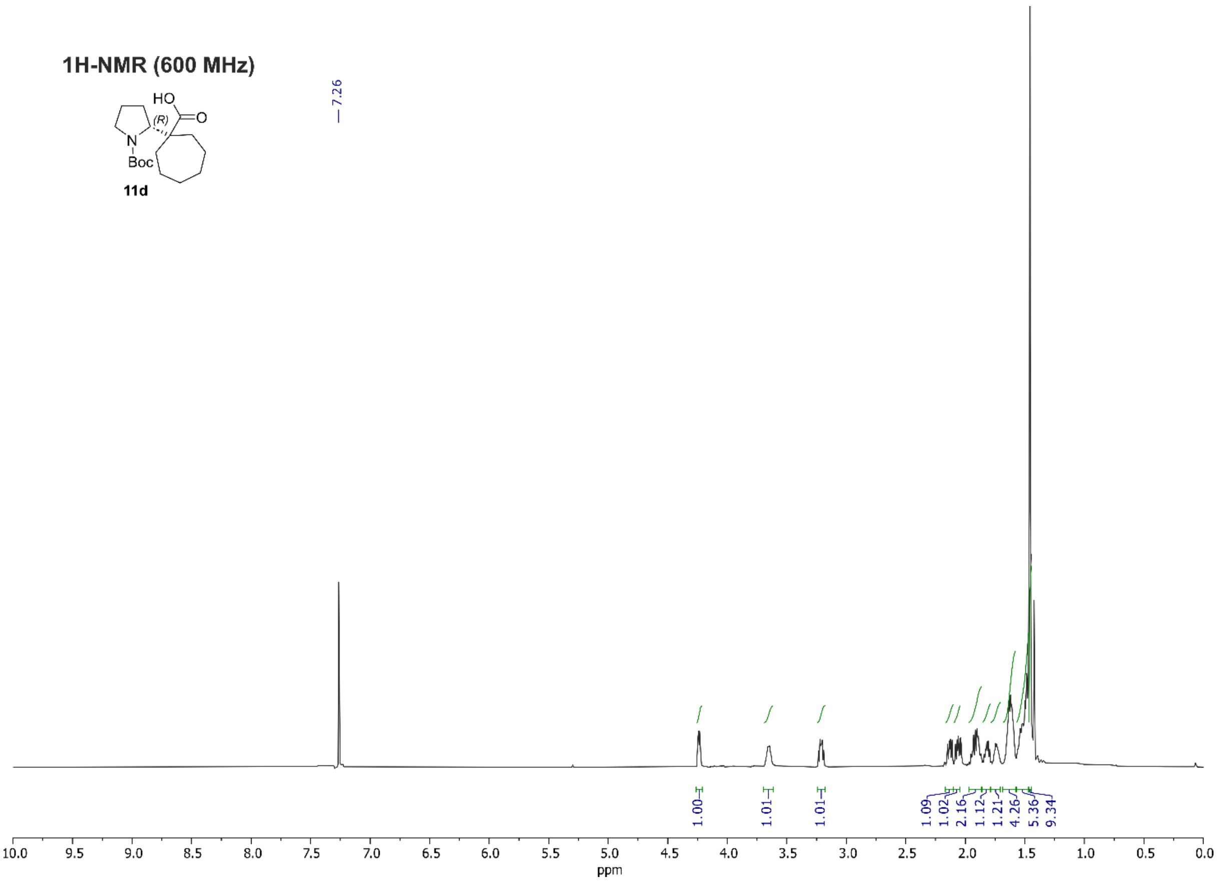

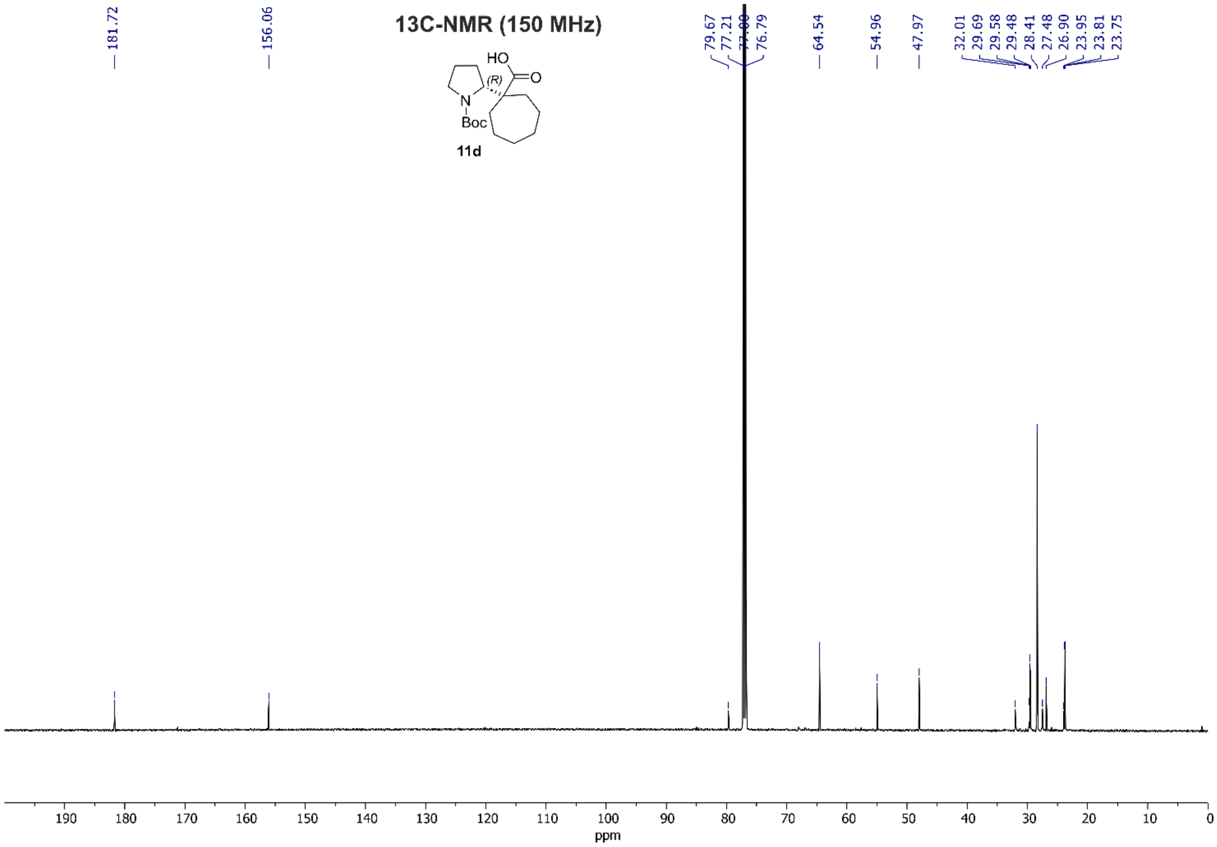
13C-NMR (150 MHz)
181.72
156.06
79.67
77.21
77.00
76.79
64.54
54.96
47.97
32.01
29.69
29.58
29.48
28.41
27.48
26.90
23.95
23.81
23.75
HO
O
(R)
N
Boc
11d
190 180 170 160 150 140 130 120 110 100 90 80 70 60 50 40 30 20 10 0
ppm

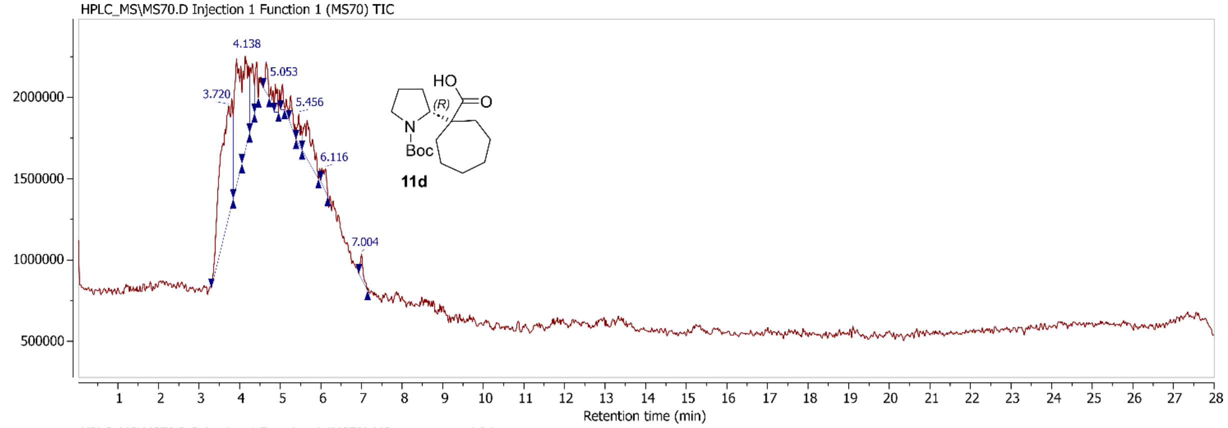
HPLC_MS\MS70.D Injection 1 Function 1 (MS70) TIC
4.138
5.053
3.720
5.456
6.116
7.004
2000000
1500000
1000000
500000
HO
O
(R)
N
Boc
11d
1 2 3 4 5 6 7 8 9 10 11 12 13 14 15 16 17 18 19 20 21 22 23 24 25 26 27 28
Retention time (min)

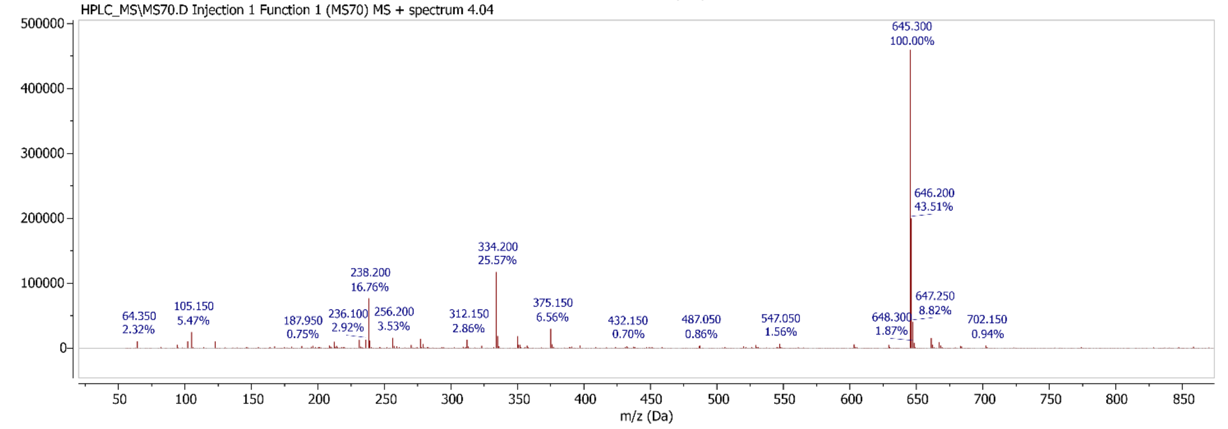
HPLC_MS\MS70.D Injection 1 Function 1 (MS70) MS + spectrum 4.04
500000
400000
300000
200000
100000
0
645.300
100.00%
646.200
43.51%
334.200
25.57%
238.200
16.76%
647.250
8.82%
375.150
6.56%
105.150
5.47%
64.350
2.32%
187.950
0.75%
236.100
2.92%
256.200
3.53%
312.150
2.86%
432.150
0.70%
487.050
0.86%
547.050
1.56%
648.300
1.87%
702.150
0.94%
50 100 150 200 250 300 350 400 450 500 550 600 650 700 750 800 850
m/z (Da)

References

1 González-Gómez, J. C.; Foubelo, F.; Yus, M. Preparation of enantioenriched homoallylic primary amines. *Org. Synth.* **2012**, *89*, 88–97.

2 Guo, T.; Yuan, B.-H.; Liu, W. Highly efficient asymmetric construction of novel indolines and tetrahydroquinoline derivatives *via* aza-Barbier/C−N coupling reaction. *Org. Biomol. Chem.* **2018**, *16*, 57–61.

3 In, J.; Lee, S.; Kwon, Y.; Kim, S. Divergent Total synthesis of the tricyclic marine alkaloids Lepadiformine, Fasicularin, and Isomers of Polycitorols by reagent-controlled diastereoselective reductive amination. *Chem. Eur. J.* **2014**, *20*, 17433–17442.

4 Li, W.-S.; Kuo, T.-S.; Hsieh, M.-C.; Tsai, M.-K.; Wu, P.-Y.; Wu, H.-L. Enantioselective rhodium-catalyzed allylation of aliphatic imines: synthesis of chiral c-aliphatic homoallylic amines. *Org. Lettt.* **2020**, *22*, 5675–5679.

5 (a) Liu, J.; Tu, J.-L.; Liu, F. Visible-light-promoted intramolecular α-allylation of aldehydes in the absence of sacrificial hydrogen acceptors. *Org. Lett.* **2020**, *22*, 7369–7372; (b) Tu, Y.-Q.; Yang, J.; Lu, K.; Li, C.; Zhao, Z.; Zhang, X.; Zhang, F.-M. Chiral 1,2,3-triazolium salt catalyzed asymmetric mono- and dialkylation of 2,5-diketopiperazines with the construction of tetrasubstituted carbon centers. *Angew. Chem. Int. Ed.* **2022**, *61*, e202114129.

6 Sirvent, A.; Hernández-Ibáñez, S.; Yus, M.; Foubelo, F. Pyrrolidine and indolizidine alkaloids from chiral *N*-*tert*-butanesulfinyl imines derived from 4-halobutanal. *Synthesis* **2021**, *53*, 1749–1759.

7 Lipschutz, B. H.; Hackmann, C. Conjugate addition reactions of allylic copper species derived from Grignard reagents: synthetic and spectroscopic aspects. *J. Org. Chem.* **1994**, *59*, 7437–7444.
